# Molecular Hybridization-Guided One-Pot Multicomponent Synthesis of Turmerone Motif-Fused 3,3′-Pyrrolidinyl-dispirooxindoles via a 1,3-Dipolar Cycloaddition Reaction

**DOI:** 10.3390/molecules22040645

**Published:** 2017-04-17

**Authors:** Bing Lin, Gen Zhou, Yi Gong, Qi-Di Wei, Min-Yi Tian, Xiong-Li Liu, Ting-Ting Feng, Ying Zhou, Wei-Cheng Yuan

**Affiliations:** 1 Guizhou Medicine Edicine Edible Plant Resources Research and Development Center, College of Pharmacy, Guizhou University, Guiyang 550025, China; nlin@gzu.edu.cn (B.L.); GenZhou@gzu.edu.cn (G.Z.); YiGong@gzu.edu.cn (Y.G.); Qi-DiWei@gzu.edu.cn (Q.-D.W.); Min-YiTian@gzu.edu.cn (M.-Y.T.); Ting-TingFeng@gzu.edu.cn (T.-T.F.); 2 Key Laboratory for Asymmetric Synthesis & Chirotechnology of Sichuan Province, Chengdu Institute of Organic Chemistry, Chinese Academy of Sciences, Chengdu 610041, China; yuanwc@cioc.ac.cn

**Keywords:** turmerone motif-fused 3,3′-pyrrolidinyl-dispirooxindoles, vicinal spiroquaternary stereocenters, 1,3-dipolar cycloaddition reaction, azomethine ylides, diastereoselectivity

## Abstract

Described herein is the development of a facile and efficient methodology for the synthesis of novel turmerone motif-fused 3,3′-pyrrolidinyl-dispirooxindoles **3**–**5** via a multicomponent 1,3-dipolar cycloaddition of dienones **2** with azomethine ylides (thermally generated in situfrom isatins and proline or thioproline or sarcosine). Products bearing four or three consecutive stereocenters consist of two oxindole moieties and a pyrrolidinyl core, including vicinal spiroquaternary stereocenters fused in one ring structure were smoothly obtained in high yields (up to 93% yield) with good diastereoselectivity (up to >20:1). Another valuable application of this method was for the design of new hybrid architectures for biological screening through the adequate fusion of these sub-units of turmerone and 3,3′-pyrrolidinyl-dispirooxindole, generating drug-like molecules.

## 1. Introduction

**Figure 1:**
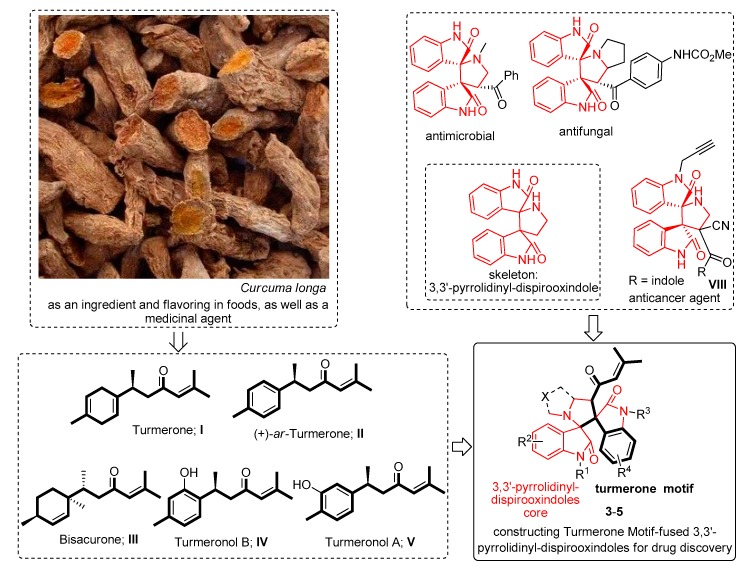
Design of turmerone motif-fused 3,3′-pyrrolidinyl-dispirooxindoles as a hybrid of these two motifs.

The close correlation between the specificity of biological activity and the complex, well-defined three-dimensionalshape of natural molecules has provided the impetus to develop novel strategies to stereoselectively access challenging target structures inherent in natural products or bioactive molecules [1,2,3,4,5]. Especially, 3,3′-pyrrolidinyl-dispirooxindoles have emerged as interesting targets owing to their complex polycyclic architecture. Some biologically active 3,3′-pyrrolidinyl-dispirooxindoles exhibit prominent bioactivities such as anticancer [6], antifungal [7], and antimicrobial [8] activities (Figure 1). Stereoselective construction of spirooxindoles is one of the most challenging tasks in catalytic organic reactions [9,10,11,12,13,14,15,16,17,18,19,20,21,22,23,24,25,26,27,28,29,30,31,32,33,34,35,36,37,38]. Generally, isatin and its derivatives have been employed as starting materials in 1,3-dipolar cycloaddition reactions yielding the spirooxindole core [39,40,41,42,43,44,45,46,47,48,49] due to the facile preparation of the corresponding azomethine ylides in the presence of α-amino acids [50,51,52,53,54,55,56], and a variety of 1,3-dipolarophiles such as α,β-unsaturated ketones [57,58,59] arylidenemalonodinitriles [60], α,β-unsaturated lactones [61] nitrostyrenes [62], acrylamides [63] and various other electron-deficient alkenes [45,64,65] have been documented. In view of their unique structural features and inspiring biological activities, the development of novel methods for the synthesis of 3,3′-pyrrolidinyl-dispirooxindoles is desirable (Figure 1).

On the other hand, the sesquiterpenes turmerone **I** [66,67,68,69], (*S*)-*ar*-turmerone **II** [66,67,68,69] and turmerone derivatives **III**–**V** [66,67,68,69] isolated from rhizomes of *Curcuma longa* [66,67,68,69] are reported to exhibit cytotoxic, anti-inflammatory, anti-cancer and anti-venom activity [66,67,68,69] (Figure 1). However, a close review of the literature data revealed that this biologically important turmerone scaffold has not yet been widely studied, particularly those turmerone motifs fused with other biological scaffolds.

**Scheme 1:**
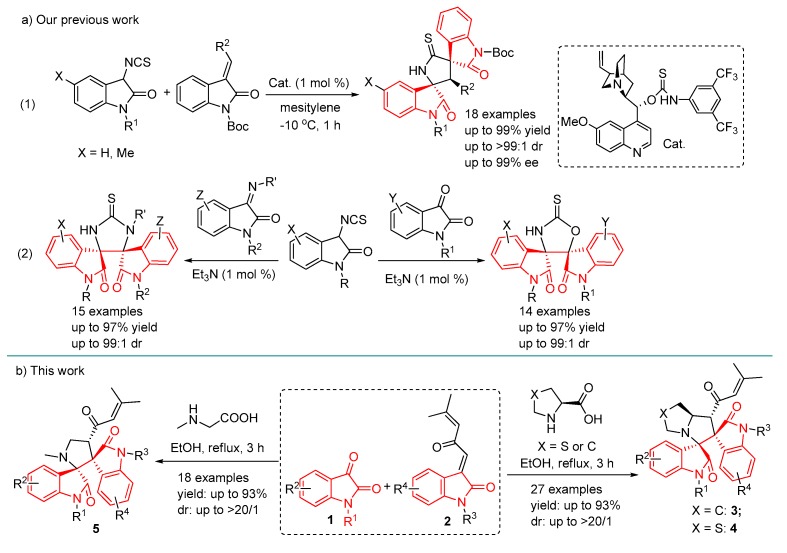
Construction of dispirooxindoles via 1,3-dipolar cycloaddition reaction [73].

In this context, we have been recently attracted by these 3,3′-pyrrolidinyl-dispirooxindoles due to their potential pharmaceutical applications. As a continuing effort to develop new methodology for the construction of complex dispirooxindoles (Scheme 1a) [70,71,72], we report herein a facile construction of novel turmerone motif-fused 3,3′-pyrrolidinyl-dispirooxindoles **3**–**5** via a multicomponent 1,3-dipolar cycloaddition reaction of dienones **2** with azomethine ylides (thermally generated in situ from isatins and proline or thioproline or sarcosine) (Scheme 1b).

## 2. Results and Discussion

In our initial endeavor, the dienone **2a** was prepared via a Knoevenagel condensation reaction of mesityl oxide with *N*-methylisatin [68]. The three-component 1,3-dipolar cycloaddition reaction of *N*-methylisatin **1a**, dienone **2a** and proline was investigated to substantiate the feasibility of the strategy under various reaction conditions, as shown in Table 1. We were pleased to find that the reaction led to the desired product **3aa** in moderate to good yields and *dr* values in different solvents (e.g., CH_3_CN, DCE, EtOAc, EtOH, THF, H_2_O and toluene). Finally, EtOH was found to be the best choice among all the solvents with respect to the stereoselectivity and yield (Table 1, entries 1–7). The reaction also occurs at 40 °C but extended reaction time (48 h) is required and isolated yield of product **3aa** is lower (52%) (Table 1, entry 8). Increasing the amount of EtOH from 6.0 mL to 10.0 mL had a positive effect on both the *dr* value and yield of **3aa** albeit with shortened reaction time, probably because it increased solubility of the substrates **1a**, **2a**, proline and product **3aa** in this reaction system (Table 1, entry 9). Decreasing the amount of proline led to the desired product **3aa** in the relatively lower yield (72%), along with some starting materials remained (Table 1, entry 11). Thus, the optimal reaction conditions we established were: isatin **1a** (0.6 mmol), dienone **2a** (0.4 mmol), proline (0.8 mmol) in 10.0 mL of EtOH at reflux for 3 h.

With the optimized reaction conditions in hands, we next turned our interest to the reaction scope, and the results were summarized in Table 2, Table 3 and Table 4. Proline was first used as a standard substrate to probe the reactivity of different isatins **1** and dienones **2** in this reaction. All of the reactions proceeded smoothly under the optimal conditions, producing the desired products **3** in moderate to good yields with good diastereoselectivities (Table 2, compounds **3aa**–**3le**). Interestingly, electron-rich (Table 2, compounds **3da**, **3ha**, **3ka** and **3dd**) and electron-poor (Table 2, compounds **3ea**, **3fa**, **3ga**, **3ia**, **3ja**, **3la**, **3fd**, **3if** and **3le**) substituents on the benzomoiety of isatins **1** were totally tolerated under the conditions. In addition, significant structural variation in the benzomoiety of dienones **2** could be accommodated in this reaction, producing the desired products **3** in moderate to good yields, regardless of the electronic nature of the substituents (Table 2, compounds **3ab**–**3le**).

The generality of the reaction was further demonstrated by using a thioproline as a standard substrate (Table 3). Significant structural variation in the benzo-moiety of isatins **1** could be accommodated in this reaction, producing the desired products **4** in moderate to good yields with excellent diastereoselectivities (Table 3, products **4aa**–**4ma**). In addition, comparing the results reported in Table 2 and Table 3, a trend becomes evident: the yields for the reaction with proline as the substrate are higher than those obtained with thioproline, indicating that proline is more reactive than thioproline in this reaction. On the other hand, the stereoselectivities recorded with thioproline are very high regardless of the employed substrates.

To further substantiate generality of the reaction, sarcosine was also used as a substrate to probe the reactivity of different isatins **1** under the optimal conditions (Table 4, products **5aa**–**5oc**). It was found that, regardless of electron-donating substituents **1d**, **1m** and **1p** or electron-withdrawing substituents **1e**–**1g**, **1i**, **1j** and **1n**–**1o** on the aromatic ring of isatins **1** (Table 4, entries 1–13), the corresponding products **5** could be successfully obtained in good yields with excellent diastereoselectivities.

**Figure 2:**
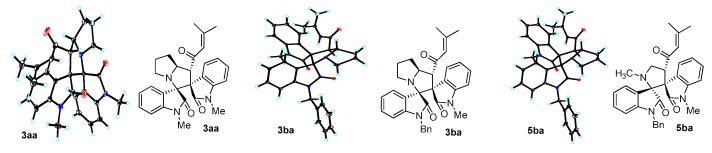
X-ray crystallographic structures of **3aa**, **3ba** and **5ba**.

All the targets piropyrrolidine oxindoles **3**–**5** were characterized by nuclear magnetic resonance (NMR) spectroscopy and mass spectrometry, and their structures were further confirmed by X-ray crystallographic studies of single crystals of **3aa**, **3ba** and **5ba** [73] (Figure 2).

**Scheme 2:**
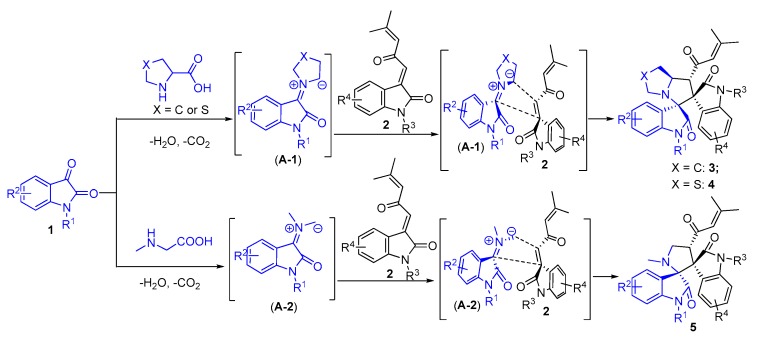
Plausible mechanism of the cycloaddition reaction providing 3,3′-pyrrolidinyl-dispirooxindoles **3**–**5**.

In view of the above data and the previously disclosed similar systems in the literature [10], we tentatively propose a working model as shown in Scheme 2. The reactions of isatins (**1**) and proline, thioproline or sarcosine led to the azomethine ylides (**A**) via a decarboxylation process. The ylides (**A**) formed could be added to the dipolarophile dienones **2** from the less hindered side thus to give the expected cycloaddition products **3**–**5** (Scheme 2).

**Scheme 3:**
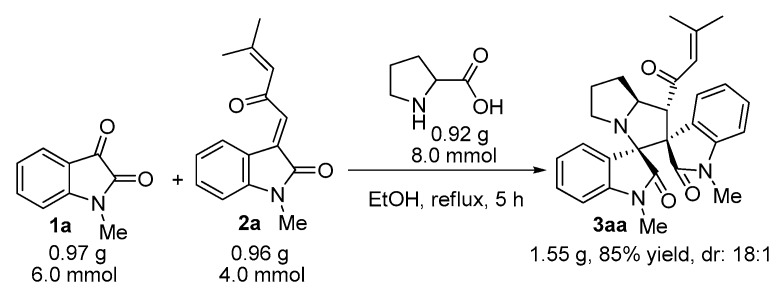
The 1,3-dipolar cycloaddition reaction on a gram scale.

According to the reaction mechanism as shown in scheme 2, if l- or d-proline was used in this cycloaddition, the racemic products would be obtained. The regiochemical outcome of the cycloaddition was further confirmed by single crystal X-ray structures of the cycloadducts **3aa**, **3ba** and **5ba**. The significance and the high efficacy of the current protocol were demonstrated by a gram-scale synthesis of **3aa**. The 1,3-dipolar cycloaddition reaction between **1a**, **2a** and proline proceeded cleanly on a 4.0 mmol scale (0.96 g of **2a**) in 100 mL EtOH at reflux for 5 h. As outlined in Scheme 3, the corresponding adduct **3aa** was obtained smoothly in 85% yield, which was similar to that observed in the previous investigation (entry 9, Table 1).

Subsequently, to further demonstrate the potential activities of these synthesized turmerone motif-fused 3,3′-pyrrolidinyl-dispirooxindoles, following the literature precedent by Mosmann and coworkers [74,75] with minor modification (Alley et al.) [74,75], we evaluated in vitro anticancer (human leukemia cells K562) activities of the newly synthesized 10 compounds **3le**, **3fa**, **3la**, **3ia**, **3fd**, **4da**, **5ia**, **5ba**, **5oa** and **5pa** by the MTT-based assay using the commercially available broad-spectrum anticancer drug of cisplatin as a positive control, and their IC_50_ concentration were depicted in Table 5. The results demonstrated that the newly synthesized 10 compounds **3le**, **3fa**, **3la**, **3ia**, **3fd**, **4da**, **5ia**, **5ba**, **5oa** and **5pa** showed considerable cytotoxicities to the cell lines K562, and showed equipotent potent than the positive control of cisplatin. The results also indicated that synthesized turmerone motif-fused 3,3′-pyrrolidinyl-dispirooxindoles may be useful leads for further biological screenings.

## 3. Experimental Section

### 3.1. General

The ^1^H- and ^13^C-NMRspectra were recorded on Bruker Avance DMX 400 MHz or 500 M NMR spectrometers in CDCl_3_ using TMS as internal standard. Chemical shifts were reported as δ values (ppm). High-resolution mass spectra (HRMS-ESI) were obtained on a Micro^TM^ Q-TOF Mass Spectrometer. Melting points were uncorrected and recorded on an Electothermal 9100 digital melting point apparatus. Reagents were purchased from commercial sources and were used as received unless mentioned otherwise. Reactions were monitored by thin layer chromatography using silica gel GF_254_ plates. Column chromatography was performed on silica gel (300–400 mesh).

### 3.2. General Experimental Procedures for the Synthesis of Compounds ***3***–***5***

A solution of isatins **1** (0.6 mmol), dienones **2** (0.4 mmol) and proline, thioproline or sarcosine (0.8 mmol) in the 10.0 mL of EtOH at reflux for 3 h. After completion of the reaction, as indicated by TLC, the removal of solvent and purification by flash column chromatography (hexane/EtOAc = 5:1~3:1) were carried out to furnish the corresponding products **3**–**5**.

### 3.3. Characterization Data of Compounds ***3***–***5***

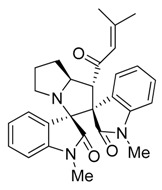



**3aa**: Light orange solid, m.p. 176.5–177.8 °C; yield 89%, 20:1 *dr*; ^1^H-NMR (CDCl_3_) δ: 1.43 (s, 3H), 1.61 (s, 3H), 2.05–2.10 (m, 2H), 2.21–2.29 (m, 2H), 2.47–2.53 (m, 1H), 2.66–2.71 (m, 1H), 2.88 (s, 3H), 3.18 (s, 3H), 4.58–4.63 (m, 2H), 5.75 (s, 1H), 6.39–6.41 (m, 1H), 6.51–6.56 (m, 2H), 6.60 (d, *J* = 8.0 Hz, 1H), 7.03–7.13 (m, 2H), 7.20–7.25 (m, 1H), 7.77 (d, *J* = 7.6 Hz, 1H); ^13^C-NMR (CDCl_3_) δ: 20.0, 26.0, 26.2, 27.1, 30.9, 31.3, 47.3, 59.0, 65.0, 67.2, 107.4, 121.6, 122.3, 123.1, 125.1, 125.5, 127.4, 128.8, 129.2, 143.3, 143.8, 154.4, 172.9, 177.1, 196.9; HRMS (ESI-TOF) *m*/*z*: Calcd. for C_28_H_29_N_3_NaO_3_ [M + Na]^+^: 478.2107; Found: 478.2109.

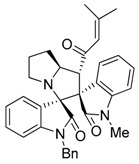



**3ba**: Light orange solid, m.p. 112.2–113.6 °C; yield 83%, 12:1 *dr*; ^1^H-NMR (CDCl_3_) δ: 1.40 (s, 3H), 1.57 (s, 3H), 2.02–2.08 (m, 2H), 2.19–2.26 (m, 2H), 2.50–2.55 (m, 1H), 2.66–2.69 (m, 1H), 2.87 (s, 3 H), 4.57–4.63 (m, 2H), 4.76 (d, *J* = 12.4 Hz, 1H), 4.94 (d, *J* = 12.4 Hz, 1H), 5.74 (s, 1H), 6.34–6.37 (m, 1H), 6.41–6.44 (m, 1H), 6.50 (d, *J* = 6.0 Hz, 1H), 6.83–6.87 (m, 1H), 7.05–7.08 (m, 1H), 7.15–7.19 (m, 2H), 7.23–7.26 (m, 2H), 7.30–7.32 (m, 2H), 7.73 (d, *J* = 6.0 Hz, 1H); ^13^C-NMR (CDCl_3_) δ: 20.0, 26.2, 27.0, 30.8, 31.4, 44.0, 47.3, 59.1, 65.0, 67.0, 107.4, 108.6, 122.2, 123.1, 127.3, 127.4, 127.5, 128.6, 135.9, 143.0, 143.3, 154.4, 172.9, 177.0, 196.9; HRMS (ESI-TOF) *m*/*z*: Calcd. for C_34_H_33_N_3_NaO_3_ [M + Na]^+^: 554.2420; Found: 554.2423.

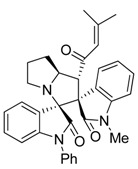



**3ca**: Light orange solid, m.p. 117.2–118.6 °C; yield 61%, 12:1 *dr*; ^1^H-NMR (CDCl_3_) δ: 1.47 (s, 3H), 1.64 (s, 3H), 2.08–2.16 (m, 2H), 2.24–2.31 (m, 2H), 2.66–2.71 (m, 1H), 2.82–2.85 (m, 1H), 2.96 (s, 3H), 4.66–4.68 (m, 2H), 5.80 (s, 1H), 6.49–6.51 (m, 1H), 6.56–6.63 (m, 3H), 6.99–7.03 (m, 1H), 7.14–7.17 (m, 1H), 7.24–7.28 (m, 1H), 7.38–7.41 (m, 1H), 7.52–7.54 (m, 4H), 7.83 (d, *J* = 5.6 Hz, 1H); ^13^C-NMR (CDCl_3_) *δ*: 20.0, 26.2, 27.0, 30.8, 31.2, 47.4, 58.9, 65.1, 67.5, 107.4, 108.6, 121.8, 123.1, 125.7, 126.9, 127.4, 128.0, 128.9, 129.5, 143.4, 143.6, 154.3, 172.9, 176.7, 196.9; HRMS (ESI-TOF) *m*/*z*: Calcd. for C_33_H_31_N_3_NaO_3_ [M + Na]^+^: 540.2263; Found: 540.2265.

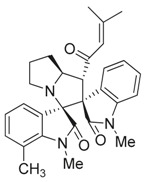



**3da**: Light orange solid, m.p. 185.4–186.8 °C; yield 72%, 13:1 *dr*; ^1^H-NMR (CDCl_3_) δ: 1.45 (s, 3H), 1.63 (s, 3H), 2.05–2.11 (m, 2H), 2.23–2.33 (m, 2H), 2.46 (s, 3H), 2.48–2.53 (m, 1H), 2.69–2.72 (m, 1H), 2.93 (s, 3H), 3.49 (s, 3H), 4.58–4.65 (m, 2H), 5.76 (s, 1H), 6.36 (d, *J* = 5.6 Hz, 1H), 6.41–6.44 (m, 1H), 6.56 (d, *J* = 6.4 Hz, 1H), 6.80 (d, *J* = 6.0 Hz, 1H), 7.10–7.13 (m, 1H), 7.21–7.24 (m, 1H), 7.77 (d, *J* = 6.0 Hz, 1H); ^13^C-NMR (CDCl_3_) δ: 19.2, 20.0, 26.3, 27.1, 29.5, 30.8, 31.2, 47.3, 59.2, 64.9, 67.4, 107.4, 118.7, 121.3, 122.2, 123.1, 123.6, 127.5, 128.8, 133.0, 141.5, 143.3, 154.2, 173.0, 177.8, 197.0; HRMS (ESI-TOF) *m*/*z*: Calcd. for C_29_H_31_N_3_NaO_3_ [M + Na]^+^: 492.2263; Found: 492.2265.

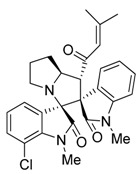



**3ea**: Light orange solid, m.p. 106.8–108.5 °C; yield 92%, 20:1 *dr*; ^1^H-NMR (CDCl_3_) δ: 1.42 (s, 3H), 1.60 (s, 3H), 2.06–2.08 (m, 2H), 2.22–2.25 (m, 2 H), 2.46–2.49 (m, 1H), 2.65–2.68 (m, 1H), 2.93 (s, 3H), 3.56 (s, 3H), 4.56–4.59 (m, 2H), 5.72 (s, 1H), 6.39–6.43 (m, 2H), 6.55 (d, *J* = 8.0 Hz, 1H), 6.95–6.98 (m, 1H), 7.07–7.11 (m, 1H), 7.18–7.25 (m, 1H), 7.72–7.74 (m, 1H); ^13^C-NMR (CDCl_3_) δ: 20.1, 26.4, 27.2, 29.6, 30.9, 31.4, 47.4, 59.2, 65.1, 67.4, 107.7, 114.7, 122.2, 122.4, 123.1, 124.2, 126.2, 127.5, 128.1, 129.1, 131.6, 139.7, 143.4, 154.7, 172.8, 177.5, 196.7; HRMS (ESI-TOF) *m*/*z*: Calcd. for C_28_H_28_ClN_3_NaO_3_ [M + Na]^+^: 512.1717; Found: 512.1717.

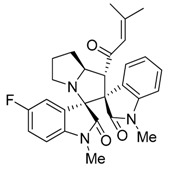



**3fa**: Light orange solid, m.p. 204.3–206.5 °C; yield 82%, 10:1 *dr*; ^1^H-NMR (CDCl_3_, 500 MHz) δ: 1.40 (s, 3H), 1.57 (s, 3H), 2.02–2.06 (m, 2H), 2.17–2.24 (m, 2H), 2.41–2.47 (m, 1H), 2.63–2.67 (m, 1H), 2.86 (s, 3H), 3.12 (s, 3H), 4.50–4.57 (m, 2H), 5.70 (s, 1H), 6.09–6.12 (m, 1H), 6.46–6.49 (m, 1H), 6.54 (d, *J* = 7.5 Hz, 1H), 6.69–6.73 (m, 1H), 7.07–7.10 (m, 1H), 7.18–7.22 (m, 1H), 7.70 (d, *J* = 7.0 Hz, 1H); ^13^C-NMR (CDCl_3_, 125 MHz) δ: 20.0, 26.1, 26.2, 27.1, 30.8, 31.2, 47.3, 59.0, 65.1, 67.1, 107.6, 107.7, 113.7 (d, *J_CF_* = 25.0 Hz), 115.3 (d, *J_CF_* = 23.8 Hz), 122.4, 123.0, 126.1, 127.1, 127.2, 129.2, 139.7, 143.2, 154.6, 158.3 (d, *J_CF_* = 238.8 Hz), 172.6, 176.8, 196.6; HRMS (ESI-TOF) *m*/*z*: Calcd. for C_28_H_28_FN_3_NaO_3_ [M + Na]^+^: 496.2012; Found: 496.2015.

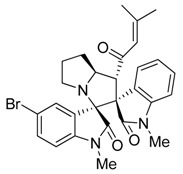



**3ga**: Light orange solid, m.p. 243.3–245.5 °C; yield 85%, 19:1 *dr*; ^1^H-NMR (CDCl_3_) δ: 1.48 (s, 3H), 1.64 (s, 3H), 2.08–2.12 (m, 2H), 2.23–2.29 (m, 2H), 2.48–2.53 (m, 1H), 2.72–2.75 (m, 1H), 2.93 (s, 3H), 3.18 (s, 3H), 4.58–4.61 (m, 2H), 5.77 (s, 1H), 6.49–6.51 (m, 2H), 6.62 (d, *J* = 6.4 Hz, 1H), 7.16–7.21 (m, 2H), 7.27–7.31 (m, 1H), 7.76 (d, *J* = 6.0 Hz, 1H); ^13^C-NMR (CDCl_3_) δ: 20.0, 26.1, 26.2, 27.1, 30.8, 31.3, 47.3, 58.9, 65.2, 67.2, 107.7, 108.7, 114.3, 122.4, 122.9, 127.2, 128.8, 129.2, 131.9, 142.7, 143.2, 154.6, 172.6, 176.5, 196.6; HRMS (ESI-TOF) *m*/*z*: Calcd. for C_28_H_28_BrN_3_NaO_3_ [M + Na]^+^: 556.1212; Found: 556.1214.

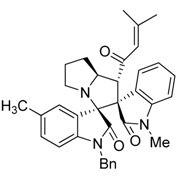



**3ha**: Light orange solid, m.p. 204.4–205.6 °C; yield 90%, >20:1 *dr*; ^1^H-NMR (CDCl_3_) δ: 1.41 (s, 3H), 1.57 (s, 3H), 1.80 (s, 3H), 2.03–2.08 (m, 2H), 2.18–2.29 (m, 2H), 2.51–2.56 (m, 1H), 2.66–2.70 (m, 1H), 2.87 (s, 3H), 4.56–4.63 (m, 2H), 4.75 (d, *J* = 12.8 Hz, 1H), 4.91 (d, *J* = 12.4 Hz, 1H), 5.74 (s, 1H), 6.12 (s, 1H), 6.23 (d, *J* = 6.4 Hz, 1H), 6.49 (d, *J* = 6.0 Hz, 1H), 6.64 (d, *J* = 6.0 Hz, 1H), 7.06–7.09 (m, 1H), 7.15–7.19 (m, 2H), 7.22–7.26 (m, 2H), 7.30 (d, *J* = 5.6 Hz, 2H), 7.73 (d, *J* = 5.6 Hz, 1H); ^13^C-NMR (CDCl_3_) δ: 20.0, 20.7, 26.2, 27.0, 30.8, 31.4, 44.1, 47.4, 59.1, 65.2, 67.0, 107.4, 108.2, 122.1, 123.1, 126.5, 127.3, 127.4, 127.5, 128.5, 128.7, 129.2, 130.9, 136.1, 154.3, 173.0, 176.8, 197.0; HRMS (ESI-TOF) *m*/*z*: Calcd. for C_35_H_35_N_3_NaO_3_ [M + Na]^+^: 568.2576; Found: 568.2575.

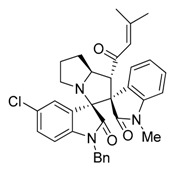



**3ia**: Light orange solid, m.p. 161.1–162.8 °C; yield 88%, 12:1 *dr*; ^1^H-NMR (CDCl_3_) δ: 1.47 (s, 3H), 1.64 (s, 3H), 2.08–2.15 (m, 2H), 2.24–2.29 (m, 2H), 2.53–2.59 (m, 1H), 2.73–2.77 (m, 1H), 2.95 (s, 3H), 4.61–4.65 (m, 2H), 4.81 (d, *J* = 15.6 Hz, 1H), 4.97 (d, *J* = 15.6 Hz, 1H), 5.79 (s, 1H), 6.30–6.35 (m, 2H), 6.59–6.62 (m, 1H), 6.85–6.88 (m, 1H), 7.13–7.17 (m, 1H), 7.22–7.35 (m, 6H), 7.75–7.77 (m, 1H); ^13^C-NMR (CDCl_3_) δ: 20.2, 26.3, 27.2, 30.9, 31.5, 44.3, 47.4, 59.2, 65.3, 67.1, 107.9, 109.6, 123.1, 126.2, 127.1, 127.2, 127.3, 127.6, 127.7, 128.8, 129.1, 129.3, 135.6, 154.8, 172.8, 176.7, 196.7; HRMS (ESI-TOF) *m*/*z*: Calcd. for C_34_H_32_ClN_3_NaO_3_ [M + Na]^+^: 588.2030; Found: 588.2032.

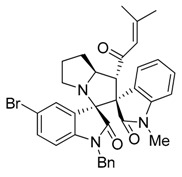



**3ja**: Light orange solid, m.p. 180.2–182.1 °C; yield 88%, >20:1 *dr*; ^1^H-NMR (CDCl_3_) δ: 1.47 (s, 3H), 1.63 (s, 3H), 2.09–2.14 (m, 2H), 2.25–2.29 (m, 2H), 2.54–2.58 (m, 1H), 2.72–2.79 (m, 1H), 2.95 (s, 3H), 4.60–4.62 (m, 2H), 4.79 (d, *J* = 15.6 Hz, 1H), 4.96 (d, *J* = 16.0 Hz, 1H), 5.78 (s, 1H), 6.26 (d, *J* = 8.4 Hz, 1H), 6.47 (d, *J* = 2.4 Hz, 1H), 6.59–6.62 (m, 1H), 6.99–7.02 (m, 1H), 7.13–7.17 (m, 1H), 7.23–7.34 (m, 6H), 7.73–7.76 (m, 1H); ^13^C-NMR (CDCl_3_) δ: 20.2, 26.3, 27.2, 30.9, 31.5, 44.2, 47.4, 59.1, 65.3, 67.2, 107.9, 110.1, 104.6, 122.5, 123.1, 127.3, 127.6, 127.7, 128.8, 129.0, 129.3, 135.6, 154.8, 172.8, 176.6, 196.7; HRMS (ESI-TOF) *m*/*z*: Calcd. for C_34_H_32_BrN_3_NaO_3_ [M + Na]^+^: 632.1525; Found: 632.1528.

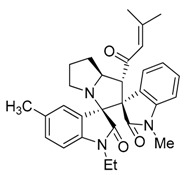



**3ka**: Light orange solid, m.p. 124.3–126.5 °C; yield 80%, 8:1 *dr*; ^1^H-NMR (CDCl_3_) δ: 1.24–1.27 (m, 3H), 1.47 (s, 3H), 1.62 (s, 3H), 1.92 (s, 3H), 2.09–2.13 (m, 2H), 2.25–2.28 (m, 2H), 2.52–2.56 (m, 1H), 2.69–2.73 (m, 1H), 2.90 (s, 3H), 3.63–3.67 (m, 1H), 3.80–3.85 (m, 1H), 4.61–4.65 (m, 2H), 5.78 (s, 1H), 6.20 (s, 1H), 6.51–6.57 (m, 2H), 6.84–6.87 (m, 1H), 7.13–7.16 (m, 1H), 7.23–7.27 (m, 1H), 7.78 (d, *J* = 6.0 Hz, 1H); ^13^C-NMR (CDCl_3_) δ: 12.4, 19.9, 20.7, 26.1, 27.0, 30.8, 31.3, 34.5, 47.3, 58.9, 65.2, 67.1, 107.0, 107.3, 122.0, 123.1, 126.6, 127.3, 128.7, 129.2, 130.6, 140.3, 143.3, 154.1, 172.8, 176.3, 197.1; HRMS (ESI-TOF) *m*/*z*: Calcd. for C_30_H_33_N_3_NaO_3_ [M + Na]^+^: 506.2420; Found: 506.2423.

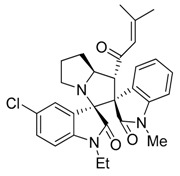



**3la**: Light orange solid, m.p. 206.6–207.3 °C; yield 71%, 8:1 *dr*; ^1^H-NMR (CDCl_3_) δ: 1.23–1.27 (m, 3H), 1.47 (s, 3H), 1.64 (s, 3H), 2.08–2.13 (m, 2H), 2.22–2.28 (m, 2H), 2.48–2.54 (m, 1H), 2.70–2.75 (m, 1H), 2.93 (s, 3H), 3.62–3.68 (m, 1H), 3.80–3.86 (m, 1H), 4.58–4.62 (m, 2H), 5.77 (s, 1H), 6.37 (s, 1H), 6.56 (d, *J* = 8.0 Hz, 1H), 6.61 (d, *J* = 8.0 Hz, 1H), 7.03–7.05 (m, 1H), 7.14–7.18 (m, 1H), 7.26–7.30 (m, 1H), 7.76 (d, *J* = 8.0 Hz, 1H); ^13^C-NMR (CDCl_3_) δ: 12.3, 20.0, 26.1, 27.0, 30.8, 31.3, 34.7, 47.2, 58.9, 65.1, 67.0, 107.6, 108.2, 122.3, 123.0, 126.2, 126.7, 127.2, 128.9, 129.1, 141.3, 143.2, 154.5, 172.5, 176.1, 196.6; HRMS (ESI-TOF) *m*/*z*: Calcd. for C_29_H_30_ClN_3_NaO_3_ [M + Na]^+^: 526.1873; Found: 526.1876.

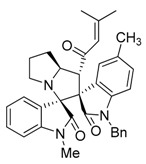



**3ab**: Light orange solid, m.p. 188.3–190.5 °C; yield 71%, 15:1 *dr*; ^1^H-NMR (CDCl_3_) δ: 1.50 (s, 3H), 1.64 (s, 3H), 2.11–2.18 (m, 2H), 2.25–2.36 (m, 2H), 2.40 (s, 3H), 2.54–2.61 (m, 1H), 2.75–2.79 (m, 1H), 3.17 (s, 3H), 4.14 (d, *J* = 16.0 Hz, 1H), 4.63–4.71 (m, 2H), 5.20 (d, *J* = 16.0 Hz, 1H), 5.82 (s, 1H), 6.20 (d, *J* = 7.6 Hz, 1H), 6.34–6.40 (m, 3H), 6.59–6.63 (m, 1H), 6.67 (d, *J* = 7.6 Hz, 1H), 6.90 (d, *J* = 7.6 Hz, 1H), 7.01–7.05 (m, 2H), 7.08–7.12 (m, 1H), 7.19–7.23 (m, 1H), 7.60 (s, 1H); ^13^C-NMR (CDCl_3_) δ: 20.0, 21.4, 26.0, 27.1, 30.9, 31.4, 43.1, 47.5, 58.7, 65.5, 67.3, 77.8, 107.5, 108.3, 121.7, 123.1, 125.7, 125.8, 126.9, 127.9, 128.5, 129.1, 129.3, 131.7, 135.0, 154.1, 172.9, 177.0, 197.2; HRMS (ESI-TOF) *m*/*z*: Calcd. for C_35_H_35_N_3_NaO_3_ [M + Na]^+^: 568.2576; Found: 568.2576.

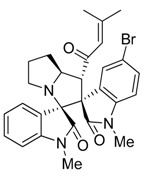



**3ac**: Light orange solid, m.p. 97.3–99.1 °C; yield 68%, 9:1 *dr*; ^1^H-NMR (CDCl_3_) δ: 1.58 (s, 3H), 1.67 (s, 3H), 2.08–2.15 (m, 2H), 2.22–2.34 (m, 2H), 2.45–2.52 (m, 1H), 2.69–2.73 (m, 1H), 2.88 (s, 3H), 3.17 (s, 3H), 4.43–4.49 (m, 1H), 4.65 (d, *J* = 8.8 Hz, 1H), 5.79 (s, 1H), 6.43–6.46 (m, 2H), 6.58–6.63 (m, 2H), 7.07–7.11 (m, 1H), 7.36–7.38 (m, 1H), 7.87 (s, 1H); ^13^C-NMR (CDCl_3_) δ: 20.4, 26.2, 26.4, 27.4, 30.9, 31.6, 47.4, 59.3, 65.2, 67.1, 107.6, 109.0, 115.0, 121.9, 122.9, 124.8, 125.5, 129.5, 130.0, 131.7, 142.7, 143.9, 155.2, 172.5, 176.9; HRMS (ESI-TOF) *m*/*z*: Calcd. for C_28_H_28_BrN_3_NaO_3_ [M + Na]^+^: 556.1212; Found: 556.1215.

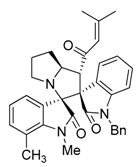



**3dd**: Light orange solid, m.p. 165.3–166.6 °C; yield 89%, >20:1 *dr*; ^1^H-NMR (CDCl_3_) δ: 1.46 (s, 3H), 1.63 (s, 3H), 2.08–2.16 (m, 2H), 2.23–2.31 (m, 1H), 2.33–2.38 (m, 1H), 2.44 (s, 3H), 2.51–2.58 (m, 1H), 2.71–2.76 (m, 1H), 3.42 (s, 3H), 4.14 (d, *J* = 16.0 Hz, 1H), 4.63–4.69 (m, 2H), 5.25 (d, *J* = 16.4 Hz, 1H), 5.81 (s, 1H), 6.28–6.31 (m, 2H), 6.36 (d, *J* = 7.6 Hz, 2H), 6.42–6.46 (m, 1H), 6.90–6.93 (m, 1H), 7.01–7.11 (m, 5H), 7.74–7.76 (m, 1H); ^13^C-NMR (CDCl_3_) δ: 19.3, 20.1, 27.2, 29.6, 31.0, 31.5, 43.2, 47.6, 58.8, 65.5, 67.5, 108.5, 119.1, 121.6, 122.3, 123.2, 123.8, 125.8, 127.1, 127.5, 128.6, 128.9, 135.1, 154.4, 173.1, 177.9, 197.2; HRMS (ESI-TOF) *m*/*z*: Calcd. for C_35_H_35_N_3_NaO_3_ [M + Na]^+^: 568.2576; Found: 568.2577.

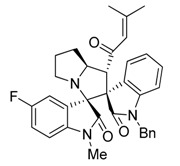



**3fd**: Light orange solid, m.p. 204.3–205.8 °C; yield 93%, >20:1 dr; ^1^H-NMR (CDCl_3_) δ: 1.50 (s, 3H), 1.64 (s, 3H), 2.09–2.16 (m, 2H), 2.23–2.35 (m, 2H), 2.50–2.57 (m, 1H), 2.72–2.76 (m, 1H), 3.13 (s, 3H), 4.20 (d, *J* = 16.0 Hz, 1H), 4.59–4.69 (m, 2H), 5.14 (d, *J* = 16.0 Hz, 1H), 5.82 (s, 1H), 6.07–6.10 (m, 1H), 6.36–6.38 (m, 1H), 6.46 (d, *J* = 7.2 Hz, 2H), 6.53–6.56 (m, 1H), 6.84–6.89 (m, 1H), 7.03–7.15 (m, 5H), 7.73–7.76 (m, 1H); ^13^C-NMR (CDCl_3_) δ: 20.2, 26.3, 27.2, 31.0, 31.5, 43.3, 47.6, 58.8, 65.6, 67.2, 77.9, 107.8, 107.9, 108.9, 114.0 (d, *J_CF_* = 26.0 Hz), 115.5 (d, *J_CF_* = 24.0 Hz), 122.5, 123.1, 126.1, 126.4, 127.2, 127.3, 128.7, 129.3, 135.1, 140.0, 142.5, 155.0, 158.6 (d, *J_CF_* = 239.0 Hz), 172.8, 176.8, 196.8; HRMS (ESI-TOF) *m*/*z*: Calcd. for C_34_H_32_FN_3_NaO_3_ [M + Na]^+^: 572.2325; Found: 572.2327.

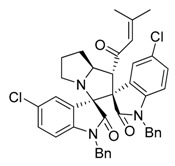



**3if**: Light orange solid, m.p. 177.3–178.9 °C; yield 67%, 10:1 *dr*; ^1^H-NMR (CDCl_3_) δ: 1.67 (s, 3H), 1.71 (s, 3H), 2.11–2.23 (m, 2H), 2.28–2.41 (m, 2H), 2.55–2.62 (m, 1H), 2.78–2.83 (m, 1H), 4.27 (d, *J* = 16.0 Hz, 1H), 4.49–4.56 (m, 1H), 4.75–4.80 (m, 2H), 4.93 (d, *J* = 15.6 Hz, 1H), 5.17 (d, *J* = 16.0 Hz, 1H), 5.89 (s, 1H), 6.31–6.36 (m, 3H), 6.41 (d, *J* = 8.0 Hz, 2H), 6.95–7.00 (m, 3H), 7.06–7.15 (m, 5H), 7.23–7.25 (m, 2H), 7.72 (s, 1H); ^13^C-NMR (CDCl_3_) δ: 20.6, 27.5, 30.9, 31.8, 43.5, 44.3, 47.5, 59.1, 65.7, 67.0, 109.9, 110.0, 122.7, 126.0, 127.1, 127.3, 127.4, 127.5, 127.6, 127.9, 128.7, 128.8, 134.5, 135.1, 156.0, 172.4, 176.5, 196.3; HRMS (ESI-TOF) *m*/*z*: Calcd. for C_40_H_35_Cl_2_N_3_NaO_3_ [M + Na]^+^: 698.1953; Found: 698.1954.

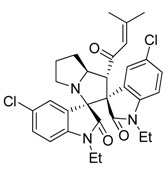



**3le**: Light orange solid, m.p. 148.3–150.5 °C; yield 84%, 14:1 *dr*; ^1^H-NMR (CDCl_3_) δ: 0.73–0.77 (m, 3H), 1.20–1.24 (m, 3H), 1.63–1.65 (m, 6H), 2.02–2.15 (m, 2H), 2.21–2.32 (m, 2H), 2.44–2.51 (m, 1H), 2.70–2.74 (m, 1H), 3.27–3.32 (m, 1H), 3.51–3.60 (m, 2H), 3.81–3.87 (m, 1H), 4.46–4.52 (m, 1H), 4.59 (d, *J* = 7.6 Hz, 1H), 5.80 (s, 1H), 6.23 (s, 1H), 6.50–6.53 (m, 2H), 7.01–7.03 (m, 1H), 7.23–7.25 (m, 1H), 7.67 (d, *J* = 1.6 Hz, 1H); ^13^C-NMR (CDCl_3_) δ: 12.1, 12.4, 20.5, 27.4, 30.9, 31.7, 34.7, 34.8, 47.4, 58.4, 65.6, 67.1, 108.3, 108.6, 122.8, 126.1, 126.9, 127.4, 127.5, 128.9, 129.1, 141.1, 141.4, 155.9, 171.6, 175.9, 196.3; HRMS (ESI-TOF) *m*/*z*: Calcd. for C_30_H_31_Cl_2_N_3_NaO_3_ [M + Na]^+^: 574.1640; Found: 574.1638.

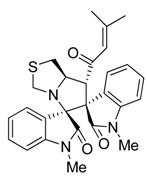



**4aa**: Light orange solid, m.p. 198.2–200.1 °C; yield 72%, >20:1 *dr*; ^1^H-NMR (CDCl_3_) δ: 1.56 (s, 3H), 1.58 (s, 3H), 2.89 (s, 3H), 2.94 (s, 3H), 2.95–2.98 (m, 1H), 3.35–3.39 (m, 1H), 3.63 (d, *J* = 6.4 Hz, 1H), 3.84 (d, *J* = 7.2 Hz, 1H), 3.91 (d, *J* = 7.2 Hz, 1H), 5.26–5.31 (m, 1H), 5.52 (s, 1H), 6.61 (d, *J* = 6.4 Hz, 1H), 6.67 (d, *J* = 6.0 Hz, 1H), 7.01–7.04 (m, 1H), 7.09–7.13 (m, 1H), 7.19–7.22 (m, 1H), 7.30–7.33 (m, 1H), 7.62–7.64 (m, 1H), 7.73–7.74 (m, 1H); ^13^C-NMR (CDCl_3_) δ: 20.3, 26.2, 26.4, 27.1, 36.1, 50.4, 62.0, 65.9, 67.6, 76.4, 107.3, 107.8, 121.5, 122.0, 122.9, 123.2, 124.7, 128.5, 128.7, 129.0, 130.0, 142.4, 143.5, 156.1, 173.8, 174.2; HRMS (ESI-TOF) *m*/*z*: Calcd. for C_27_H_27_N_3_NaO_3_S [M + Na]^+^: 496.1671; Found: 496.1673.

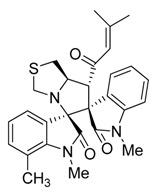



**4da**: Light orange solid, m.p. 174.4–176.8 °C; yield 87%, >20:1 *dr*; ^1^H-NMR (CDCl_3_) δ: 1.54 (s, 3H), 1.61 (s, 3H), 2.42 (s, 3H), 2.90–2.93 (m, 1H), 2.94 (s, 3H), 3.30–3.34 (m, 1H), 3.62 (d, *J* = 6.0 Hz, 1H), 3.80 (d, *J* = 7.2 Hz, 1H), 3.84 (d, *J* = 6.0 Hz, 1H), 5.26–5.30 (m, 1H), 5.48 (s, 1H), 6.61 (d, *J* = 6.0 Hz, 1H), 6.96–7.03 (m, 3H), 7.18–7.21 (m, 1H), 7.55–7.56 (m, 2H); ^13^C-NMR (CDCl_3_) *δ*: 19.3, 20.4, 26.5, 27.2, 29.6, 35.7, 49.4, 61.5, 66.4, 67.6, 75.4, 107.2, 119.1, 121.4, 121.9, 123.0, 126.3, 128.4, 128.9, 133.9, 141.4, 142.6, 156.0, 174.7, 195.1; HRMS (ESI-TOF) *m*/*z*: Calcd. for C_28_H_29_N_3_NaO_3_S [M + Na]^+^: 510.1827; Found: 510.1829.

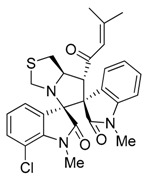



**4ea**: Light orange solid, m.p. 196.8–198.7 °C; yield 71%, >20:1 *dr*; ^1^H-NMR (CDCl_3_) δ: 1.55 (s, 3H), 1.59 (s, 3H), 2.90–2.94 (m, 1H), 2.96 (s, 3H), 3.23 (s, 3H), 3.32–3.35 (m, 1H), 3.57 (d, *J* = 6.4 Hz, 1H), 3.77 (d, *J* = 7.2 Hz, 1H), 3.87 (d, *J* = 6.4 Hz, 1H), 5.23–5.27 (m, 1H), 5.48 (s, 1H), 6.64 (d, *J* = 6.4 Hz, 1H), 6.99–7.04 (m, 2H), 7.20–7.24 (m, 2H), 7.55–7.57 (m, 1H), 7.62–7.64 (m, 1H); ^13^C-NMR (CDCl_3_) δ: 20.4, 26.5, 27.1, 29.6, 35.8, 49.6, 61.6, 66.4, 67.6, 75.6, 107.4, 115.1, 122.0, 122.2, 122.8, 128.4, 129.2, 132.3, 139.4, 142.4, 156.4, 174.1, 174.2, 194.6; HRMS (ESI-TOF) *m*/*z*: Calcd. for C_27_H_26_ClN_3_NaO_3_S [M + Na]^+^: 530.1281; Found: 530.1280.

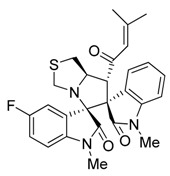



**4fa**: Light orange solid, m.p. 182.3–184.5 °C; yield 74%, >20:1 *dr*; ^1^H-NMR (CDCl_3_, 500 MHz) *δ*: 1.57–1.58 (m, 6H), 2.88 (s, 3H), 2.88–2.95 (m, 1H), 2.97 (s, 3H), 3.35–3.38 (m, 1H), 3.59 (d, *J* = 8.5 Hz, 1H), 3.77 (d, *J* = 9.0 Hz, 1H), 3.91 (d, *J* = 8.5 Hz, 1H), 5.24–5.28 (m, 1H), 5.52 (s, 1H), 6.58–6.64 (m, 2H), 7.01–7.05 (m, 2H), 7.20–7.23 (m, 1H), 7.54–7.56 (m, 1H), 7.62 (d, *J* = 7.5 Hz, 1H); ^13^C-NMR (CDCl_3_, 125 MHz) δ: 20.3, 26.4, 26.5, 27.1, 36.1, 50.4, 62.0, 65.8, 67.6, 76.4, 107.4, 108.0, 116.1 (d, *J_CF_* = 23.8 Hz), 117.1 (d, *J_CF_* = 26.3 Hz), 122.0, 122.7, 124.4, 128.5, 129.1, 139.4, 142.4, 156.4, 158.2 (d, *J_CF_* = 240.0 Hz), 173.6, 173.9, 194.5; HRMS (ESI-TOF) *m*/*z*: Calcd. for C_27_H_26_FN_3_NaO_3_S [M + Na]^+^: 514.1577; Found: 514.1580.

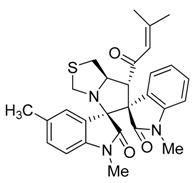



**4ma**: Light orange solid, m.p. 198.3–199.7 °C; yield 79%, >20:1 *dr*; ^1^H-NMR (CDCl_3_) δ: 1.55 (s, 3H), 1.60 (s, 3H), 2.40 (s, 3H), 2.85 (s, 3H), 2.94 (s, 3H), 2.95–2.97 (m, 1H), 3.33–3.37 (m, 1H), 3.63 (d, *J* = 6.4 Hz, 1H), 3.82 (d, *J* = 7.2 Hz, 1H), 3.87 (d, *J* = 6.4 Hz, 1H), 5.27–5.31 (m, 1H), 5.52 (s, 1H), 6.55 (d, *J* = 6.0 Hz, 1H), 6.60 (d, *J* = 6.4 Hz, 1H), 6.98–7.02 (m, 1H), 7.08–7.10 (m, 1H), 7.17–7.21 (m, 1H), 7.58–7.60 (m, 1H); ^13^C-NMR (CDCl_3_) δ: 20.3, 21.2, 26.1, 26.4, 27.1, 35.9, 50.0, 61.7, 66.0, 67.6, 76.3, 107.2, 107.4, 121.9, 122.9, 123.1, 128.4, 128.9, 129.3, 130.1, 130.9, 141.2, 142.5, 156.0, 173.7, 174.5, 195.0; HRMS (ESI-TOF) *m*/*z*: Calcd. for C_28_H_29_N_3_NaO_3_S [M + Na]^+^: 510.1827; Found: 510.1827.

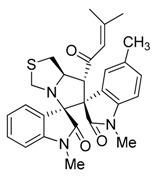



**4ag**: Light orange solid, m.p. 211.3–211.8 °C; yield 70%, >20:1 *dr*; ^1^H-NMR (CDCl_3_) δ: 1.48 (s, 3H), 1.49 (s, 3H), 2.27 (s, 3H), 2.83 (s, 3H), 2.84 (s, 3H), 2.87–2.91 (m, 1H), 3.29–3.33 (m, 1H), 3.57 (d, *J* = 7.2 Hz, 1H), 3.74 (d, *J* = 7.2 Hz, 1H), 3.86 (d, *J* = 6.4 Hz, 1H), 5.19–5.23 (m, 1H), 5.44 (s, 1H), 6.43 (d, *J* = 6.4 Hz, 1H), 6.61 (d, *J* = 6.4 Hz, 1H), 6.92 (d, *J* = 6.4 Hz, 1H), 7.02–7.05 (m, 1H), 7.23–7.26 (m, 1H), 7.38 (s, 1H), 7.67 (d, *J* = 6.0 Hz, 1H); ^13^C-NMR (CDCl_3_) δ: 20.2, 21.2, 26.2, 26.5, 27.1, 36.2, 50.7, 62.1, 65.8, 67.6, 76.5, 107.0, 107.8, 121.4, 122.9, 123.2, 128.9, 129.0, 129.3, 129.9, 131.3, 140.1, 143.4, 155.7, 173.8, 174.0, 195.0; HRMS (ESI-TOF) *m*/*z*: Calcd. for C_28_H_29_N_3_NaO_3_S [M + Na]^+^: 510.1827; Found: 510.1827.

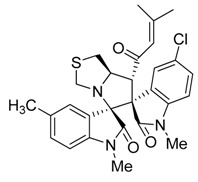



**4mh**: Light orange solid, m.p. 203.8–205.4 °C; yield 93%, >20:1 *dr*; ^1^H-NMR (CDCl_3_) δ: 1.52 (s, 3H), 1.61 (s, 3H), 2.33 (s, 3H), 2.82 (s, 3H), 2.87 (s, 3H), 2.88–2.91 (m, 1H), 3.27–3.30 (m, 1H), 3.55 (d, *J* = 6.4 Hz, 1H), 3.74 (d, *J* = 6.8 Hz, 1H), 3.80 (d, *J* = 6.4 Hz, 1H), 5.14–5.19 (m, 1H), 5.47 (s, 1H), 6.46–6.50 (m, 2H), 7.03–7.05 (m, 1H), 7.09–7.11 (m, 1H), 7.42 (s, 1H), 7.53 (s, 1H); ^13^C-NMR (CDCl_3_) δ: 20.5, 21.2, 26.2, 26.5, 27.2, 35.9, 50.0, 61.7, 65.8, 67.5, 76.0, 107.5, 108.1, 122.7, 127.4, 128.6, 128.8, 129.3, 130.3, 131.0, 141.1, 141.2, 156.7, 173.3, 174.0, 194.5; HRMS (ESI-TOF) *m*/*z*: Calcd. for C_28_H_28_ClN_3_NaO_3_S [M + Na]^+^: 544.1438; Found: 544.1435.

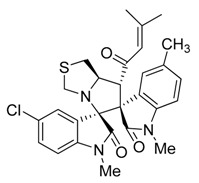



**4ng**: Light orange solid, m.p. 214.2–215.6 °C; yield 72%, >20:1 *dr*; ^1^H-NMR (CDCl_3_) δ: 1.49 (s, 3H), 2.27 (s, 3H), 2.82 (s, 3H), 2.87 (s, 3H), 2.88–2.91 (m, 1H), 3.29–3.32 (m, 1H), 3.50 (d, *J* = 7.2 Hz, 1H), 3.68 (d, *J* = 7.2 Hz, 1H), 3.85 (d, *J* = 7.2 Hz, 1H), 5.15–5.20 (m, 1H), 5.45 (s, 1H), 6.44 (d, *J* = 6.0 Hz, 1H), 6.53 (d, *J* = 7.2 Hz, 1H), 6.93–6.94 (m, 1H), 7.20–7.23 (m, 1H), 7.36 (s, 1H), 7.70 (s, 1H); ^13^C-NMR (CDCl_3_) δ: 20.3, 21.2, 26.3, 26.5, 27.1, 36.3, 50.9, 62.0, 65.7, 67.7, 76.4, 107.1, 108.5, 122.8, 124.4, 129.1, 129.4, 129.5, 129.8, 131.4, 140.1, 141.9, 156.0, 173.5, 173.7, 194.7; HRMS (ESI-TOF) *m*/*z*: Calcd. for C_28_H_28_ClN_3_NaO_3_S [M + Na]^+^: 544.1438; Found: 544.1439.

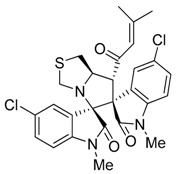



**4nh**: Light orange solid, m.p. 189.7–190.8 °C; yield 90%, >20:1 *dr*; ^1^H-NMR (CDCl_3_) δ: 1.54 (s, 3H), 1.59 (s, 3H), 2.85 (s, 3H), 2.88–2.91 (m, 4H), 3.29–3.32 (m, 1H), 3.49 (d, *J* = 7.2 Hz, 1H), 3.70 (d, *J* = 7.2 Hz, 1H), 3.84 (d, *J* = 7.2 Hz, 1H), 5.10–5.15 (m, 1H), 5.49 (s, 1H), 6.49 (d, *J* = 6.4 Hz, 1H), 6.55 (d, *J* = 6.8 Hz, 1H), 7.12–7.14 (m, 1H), 7.22–7.24 (m, 1H), 7.56 (s, 1H), 7.66 (s, 1H); ^13^C-NMR (CDCl_3_) δ: 20.5, 26.4, 26.6, 27.2, 36.3, 50.7, 62.0, 65.5, 67.5, 76.1, 108.2, 108.7, 122.6, 124.4, 127.2, 128.8, 129.1, 129.3, 130.0, 141.0, 141.9, 157.0, 173.0, 173.4, 194.0; HRMS (ESI-TOF) *m*/*z*: Calcd. for C_27_H_25_Cl_2_N_3_NaO_3_S [M + Na]^+^: 564.0891; Found: 564.0890.

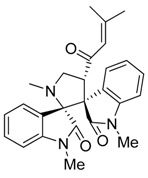



**5aa**: Light orange solid, m.p. 201.1–202.9 °C; yield 82%, >20:1 *dr*; ^1^H-NMR (CDCl_3_) δ: 1.49 (s, 3H), 1.80 (s, 3H), 2.19 (s, 3H), 2.84 (s, 3H), 2.98 (s, 3H), 3.38–3.42 (m, 1H), 4.01–4.05 (m, 1H), 4.51–4.54 (m, 1H), 5.39 (s, 1H), 6.55 (d, *J* = 6.4 Hz, 2H), 6.87–6.91 (m, 1H), 7.01–7.04 (m, 1H), 7.11–7.14 (m, 1H), 7.21–7.24 (m, 1H), 7.28–7.31 (m, 1H), 7.47 (d, *J* = 6.4 Hz, 1H); ^13^C-NMR (CDCl_3_) δ: 20.5, 25.3, 26.3, 27.1, 35.3, 52.9, 55.8, 61.8, 78.6, 107.1, 107.5, 121.7, 122.1, 123.9, 124.8, 126.2, 127.8, 128.6, 129.5, 143.2, 144.1, 155.3, 173.8, 176.9, 195.6; HRMS (ESI-TOF) *m*/*z*: Calcd. for C_26_H_27_N_3_NaO_3_ [M + Na]^+^: 452.1950; Found: 452.1953.

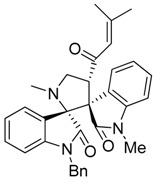



**5ba**: Light orange solid, m.p. 203.7–204.9 °C; yield 87%, >20:1 *dr*; ^1^H-NMR (CDCl_3_) δ: 1.48 (s, 3H), 1.81 (s, 3H), 2.23 (s, 3H), 2.99 (s, 3H), 3.40–3.44 (m, 1H), 4.06–4.10 (m, 1H), 4.36 (d, *J* = 12.8 Hz, 1H), 4.53–4.57 (m, 1H), 4.84 (d, *J* = 12.4 Hz, 1H), 5.38 (s, 1H), 6.38 (d, *J* = 6.0 Hz, 1H), 6.61 (d, *J* = 6.4 Hz, 1H), 6.74 (d, *J* = 5.2 Hz, 2H), 6.84–6.87 (m, 1H), 6.98–7.01 (m, 1H), 7.07–7.20 (m, 5H), 7.33–7.35 (m, 1H), 7.51–7.53 (m, 1H); ^13^C-NMR (CDCl_3_) δ: 20.5, 26.3, 27.1, 35.3, 42.9, 53.0, 55.9, 61.8, 78.4, 107.2, 108.7, 122.2, 123.8, 126.7, 127.1, 128.3, 128.4, 128.6, 143.4, 155.3, 173.7, 177.1, 195.7; HRMS (ESI-TOF) *m*/*z*: Calcd. for C_32_H_31_N_3_NaO_3_ [M + Na]^+^: 528.2263; Found: 528.2265.

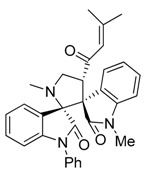



**5ca**: Light orange solid, m.p. 191.4–193.2 °C; yield 72%, >20:1 *dr*; ^1^H-NMR (CDCl_3_) δ: 1.49 (s, 3H), 1.86 (s, 3H), 2.36 (s, 3H), 3.00 (s, 3 H), 3.46–3.50 (m, 1H), 4.03–4.06 (m, 1H), 4.57–4.61 (m, 1H), 5.41 (s, 1H), 6.41 (d, *J* = 6.0 Hz, 1H), 6.59 (d, *J* = 6.4 Hz, 1H), 6.83–6.89 (m, 3H), 7.02–7.05 (m, 1H), 7.09–7.13 (m, 1H), 7.15–7.21 (m, 2H), 7.28–7.33 (m, 1H), 7.36–7.39 (m, 2H), 7.53–7.54 (m, 1H); ^13^C-NMR (CDCl_3_) δ: 20.6, 26.3, 27.2, 35.3, 53.4, 55.4, 62.3, 78.8, 107.3, 108.7, 124.0, 126.3, 126.7, 128.0, 128.7, 129.3, 129.5, 143.5, 144.4, 155.3, 173.4, 177.5, 195.6; HRMS (ESI-TOF) *m*/*z*: Calcd. for C_31_H_29_N_3_NaO_3_ [M + Na]^+^: 514.2107; Found: 514.2109.

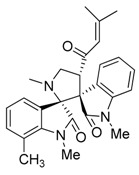



**5da**: Light orange solid, m.p. 145.1–147.8 °C; yield 86%, >20:1 *dr*; ^1^H-NMR (CDCl_3_) δ: 1.49 (s, 3H), 1.82 (s, 3H), 2.19 (s, 3H), 2.34 (s, 3H), 2.97 (s, 3H), 3.09 (s, 3H), 3.37–3.42 (m, 1H), 3.98–4.03 (m, 1H), 4.49–4.54 (m, 1H), 5.37 (s, 1H), 6.56 (d, *J* = 8.0 Hz, 1H), 6.86–6.94 (m, 3H), 7.12–7.15 (m, 1H), 7.25–7.32 (m, 2H); ^13^C-NMR (CDCl_3_) δ: 19.0, 20.5, 26.3, 27.1, 28.7, 35.3, 52.9, 55.6, 62.0, 77.9, 107.1, 118.8, 121.6, 121.8, 123.9, 124.0, 127.7, 128.5, 133.3, 141.8, 143.2, 155.2, 174.5, 177.0, 195.7; HRMS (ESI-TOF) *m*/*z*: Calcd. for C_27_H_29_N_3_NaO_3_ [M + Na]^+^: 466.2107; Found: 466.2105.

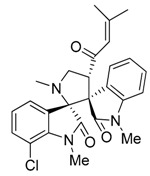



**5ea**: Light orange solid, m.p. 181.3–183.7 °C; yield 83%, >20:1 *dr*; ^1^H-NMR (CDCl_3_) δ: 1.49 (s, 3H), 1.82 (s, 3H), 2.19 (s, 3H), 2.98 (s, 3H), 3.19 (s, 3H), 3.38–3.42 (m, 1H), 3.97–4.02 (m, 1H), 4.49–4.53 (m, 1H), 5.35 (s, 1H), 6.59 (d, *J* = 7.6 Hz, 1H), 6.88–6.95 (m, 2H), 7.13–7.18 (m, 2H), 7.23 (d, *J* = 7.6 Hz, 1H), 7.38 (d, *J* = 7.6 Hz, 1H); ^13^C-NMR (CDCl_3_) δ: 20.6, 26.3, 27.2, 28.7, 35.2, 53.0, 55.6, 62.1, 78.1, 107.3, 114.8, 121.9, 122.7, 123.8, 124.8, 128.8, 131.9, 139.9, 143.1, 155.6, 174.1, 176.8, 195.4; HRMS (ESI-TOF) *m*/*z*: Calcd. for C_26_H_26_ClN_3_NaO_3_ [M + Na]^+^: 486.1560; Found: 486.1560.

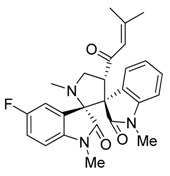



**5fa**: Light orange solid, m.p. 187.7–189.3 °C; yield 71%, >20:1 *dr*; ^1^H-NMR (CDCl_3_) δ: 1.50 (s, 3H), 1.81 (s, 3H), 2.20 (s, 3H), 2.84 (s, 3H), 3.02 (s, 3H), 3.36–3.40 (m, 1H), 3.98–4.01 (m, 1H), 4.49–4.53 (m, 1H), 5.38 (s, 1H), 6.47–6.49 (m, 1H), 6.58 (d, *J* = 7.5 Hz, 1H), 6.88–6.95 (m, 2H), 7.13–7.16 (m, 1H), 7.27–7.30 (m, 2H); ^13^C-NMR (CDCl_3_) δ: 20.6, 25.5, 26.4, 27.2, 35.3, 52.9, 55.8, 61.8, 78.6, 107.3, 107.9, 108.0, 114.4 (d, *J_CF_* = 26.3 Hz), 115.8 (d, *J_CF_* = 23.8 Hz), 121.9, 123.8, 124.5, 125.9, 127.9, 128.9, 140.1, 143.2, 155.6, 158.8 (d, *J_CF_* = 238.8 Hz), 173.6, 176.7, 195.4; HRMS (ESI-TOF) *m*/*z*: Calcd. for C_26_H_26_FN_3_NaO_3_ [M + Na]^+^: 470.1856; Found: 470.1859.

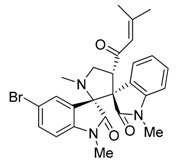



**5ga**: Light orange solid, m.p. 203.3–205.8 °C; yield 81%, >20:1 *dr*; ^1^H-NMR (CDCl_3_) δ: 1.48 (s, 3H), 1.64 (s, 3H), 2.07–2.11 (m, 2H), 2.23–2.29 (m, 2H), 2.48-2.53 (m, 1H), 2.72–2.75 (m, 1H), 2.93 (s, 3H), 3.18 (s, 3H), 4.58–4.63 (m, 2H), 5.77 (s, 1H), 6.49–6.51 (m, 2H), 6.62 (d, *J* = 6.4 Hz, 1H), 7.16–7.21 (m, 2H), 7.27–7.31 (m, 1H), 7.76 (d, *J* = 6.0 Hz, 1H); ^13^C-NMR (CDCl_3_) δ: 20.0, 26.1, 26.2, 27.1, 30.8, 31.3, 47.3, 58.9, 65.2, 67.2, 107.7, 108.7, 114.3, 122.4, 122.9, 127.2, 128.8, 129.2, 131.9, 142.7, 143.2, 154.6, 172.6, 176.5, 196.6; HRMS (ESI-TOF) *m/z*: Calcd. for C_26_H_26_BrN_3_NaO_3_ [M + Na]^+^: 530.1055; Found: 530.1057.

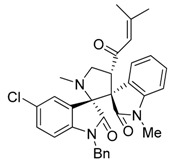



**5ia**: Light orange solid, m.p. 174.7–175.9 °C; yield 71%, >20:1 *dr*; ^1^H-NMR (CDCl_3_) δ: 1.50 (s, 3H), 1.81 (s, 3H), 2.24 (s, 3H), 3.04 (s, 3H), 3.39–3.44 (m, 1H), 4.04–4.08 (m, 1H), 4.36 (d, *J* = 16.0 Hz, 1H), 4.50–4.55 (m, 1H), 4.82 (d, *J* = 16.0 Hz, 1H), 5.38 (s, 1H), 6.30 (d, *J* = 8.4 Hz, 1H), 6.64 (d, *J* = 8.0 Hz, 1H), 6.71 (d, *J* = 6.4 Hz, 2H), 6.85–6.89 (m, 1H), 7.05–7.07 (m, 1H), 7.11–7.22 (m, 4H), 7.31 (d, *J* = 7.2 Hz, 1H), 7.53 (s, 1H); ^13^C-NMR (CDCl_3_) δ: 20.6, 26.4, 27.1, 35.3, 43.1, 53.0, 55.8, 61.8, 78.3, 107.3, 109.6, 122.2, 123.8, 126.7, 126.9, 127.3, 128.6, 128.8, 129.4, 141.8, 143.4, 155.7, 173.2, 176.8, 195.4; HRMS (ESI-TOF) *m*/*z*: Calcd. for C_32_H_30_ClN_3_NaO_3_ [M + Na]^+^: 562.1873; Found: 562.1875.

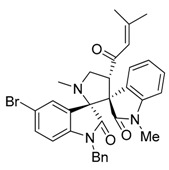



**5ja**: Light orange solid, m.p. 151.3–153.9 °C; yield 88%, >20:1 *dr*; ^1^H-NMR (CDCl_3_) δ: 1.50 (s, 3H), 1.82 (s, 3H), 2.24 (s, 3H), 3.04 (s, 3H), 3.40–3.43 (m, 1H), 4.03–4.07 (m, 1H), 4.36 (d, *J* = 12.8 Hz, 1H), 4.50–4.54 (m, 1H), 4.81 (d, *J* = 12.8 Hz, 1H), 5.38 (s, 1H), 6.24 (d, *J* = 6.8 Hz, 1H), 6.64 (d, *J* = 6.4 Hz, 1H), 6.71 (d, *J* = 5.6 Hz, 2H), 6.85–6.88 (m, 1H), 7.12–7.22 (m, 5H), 7.28–7.31 (m, 1H), 7.66 (s, 1H); ^13^C-NMR (CDCl_3_) δ: 20.6, 26.4, 27.2, 35.4, 43.1, 53.1, 55.7, 61.9, 78.4, 107.4, 110.2, 115.2, 122.3, 123.9, 124.7, 126.4, 126.7, 127.4, 128.3, 128.6, 128.8, 129.6, 132.4, 134.9, 142.4, 143.4, 155.7, 173.1, 176.9, 195.4; HRMS (ESI-TOF) *m*/*z*: Calcd. for C_32_H_30_BrN_3_NaO_3_ [M + Na]^+^: 606.1368; Found: 606.1369.

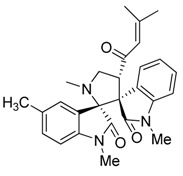



**5ma**: Light orange solid, m.p. 188.2–189.6 °C; yield 89%, >20:1 *dr*; ^1^H-NMR (CDCl_3_) δ: 1.50 (s, 3H), 1.81 (s, 3H), 2.19 (s, 3H), 2.34 (s, 3H), 2.82 (s, 3H), 2.98 (s, 3H), 3.37–3.41 (m, 1H), 4.01–4.04 (m, 1H), 4.49–4.53 (m, 1H), 5.40 (s, 1H), 6.43 (d, *J* = 6.4 Hz, 1H), 6.55 (d, *J* = 6.0 Hz, 1H), 6.86–6.90 (m, 1H), 7.01–7.02 (m, 1H), 7.11–7.14 (m, 1H), 7.28–7.30 (m, 2H); ^13^C-NMR (CDCl_3_) δ: 20.5, 21.0, 25.3, 26.2, 27.1, 35.3, 52.9, 55.6, 61.8, 78.6, 107.1, 107.2, 121.7, 123.9, 126.8, 127.8, 128.5, 129.7, 131.5, 141.6, 143.1, 155.2, 173.6, 177.0, 195.6; HRMS (ESI-TOF) *m*/*z*: Calcd. for C_27_H_29_N_3_NaO_3_ [M + Na]^+^: 466.2107; Found: 466.2108.

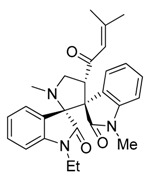



**5na**: Light orange solid, m.p. 170.2–171.7 °C; yield 93%, >20:1 *dr*; ^1^H-NMR (CDCl_3_) δ: 0.83–0.86 (m, 3H), 1.49 (s, 3H), 1.83 (s, 3H), 2.19 (s, 3H), 2.98 (s, 3H), 3.23–3.30 (m, 1H), 3.38–3.42 (m, 1H), 3.54–3.61 (m, 1H), 4.01–4.05 (m, 1H), 4.52–4.55 (m, 1H), 5.38 (s, 1H), 6.55–6.58 (m, 2H), 6.86–6.89 (m, 1H), 6.99–7.03 (m, 1H), 7.11–7.14 (m, 1H), 7.19–7.23 (m, 1H), 7.29–7.30 (m, 1H), 7.48–7.50 (m, 1H); ^13^C-NMR (CDCl_3_) δ: 12.2, 20.5, 26.2, 27.1, 33.7, 35.2, 53.0, 55.6, 61.8, 78.2, 107.1, 107.5, 121.7, 121.8, 123.9, 124.7, 126.4, 127.9, 128.5, 129.4, 143.1, 143.2, 155.2, 173.3, 177.2, 195.7; HRMS (ESI-TOF) *m*/*z*: Calcd. for C_27_H_29_N_3_NaO_3_ [M + Na]^+^: 466.2107; Found: 466.2107.

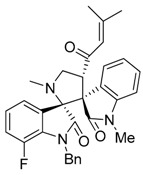



**5oa**: Light orange solid, m.p. 181.1–182.5 °C; yield 77%, >20:1 dr; ^1^H-NMR (CDCl_3_, 500 MHz) δ: 1.49 (s, 3H), 1.75 (s, 3H), 2.21 (s, 3H), 2.98 (s, 3H), 3.37–3.41 (m, 1H), 4.05–4.09 (m, 1H), 4.49–4.53 (m, 1H), 4.67 (d, *J* = 15.5 Hz, 1H), 4.80 (d, *J* = 15.5 Hz, 1H), 5.37 (s, 1H), 6.62 (d, *J* = 8.0 Hz, 1H), 6.85–6.99 (m, 5H), 7.13–7.20 (m, 4H), 7.33 (d, *J* = 7.5 Hz, 2H); ^13^C-NMR (CDCl_3_, 125 MHz) δ: 20.5, 26.4, 27.1, 35.3, 44.7, 52.7, 56.0, 61.9, 78.4, 107.2, 117.7 (d, *J_CF_* = 18.8 Hz), 122.2, 122.6, 123.6, 124.7, 127.1, 128.3 (d, *J_CF_* = 16.3 Hz), 128.8, 136.8, 143.2, 146.8 (d, *J_CF_* = 242.5 Hz), 155.6, 173.3, 176.5, 195.4; HRMS (ESI-TOF) *m*/*z*: Calcd. for C_32_H_30_FN_3_NaO_3_ [M + Na]^+^: 546.2169; Found: 546.2172.

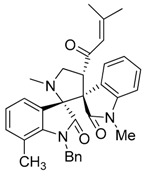



**5pa**: Light orange solid, m.p. 131.3–132.8 °C; yield 77%, >20:1 *dr*; ^1^H-NMR (CDCl_3_) δ: 1.48 (s, 3H), 1.78 (s, 3H), 2.02 (s, 3H), 2.25 (s, 3H), 3.03 (s, 3H), 3.40–3.44 (m, 1H), 4.06–4.10 (m, 1H), 4.51–4.54 (m, 1H), 4.70 (d, *J* = 13.6 Hz, 1H), 5.03 (d, *J* = 13.6 Hz, 1H), 5.38 (s, 1H), 6.62–6.66 (m, 3H), 6.79–6.82 (m, 1H), 6.88–6.95 (m, 2H), 7.11–7.19 (m, 4H), 7.27 (d, *J* = 6.0 Hz, 1H), 7.43 (d, *J* = 6.0 Hz, 1H); ^13^C-NMR (CDCl_3_) δ: 18.6, 20.5, 26.3, 27.1, 35.3, 44.2, 52.9, 55.9, 61.9, 77.6, 107.1, 118.9, 122.1, 122.2, 123.8, 124.5, 125.3, 126.6, 128.4, 128.5, 128.6, 133.6, 137.4, 155.2, 174.5, 177.2, 195.7; HRMS (ESI-TOF) *m*/*z*: Calcd. for C_33_H_33_N_3_NaO_3_ [M + Na]^+^: 542.2420; Found: 542.2423.

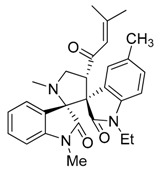



**5ai**: Light orange solid, m.p. 138.0–139.8 °C; yield 87%, 19:1 *dr*; ^1^H-NMR (CDCl_3_) δ: 0.81–0.83 (m, 3H), 1.38 (s, 3H), 1.74 (s, 3H), 2.14 (s, 3H), 2.16 (s, 3H), 2.74 (s, 3H), 3.27–3.34 (m, 2H), 3.61–3.66 (m, 1H), 3.91–3.95 (m, 1H), 4.45–4.49 (m, 1H), 5.27 (s, 1H), 6.37 (d, *J* = 6.4 Hz, 1H), 6.45 (d, *J* = 6.4 Hz, 1H), 6.83 (d, *J* = 6.4 Hz, 1H), 6.91–6.95 (m, 1H), 7.04 (s, 1H), 7.11–7.14 (m, 1H), 7.41–7.43 (m, 1H); ^13^C-NMR (CDCl_3_) δ: 12.2, 20.5, 20.9, 25.1, 27.1, 34.5, 35.4, 53.2, 55.9, 61.7, 78.7, 106.9, 107.3, 122.1, 134.0, 125.2, 126.6, 128.6, 128.8, 129.4, 130.8, 140.0, 144.2, 154.8, 174.1, 176.6, 195.9; HRMS (ESI-TOF) *m*/*z*: Calcd. for C_28_H_31_N_3_NaO_3_ [M + Na]^+^: 480.2263; Found: 480.2265.

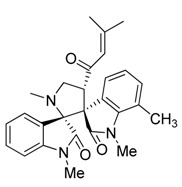



**5aj**: Light orange solid, m.p. 214.5–215.1 °C; yield 75%, >20:1 *dr*; ^1^H-NMR (CDCl_3_) δ: 1.47 (s, 3H), 1.71 (s, 3H), 2.11 (s, 3H), 2.30 (s, 3H), 2.77 (s, 3H), 3.18 (s, 3H), 3.28–3.32 (m, 1H), 3.96–3.99 (m, 1H), 4.41–4.44 (m, 1H), 5.36 (s, 1H), 6.52 (d, *J* = 6.4 Hz, 1H), 6.69–6.72 (m, 1H), 6.78 (d, *J* = 5.6 Hz, 1H), 6.96–6.99 (m, 1H), 7.11 (d, *J* = 5.6 Hz, 1H), 7.16–7.21 (m, 1H), 7.38–7.40 (m, 1H); ^13^C-NMR (CDCl_3_) δ: 19.1, 20.5, 25.5, 27.2, 29.8, 35.4, 52.7, 56.1, 61.4, 78.8, 107.6, 118.3, 121.5, 122.0, 123.9, 125.9, 126.3, 129.4, 132.4, 140.9, 144.0, 155.0, 173.7, 177.4, 195.7; HRMS (ESI-TOF) *m*/*z*: Calcd. for C_27_H_29_N_3_NaO_3_ [M + Na]^+^: 466.2107; Found: 466.2109.

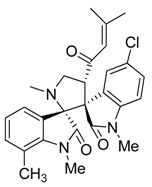



**5dh**: Light orange solid, m.p. 185.8–187.2 °C; yield 93%, 12:1 *dr*; ^1^H-NMR (CDCl_3_) δ: 1.47 (s, 3H), 1.82 (s, 3H), 2.11 (s, 3H), 2.29 (s, 3H), 2.89 (s, 3H), 3.07 (s, 3H), 3.32–3.36 (m, 1H), 3.87–3.91 (m, 1H), 4.40–4.43 (m, 1H), 5.35 (s, 1H), 6.41 (d, *J* = 7.2 Hz, 1H), 6.81–6.84 (m, 1H), 6.87 (d, *J* = 6.4 Hz, 1H), 7.04–7.06 (m, 1H), 7.20–7.22 (m, 2H); ^13^C-NMR (CDCl_3_) δ: 19.0, 20.6, 26.4, 27.2, 28.8, 35.2, 52.9, 55.6, 61.9, 77.8, 108.0, 119.0, 122.0, 123.8, 124.0, 127.1, 128.0, 128.5, 133.5, 141.8, 142.0, 156.0, 174.4, 176.7, 195.3; HRMS (ESI-TOF) *m*/*z*: Calcd. for C_27_H_28_ClN_3_NaO_3_ [M + Na]^+^: 500.1717; Found: 500.1719.

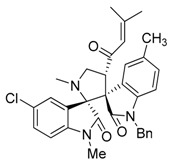



**5nb**: Light orange solid, m.p. 200.2–201.8 °C; yield 79%, 20:1 *dr*; ^1^H-NMR (CDCl_3_) δ: 1.34 (s, 3H), 1.72 (s, 3H), 2.12 (s, 3H), 2.16 (s, 3H), 2.75 (s, 3H), 3.32–3.35 (m, 1H), 3.95–3.99 (m, 1H), 4.41 (d, *J* = 12.0 Hz, 1H), 4.45–4.49 (m, 1H), 4.95 (d, *J* = 12.4 Hz, 1H), 5.24 (d, *J* = 14.0 Hz, 1H), 6.24 (d, *J* = 6.4 Hz, 1H), 6.43 (d, *J* = 6.4 Hz, 1H), 6.72 (d, *J* = 6.4 Hz, 1H), 6.80 (d, *J* = 2.8 Hz, 2H), 7.03 (s, 1H), 7.11–7.13 (m, 3H), 7.15–7.17 (m, 1H), 7.49 (s, 1H); ^13^C-NMR (CDCl_3_) δ: 20.5, 21.0, 25.4, 27.1, 35.4, 43.8, 53.2, 56.5, 61.8, 78.5, 108.1, 108.4, 123.8, 126.3, 126.8, 127.4, 128.1, 128.5, 128.6, 129.0, 129.4, 131.4, 135.5, 140.3, 155.3, 173.7, 177.0, 195.5; HRMS (ESI-TOF) *m*/*z*: Calcd. for C_33_H_32_ClN_3_NaO_3_ [M + Na]^+^: 576.2030; Found: 576.2032.

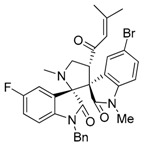



**5oc**: Light orange solid, m.p. 195.3–196.9 °C; yield 75%, >20:1 *dr*; ^1^H-NMR (CDCl_3_) δ: 1.47 (s, 3H), 1.84 (s, 3H), 2.15 (s, 3H), 2.95 (s, 3H), 3.34–3.37 (m, 1H), 3.91–3.94 (m, 1H), 4.29 (d, *J* = 12.4 Hz, 1H), 4.41–4.45 (m, 1H), 4.83 (d, *J* = 12.8 Hz, 1H), 5.34 (s, 1H), 6.26–6.29 (m, 1H), 6.45 (d, *J* = 6.4 Hz, 1H), 6.72–6.76 (m, 3H), 7.11–7.13 (m, 3H), 7.20–7.22 (m, 1H), 7.26–7.28 (m, 1H), 7.41 (s, 1H); ^13^C-NMR (CDCl_3_, 100 MHz) δ: 20.8, 26.5, 27.3, 35.3, 43.3, 53.0, 55.9, 61.6, 78.3, 108.8, 109.3, 109.4, 114.6 (d, *J_CF_* = 26.3 Hz), 115.1, 116.1 (d, *J_CF_* = 23.8 Hz), 123.6, 126.8, 126.9, 127.5, 128.7, 131.4, 131.7, 135.1, 142.6, 156.6, 158.0, 158.9 (d, *J_CF_* = 240.0 Hz), 173.2, 176.5, 195.0; HRMS (ESI-TOF) *m*/*z*: Calcd. for C_32_H_29_BrFN_3_NaO_3_ [M + Na]^+^: 624.1274; Found: 624.1275.

All the NMR spectra of compounds **3**–**5** see Supplementary Materials.

### 3.4. Cytotoxicity Assay

The human cancer cell lines, K562 was purchased from Chinese Academy of Sciences, Kunming Cell Bank. All the cells were cultured in RPMI-1640 medium (GIBICO, Sigma-Aldrich Company, St. Louis, MO, USA), supplemented with 10% fetal bovine serum (Hyclone, Sigma-Aldrich Company) and penicillin-streptomycin (100 U/mL, respectively) in 5% CO_2_ at 37 °C. The cytotoxicity assay was performed according to the MTT (3-(4,5-dimethylthiazol-2-yl)-2,5-diphenyltetrazolium bromide) method in 96-well microplates. Briefly, 5000 cells were seeded into each well of 96-well cell culture plates and allowed to grow for 24 h before drug addition. Each tumor cell line was exposed to the test compound at the concentrations of 6.25, 12.5, 25, 50, and 100 μmol·L^−1^ in triplicates for 48 h, comparable to Cisplatin (Aladdin, Shanghai, China). Then the MTT reagent was added to reaction with the cancer cells for 4 h. At least, measure the OD value at 490 wavelengths. IC_50_ of all the compounds were calculated by IBM SPSS Statistics (version 19).

## 4. Conclusions

In conclusion, we have developed a facile and efficient methodology for the synthesis of novel turmerone motif-fused 3,3′-pyrrolidinyl-dispirooxindoles **3**–**5** via a multicomponent 1,3-dipolar cycloaddition event, reacting dienones **2** with azomethine ylides (thermally generated in situ from isatins and proline or thioproline or sarcosine). Products bearing four or three consecutive stereocenters consist of two oxindole moieties and a pyrrolidinyl core, including vicinal spiroquaternary stereocenters. These structures were smoothly obtained in high yields (up to 93% yield) with good diastereoselectivity (up to >20:1). Another valuable feature of this method was its possible application in the design of new hybrid architectures for biological screenings through the adequate fusion of these sub-units of turmerone and 3,3′-pyrrolidinyl-dispirooxindole, generating drug-like molecules.

## Figures and Tables

**Table 1:**
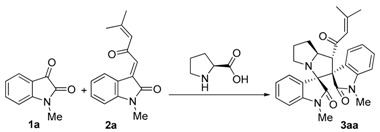
Optimization of reaction conditions ^a^.

Entry ^a^	Solvent	T (°C)	Time (h)	Yield ^b^ (%)	Dr ^c^
1	toluene	80	5	67	10:1
2	DCE	reflux	5	61	15:1
3	EtOAc	reflux	5	48	12:1
4	EtOH	reflux	5	83	18:1
5	THF	reflux	5	71	16:1
6	H_2_O	80	5	39	11:1
7	CH_3_CN	reflux	5	82	19:1
8	EtOH	40	48	52	17:1
9 ^d^	EtOH	reflux	3	89	20:1
10 ^e^	EtOH	reflux	5	57	16:1
11 ^f^	EtOH	reflux	3	72	19:1

^a^ Unless otherwise noted, reactions were carried out with 0.6 mmol of **1a**, 0.4 mmol of **2a**, 0.8 mmol of proline in 6.0 mL of solvent; ^b^ Isolated yield after flash chromatography; ^c^ Determined by ^1^H-NMR analysis of the crude products; ^d^ The reaction was carried out in 10.0 mL of EtOH; ^e^ The reaction was carried out in 3.0 mL of EtOH; ^f^ The reaction was carried out using 0.5 mmol of proline.

**Table 2:**
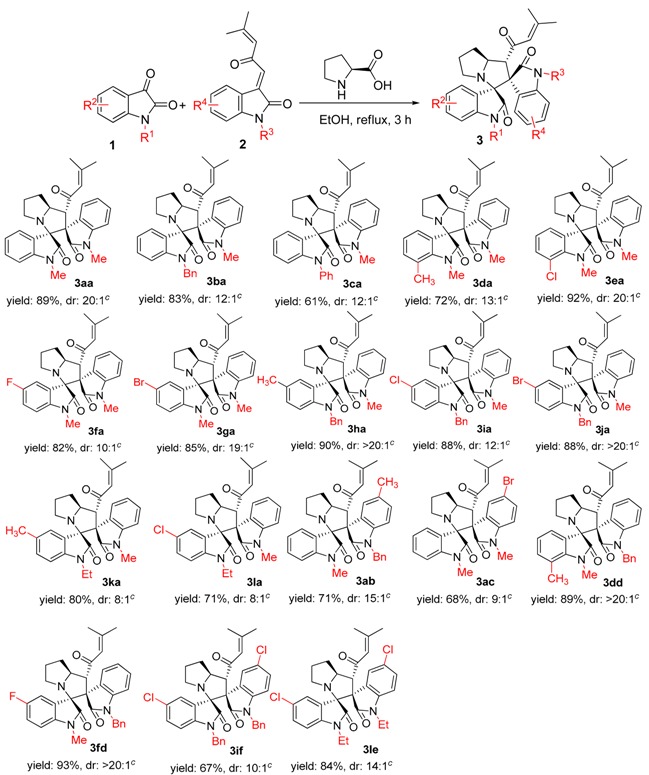
Synthesis of 3,3′-pyrrolidinyl-dispirooxindoles **3** ^a,b^.

^a^ Unless otherwise noted, reactions were carried out with 0.6 mmol of **1**, 0.4 mmol of **2**, 0.8 mmol of proline in 10.0 mL of EtOH at reflux for 3 h; ^b^ Isolated yield after flash chromatography; ^c^ Determined by ^1^H-NMR analysis of the crude products.

**Table 3:**
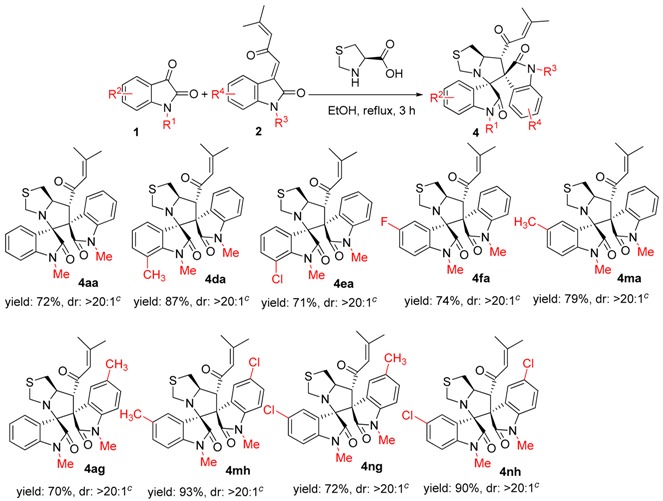
Synthesis of 3,3′-pyrrolidinyl-dispirooxindoles **4** ^a,b^.

^a^ Unless otherwise noted, reactions were carried out with 0.6 mmol of **1**, 0.4 mmol of **2**, 0.8 mmol of thioproline in 10.0 mL of EtOH at reflux for 3 h; ^b^ Isolated yield after flash chromatography; ^c^ Determined by ^1^H-NMR analysis of the crude products.

**Table 4:**
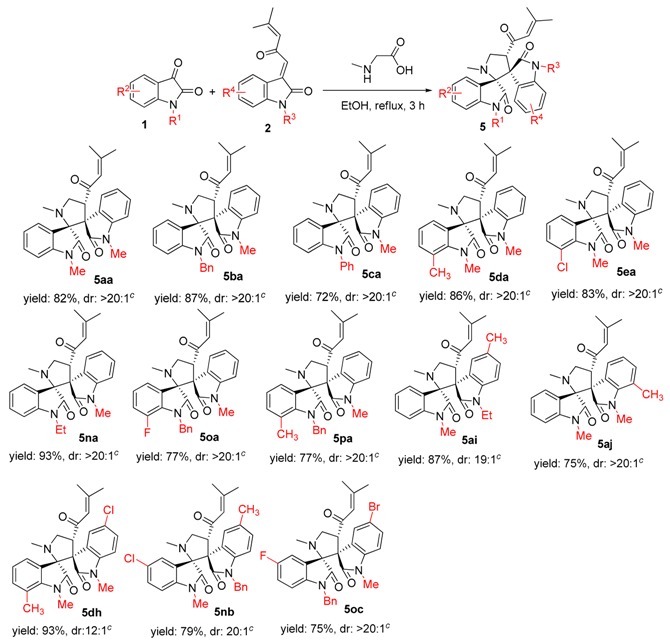
Synthesis of 3,3′-pyrrolidinyl-dispirooxindoles **5** ^a,b^.

^a^ Unless otherwise noted, reactions were carried out with 0.6 mmol of **1**, 0.4 mmol of **2a**, 0.8 mmol of sarcosine in the 10.0 mL of EtOH at reflux for 3 h; ^b^ Isolated yield after flash chromatography; ^c^ Determined by ^1^H-NMR analysis of the crude products.

**Table 5:** Cytotoxicity of the nine compounds **3le**, **3fa**, **3la**, **3ia**, **3fd**, **4da**, **5ia**, **5ba**, **5oa** and **5pa** on human leukemia cells K562 ^a^.

Compound	3le	3fa	3la	3ia	3fd	4da	5ia	5ba	5oa	5pa	Cisplatin
K562 IC_50_ (μM)	43.5	51.3	33.8	77.8	62.3	38.5	49.4	79.0	22.3	27.6	21.3

^a^ The IC_50_ concentration represents the concentration which results in a 50% decrease in cell growth after two days of incubation. The given values are mean values of three experiments.

## Supplementary Materials

Supplementary materials are available online.
