# Supplementary material for: Molecular Hybridization-Guided One-Pot Multicomponent Synthesis of Turmerone Motif-Fused 3,3′-Pyrrolidinyl-dispirooxindoles via a 1,3-Dipolar Cycloaddition Reaction
Source: Molecules. 2017 Apr 17;22(4):645. doi: 10.3390/molecules22040645 (PMC6154684; doi:10.3390/molecules22040645)

Molecular Hybridization-guided One-pot Multicomponent Synthesis of Turmerone Motif-fused 3,3'-Pyrrolidinyl-dispirooxindoles *via* a 1,3-Dipolar Cycloaddition Reaction

Bing Lin,[1,3] Gen Zhou,[1,3] Yi Gong,[1] Qi-Di Wei,[1] Min-Yi Tian,[a] Xiong-Li Liu, [1,*] Ting-Ting Feng,[1] Ying Zhou, [1,*] and Wei-Cheng Yuan[2]

[1] Guizhou Engineering Center for Innovative Traditional Chinese Medicine and Ethnic Medicine, College of Pharmacy, Guizhou University, Guiyang, 550025; zzhao@gzu.edu.cn

[2] Key Laboratory for Asymmetric Synthesis & Chirotechnology of Sichuan Province, Chengdu Institute of Organic Chemistry, Chinese Academy of Sciences, Chengdu 610041, China; yuanwc@cioc.ac.cn

[3] These two authors contributed equally to this work.

* Correspondence: *yzhou71@yeah.net* (Y.Z.); xlliu1@gzu.edu.cn (X.L.L.)

Supporting Information

Table of Contents

1. General Experimental Information.. S1

2. The Copies of ^{1}H NMR, ^{13}C NMR Spectra for Compounds **3-5**………..…………..…………S2

1. General Experimental Information

The ^{1}H and ^{13}C NMR spectra were recorded on Bruker Avance DMX 400 MHz NMR spectrometers in $CDCl_3$ using TMS as internal standard. Chemical shifts were reported as δ values (ppm). High-resolution mass spectra (HRMS-ESI) were obtained on a Micro^{TM} Q-TOF Mass Spectrometer. Melting points were uncorrected and recorded on an Electothermal 9100 digital melting point apparatus.

Reagents were purchased from commercial sources and were used as received unless mentioned otherwise. Reactions were monitored by thin layer chromatography using silica gel GF_{254} plates. Column chromatography was performed on silica gel (300-400 mesh).

2. The Copies of ^{1}H NMR, ^{13}C NMR Spectra for Compounds 3-5.

^{1}H and ^{13}C NMR of 3aa

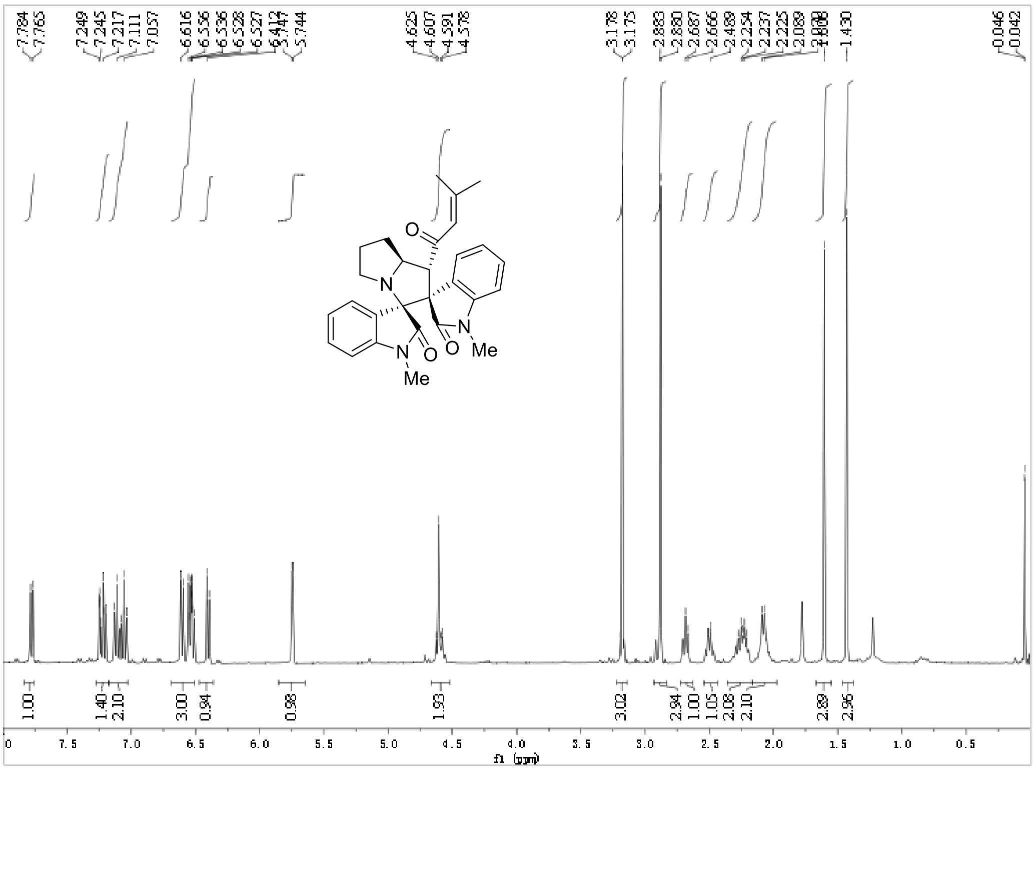

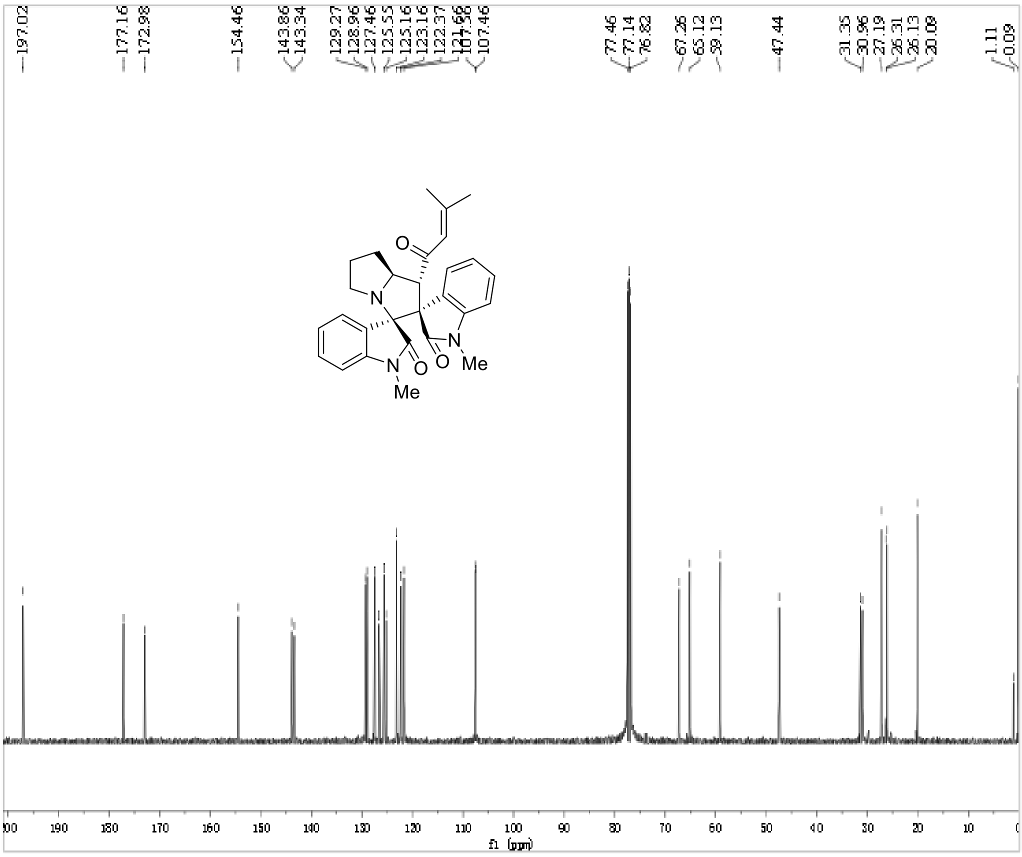

^{1}H and ^{13}C NMR of 3ba

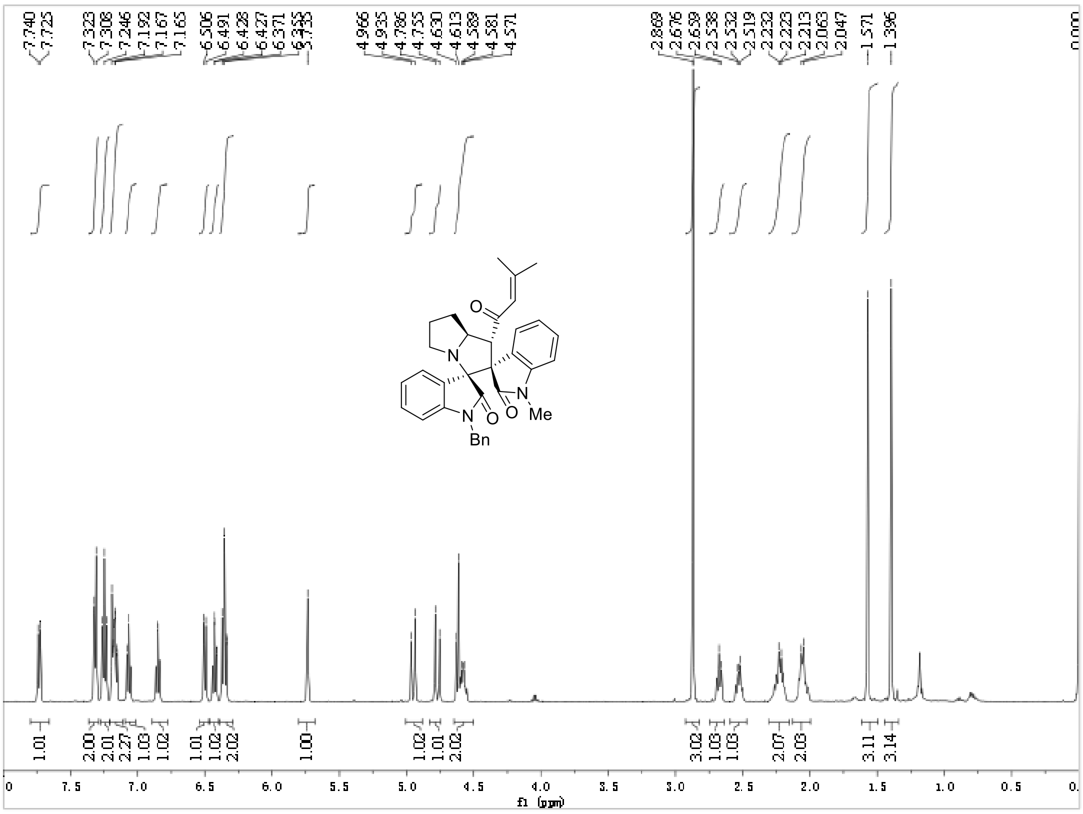

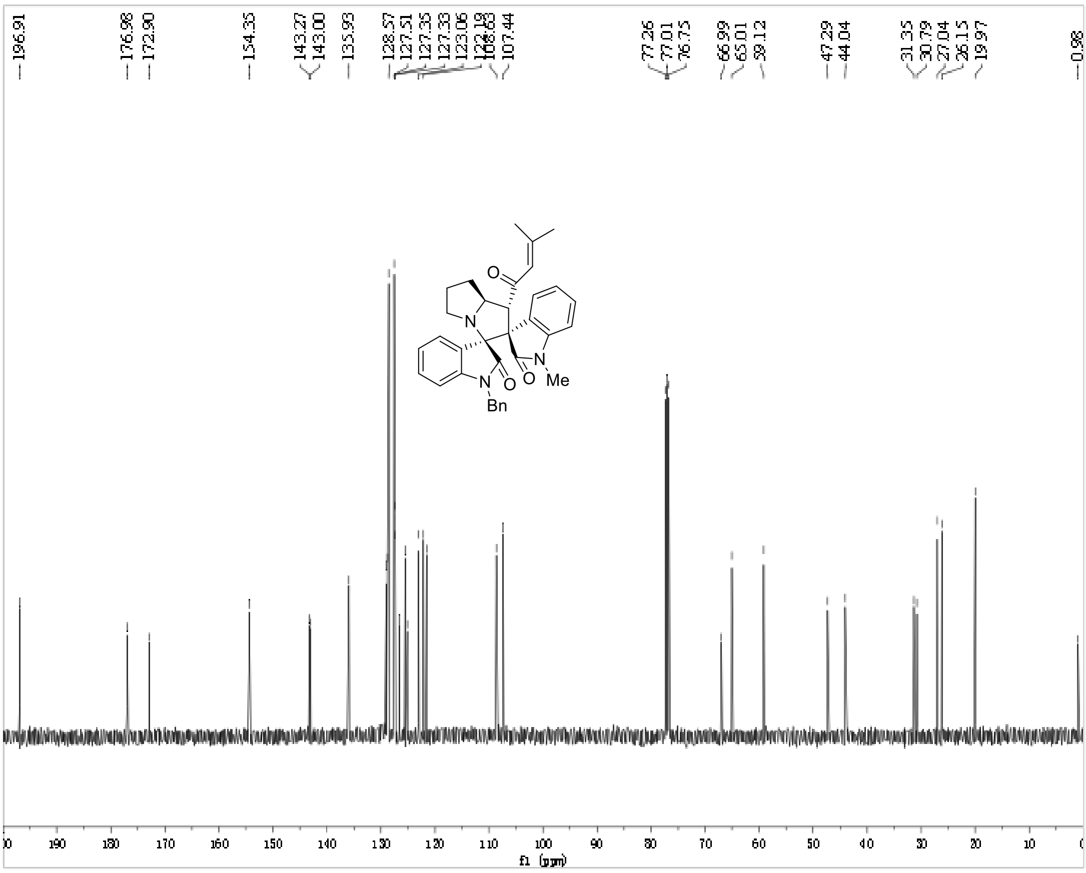

^{1}H and ^{13}C NMR of 3ca

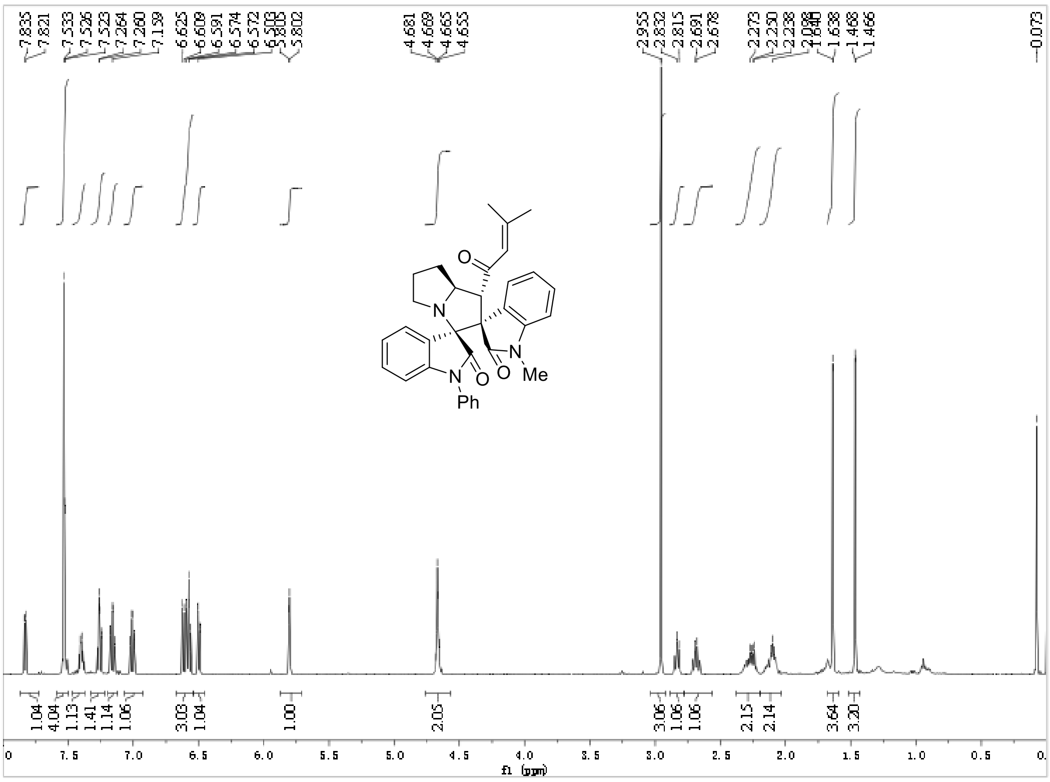

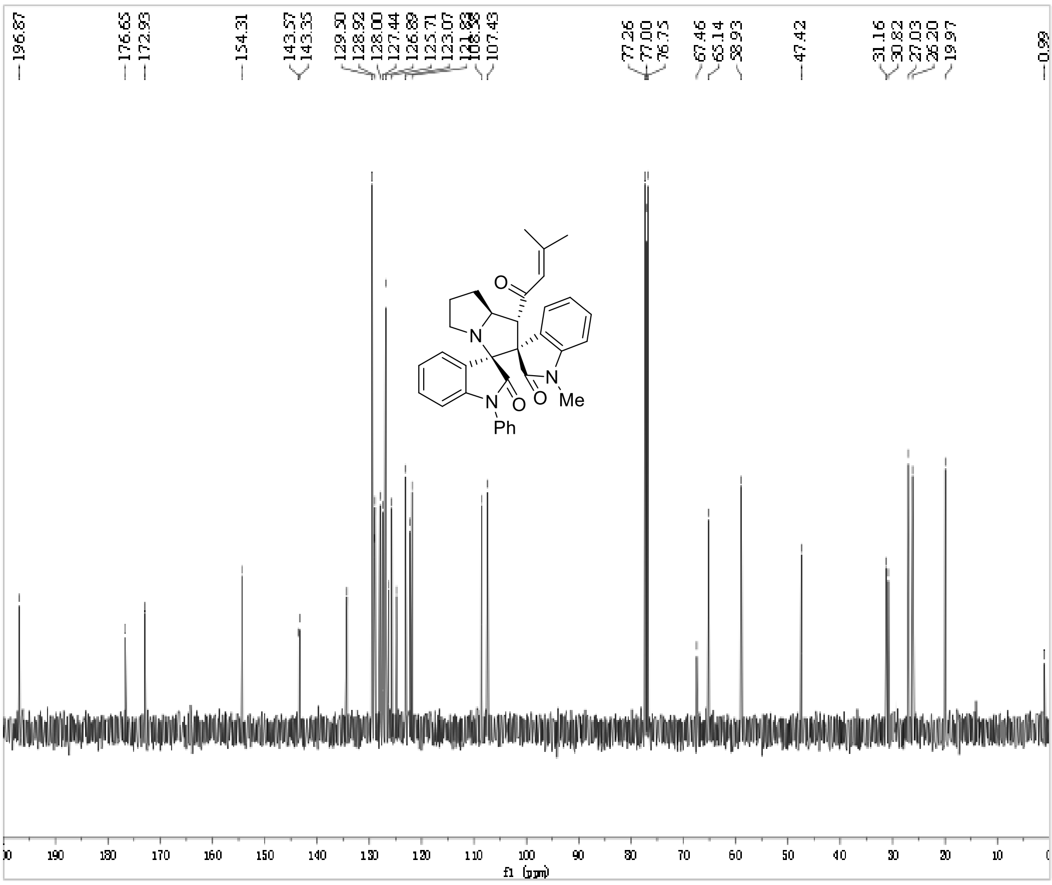

^{1}H and ^{13}C NMR of 3da

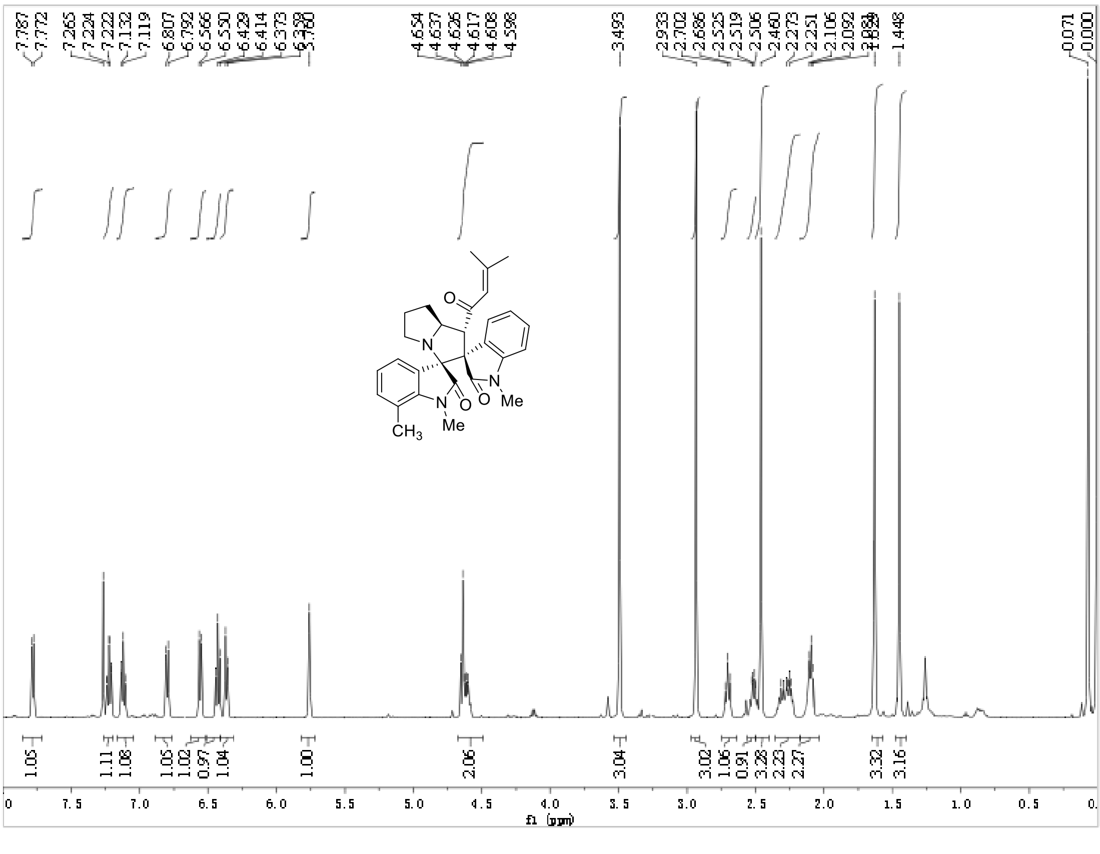

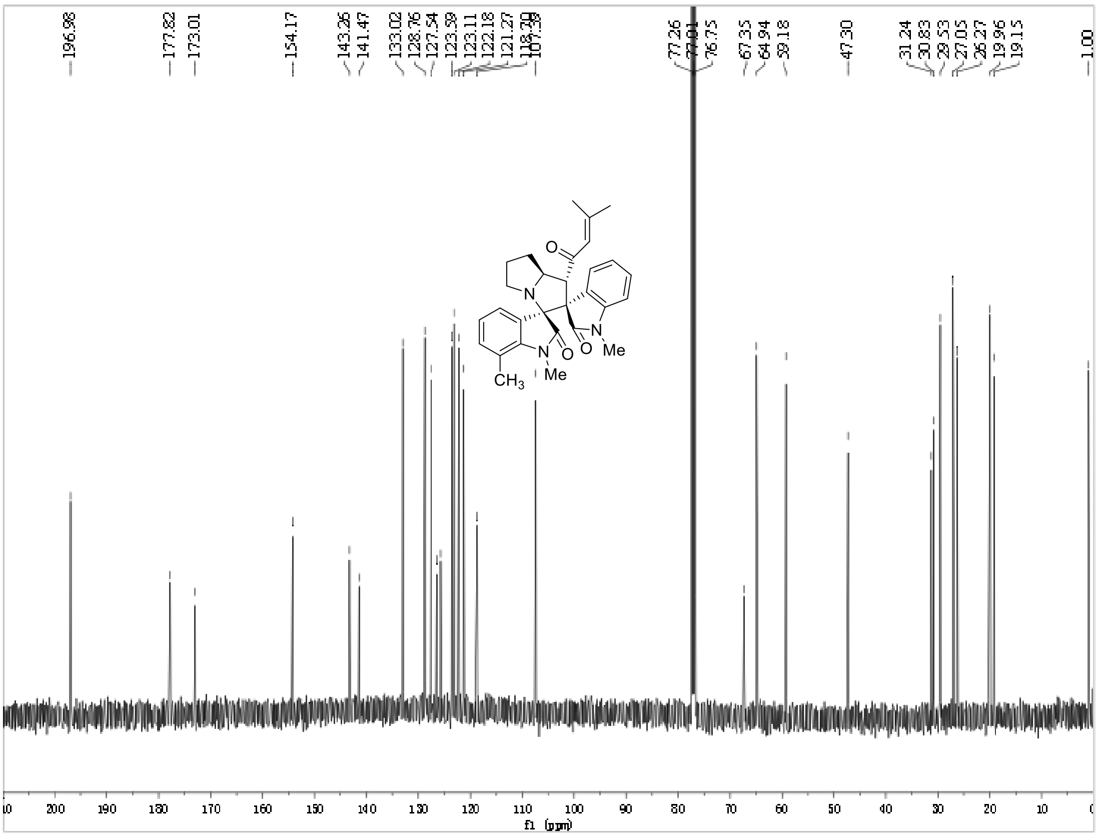

^{1}H and ^{13}C NMR of 3ea

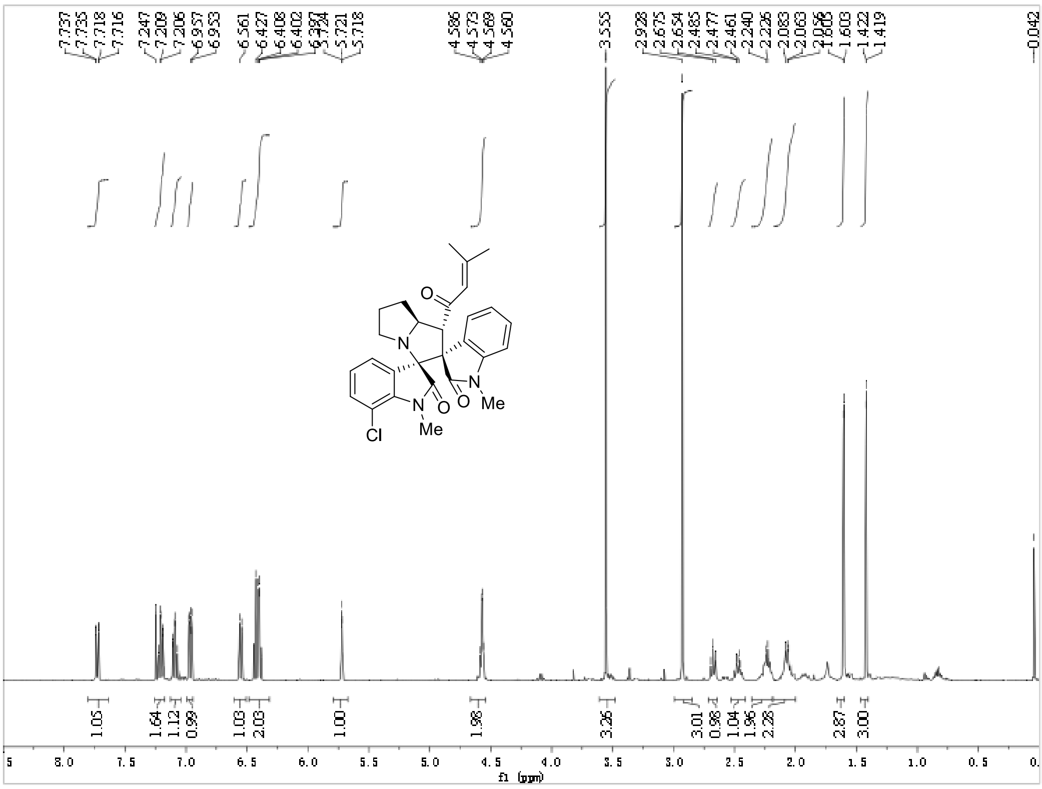

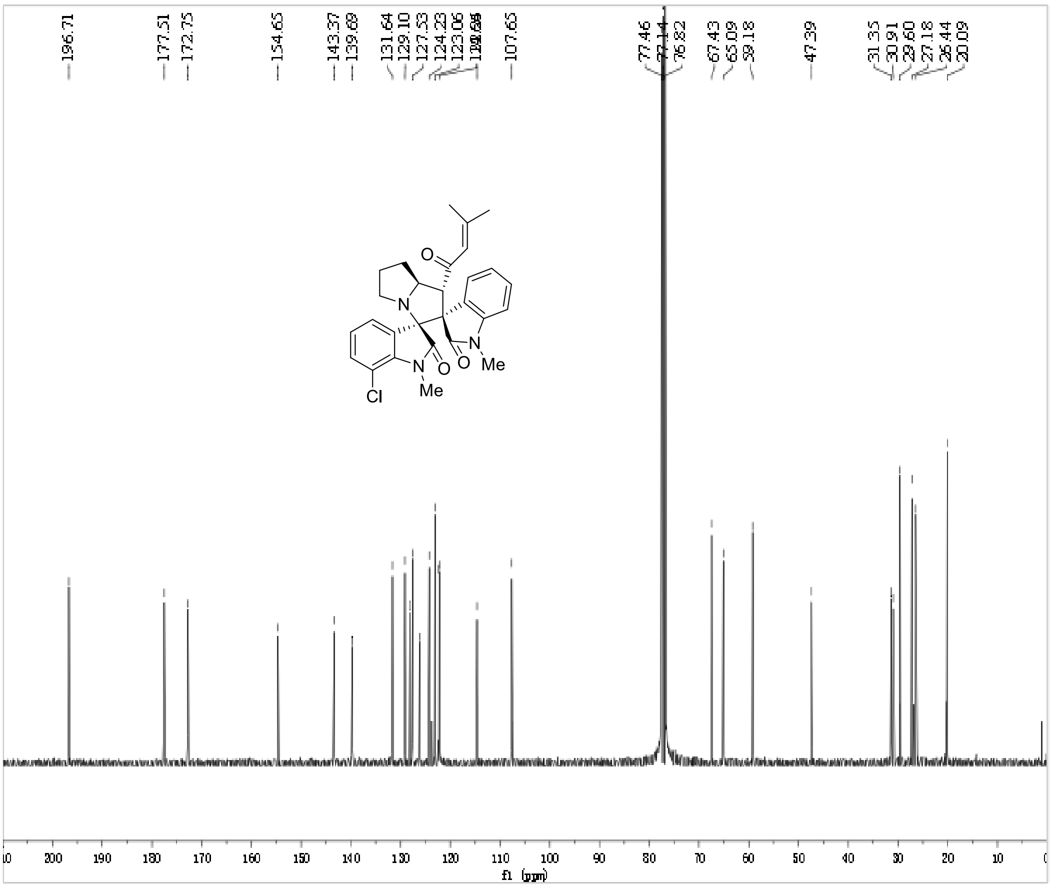

^{1}H and ^{13}C NMR of 3fa

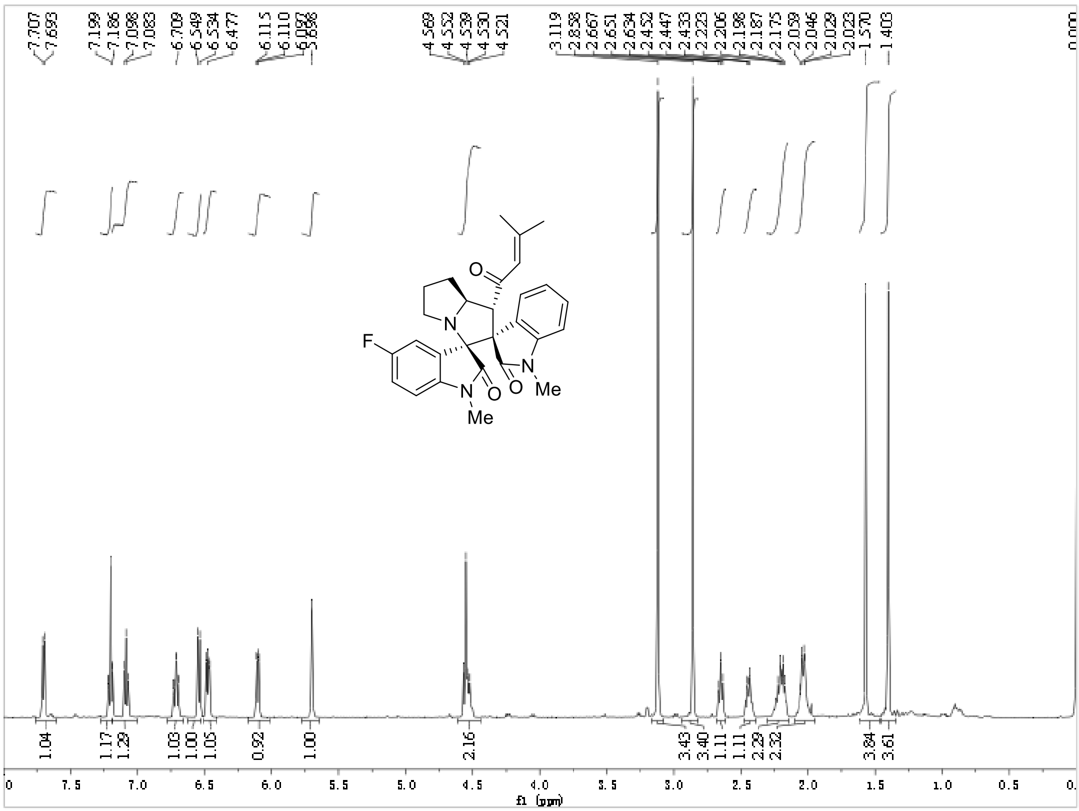

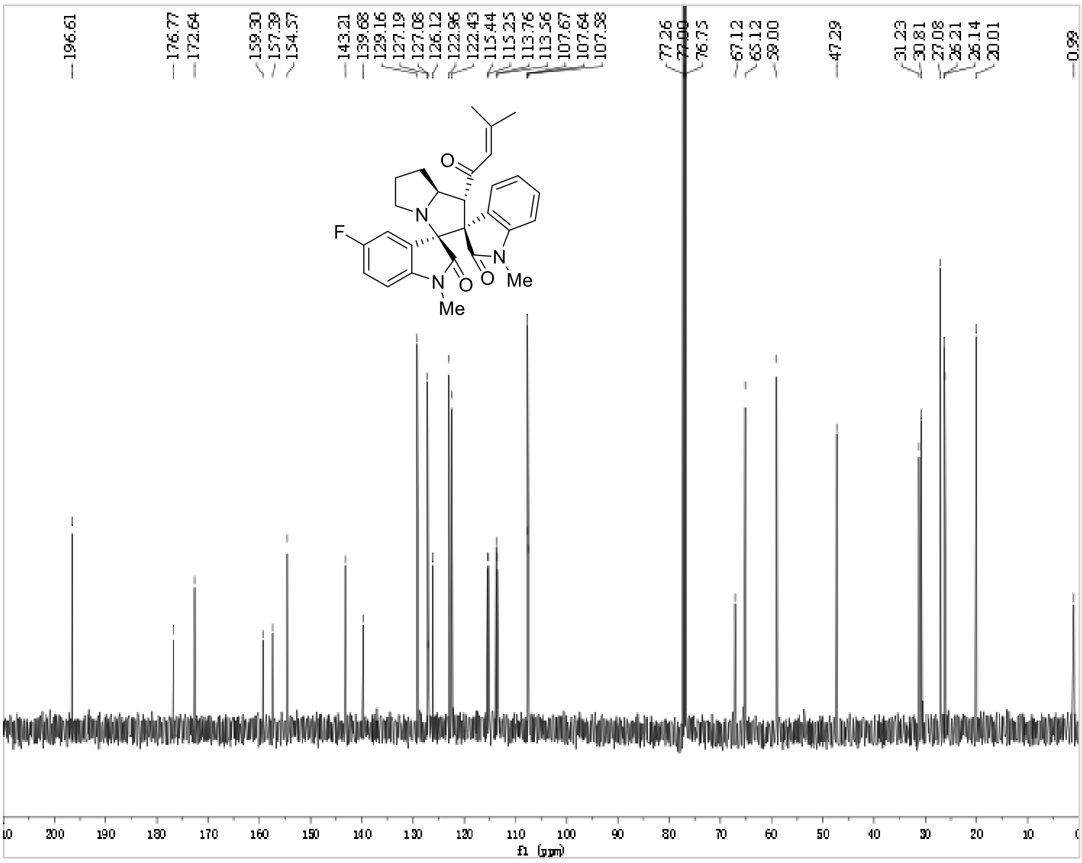

^{1}H and ^{13}C NMR of 3ga

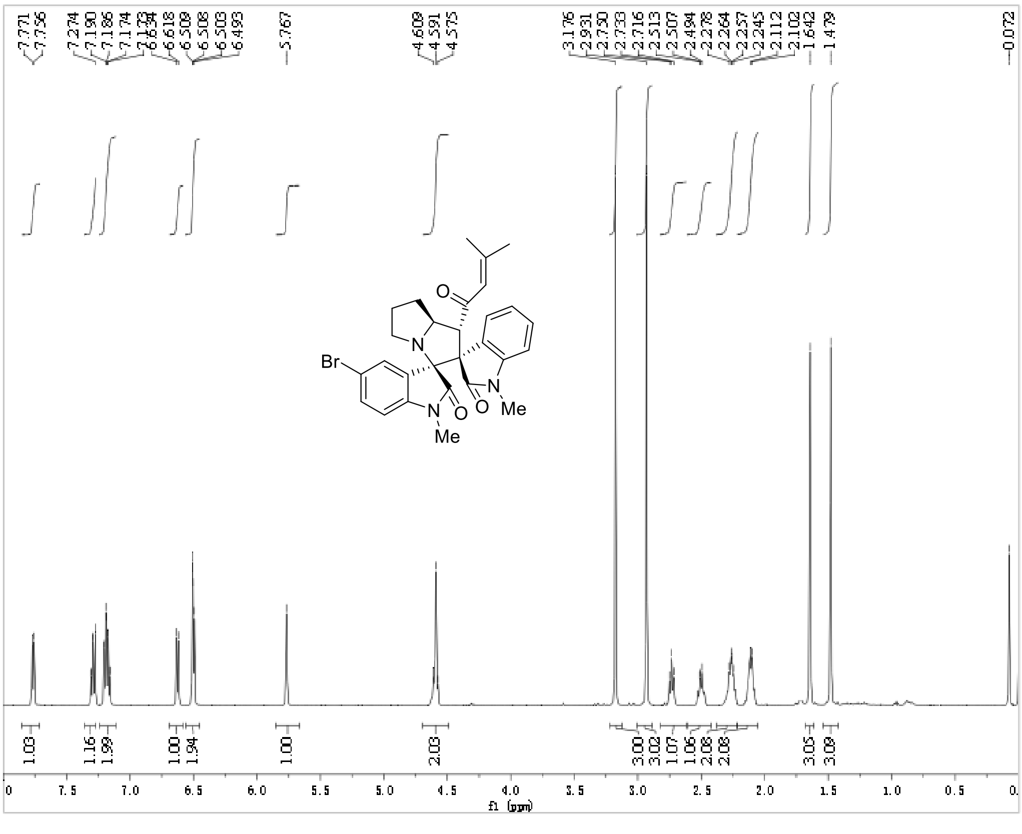

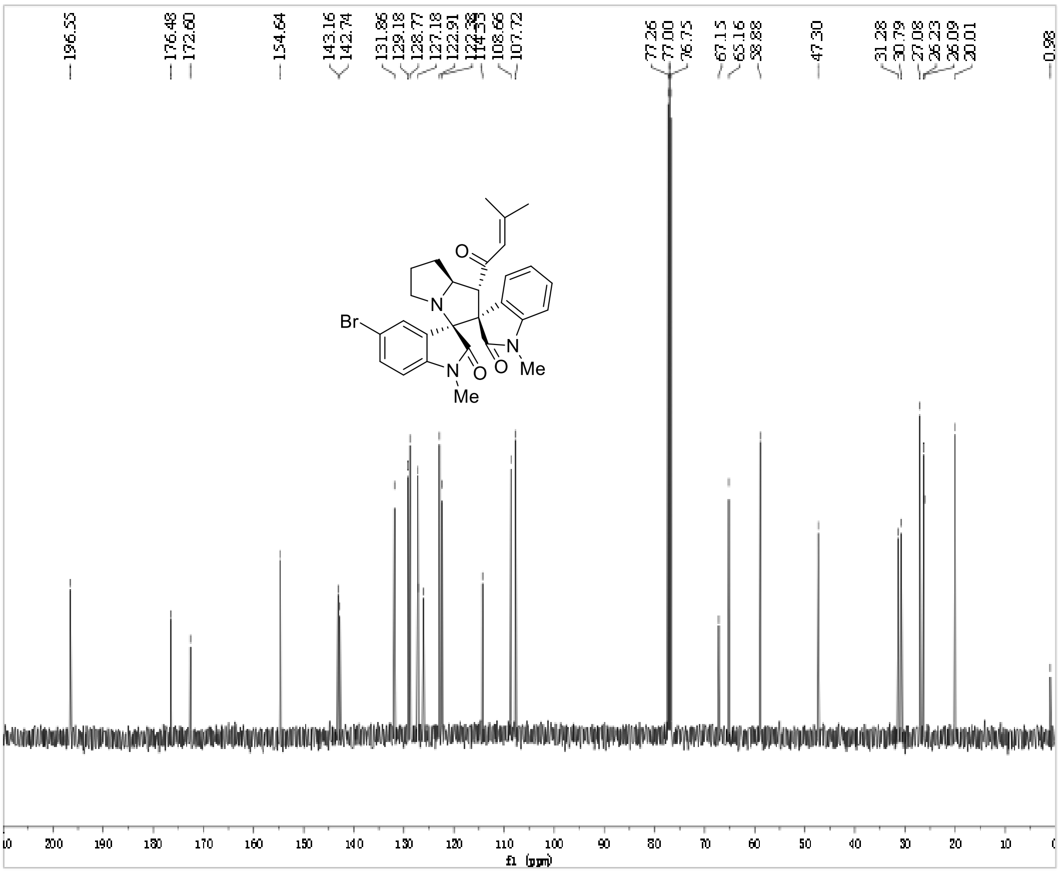

^{1}H and ^{13}C NMR of 3ha

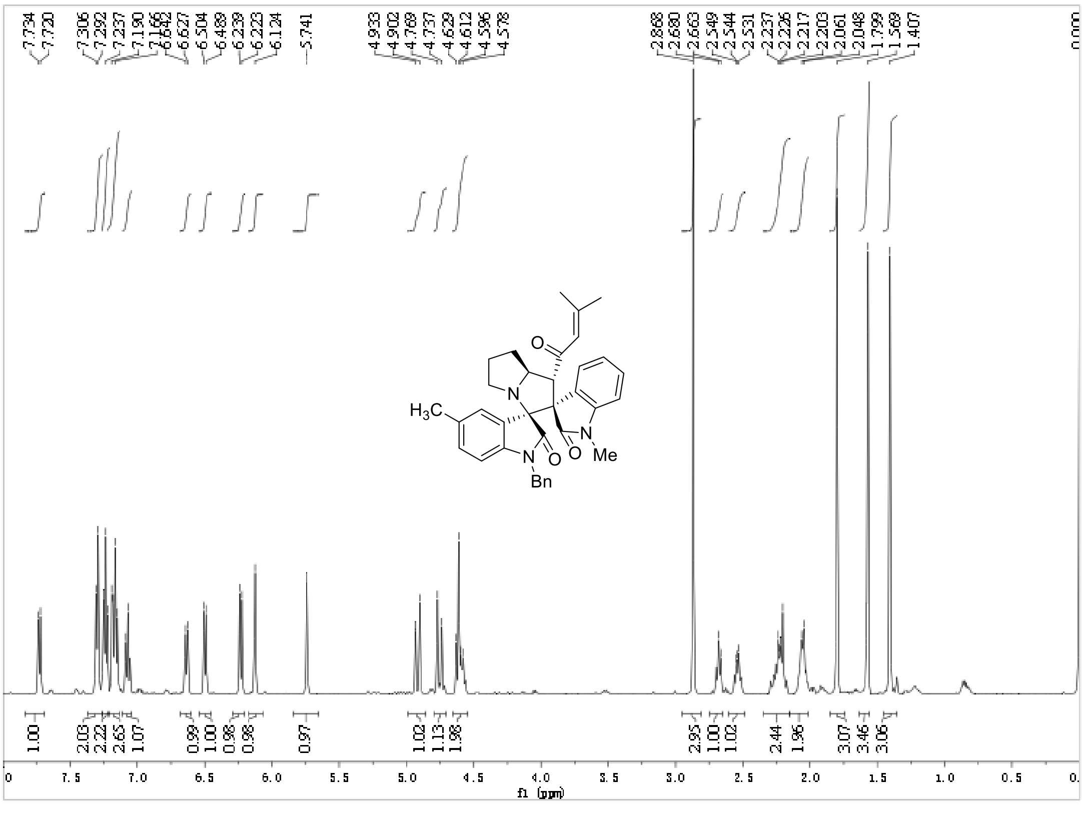

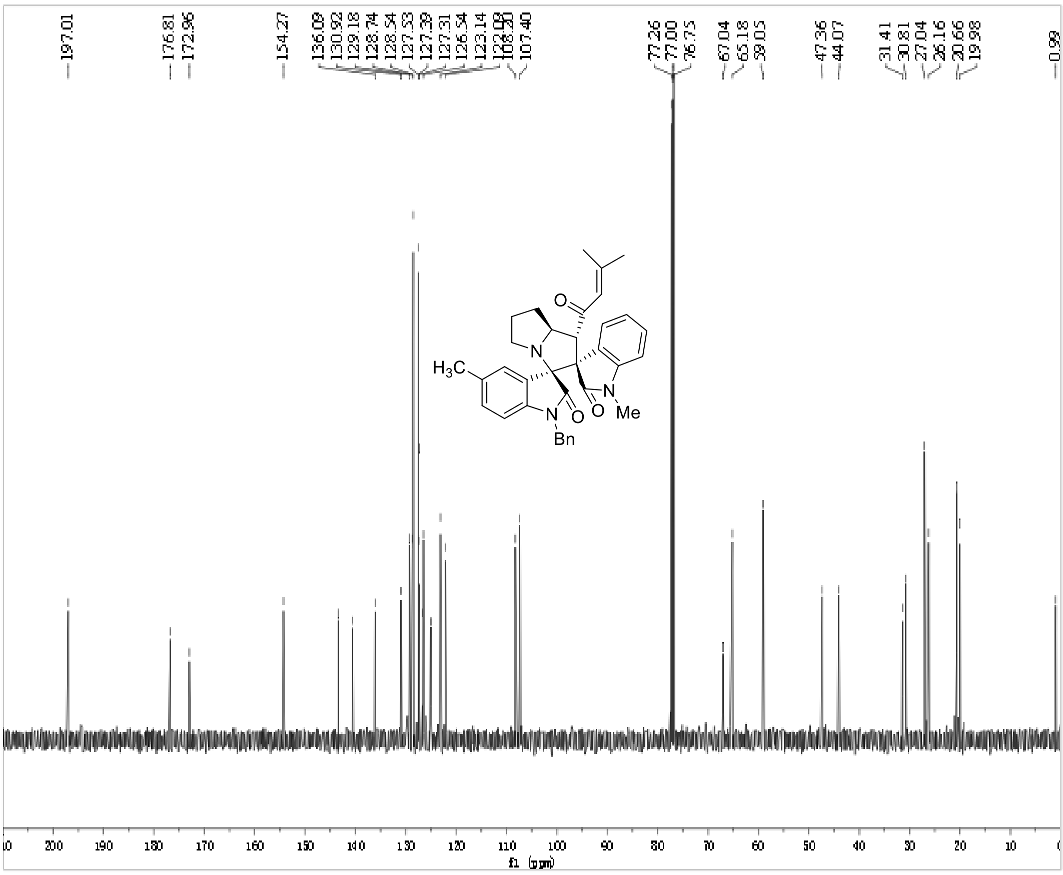

^{1}H and ^{13}C NMR of 3ia

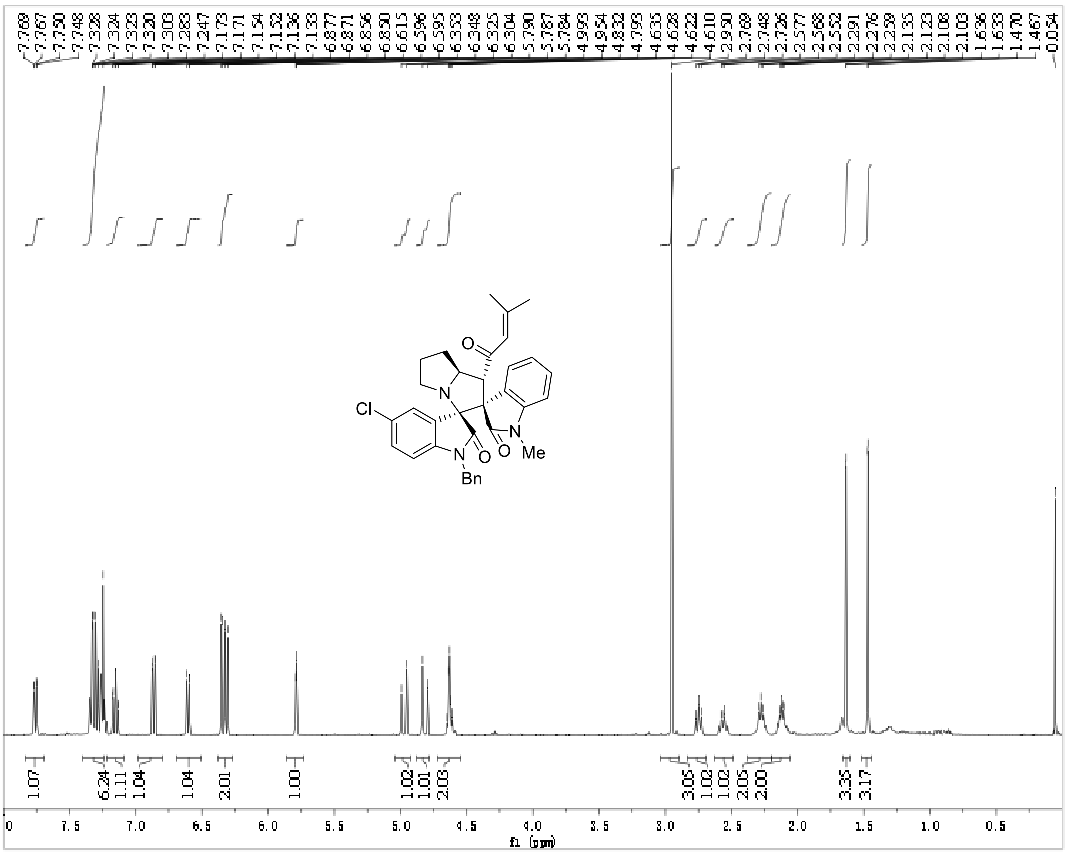

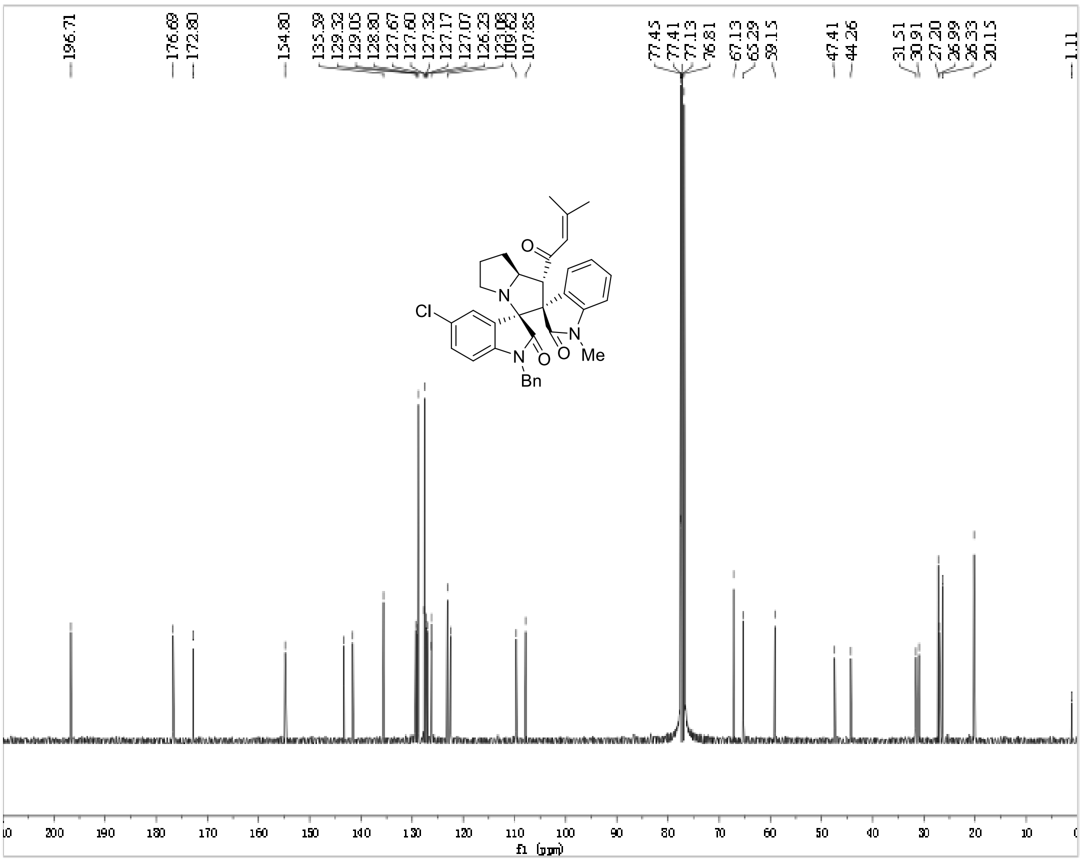

^{1}H and ^{13}C NMR of 3ja

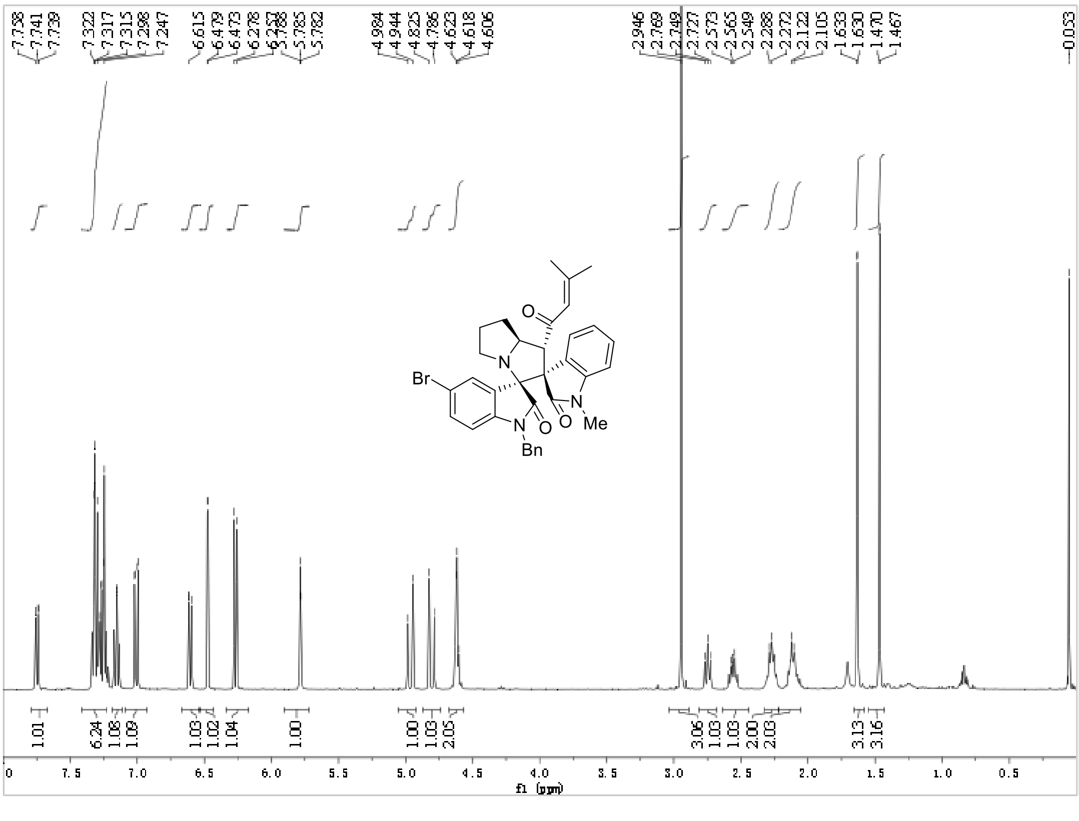

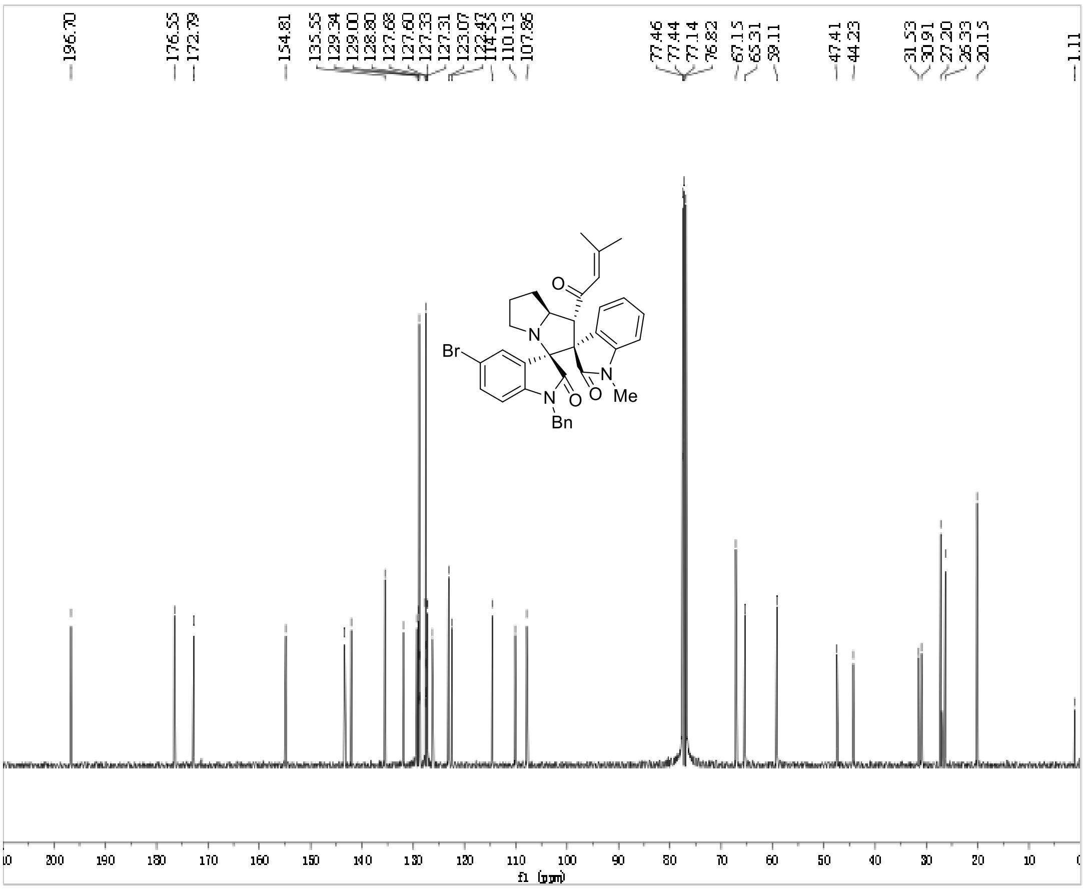

^{1}H and ^{13}C NMR of 3ka

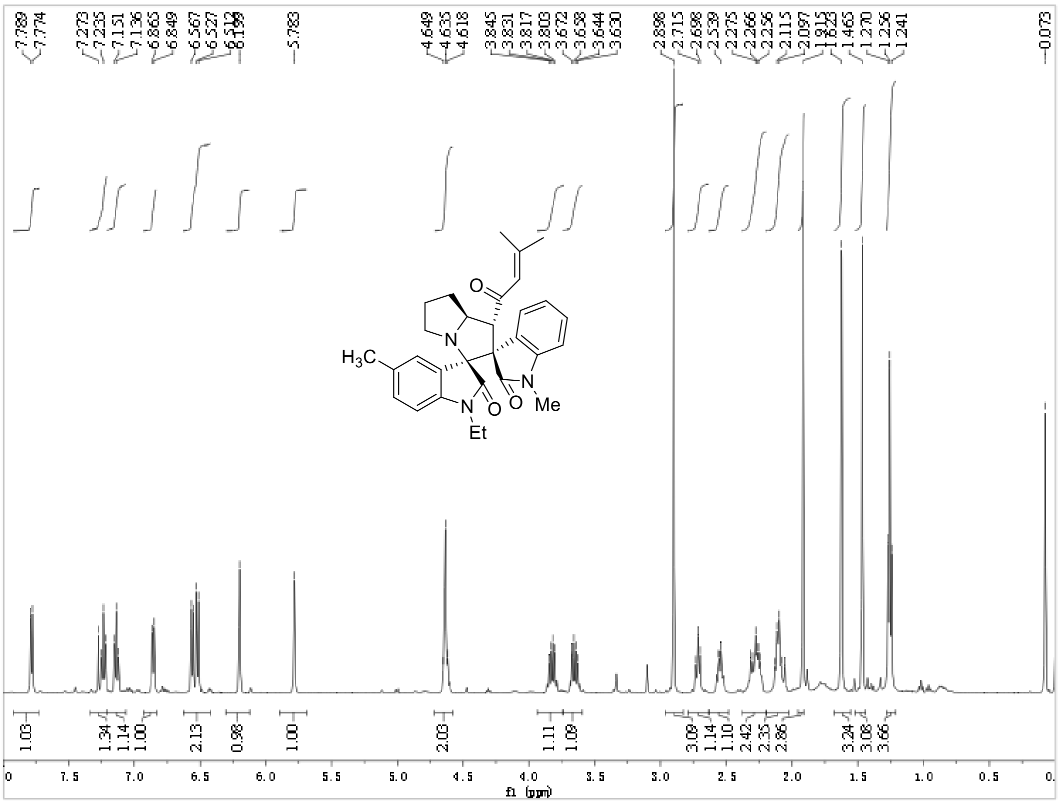

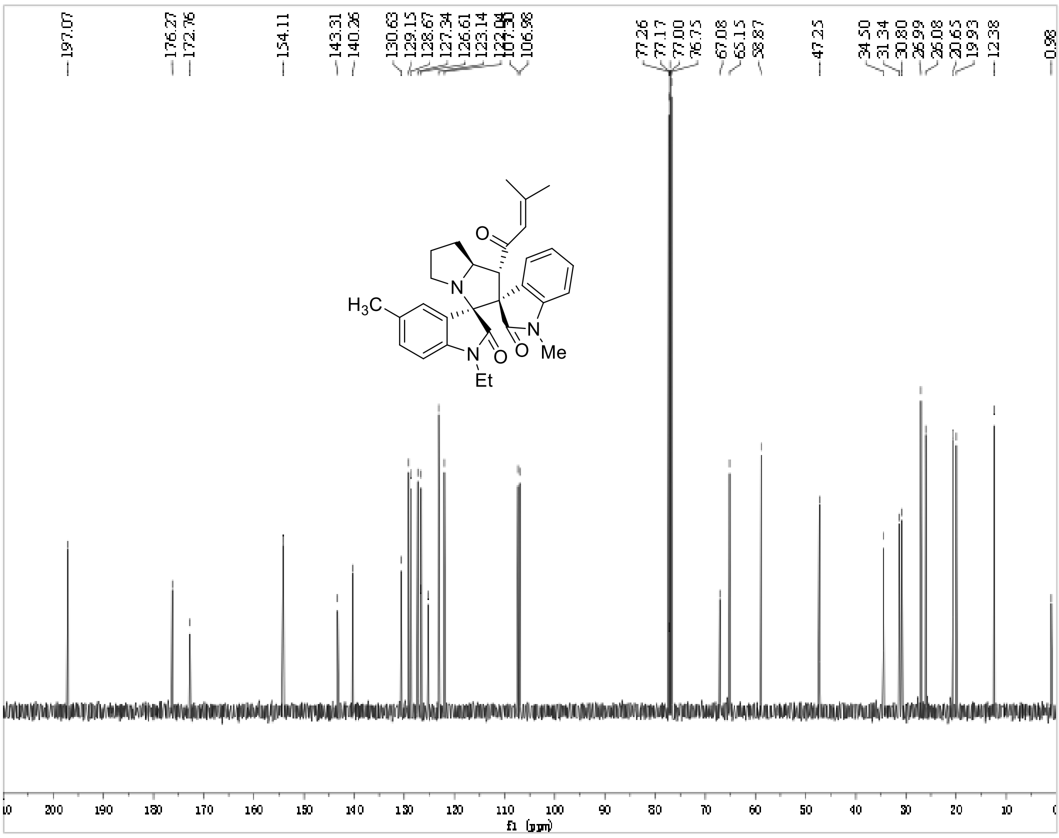

^{1}H and ^{13}C NMR of 3la

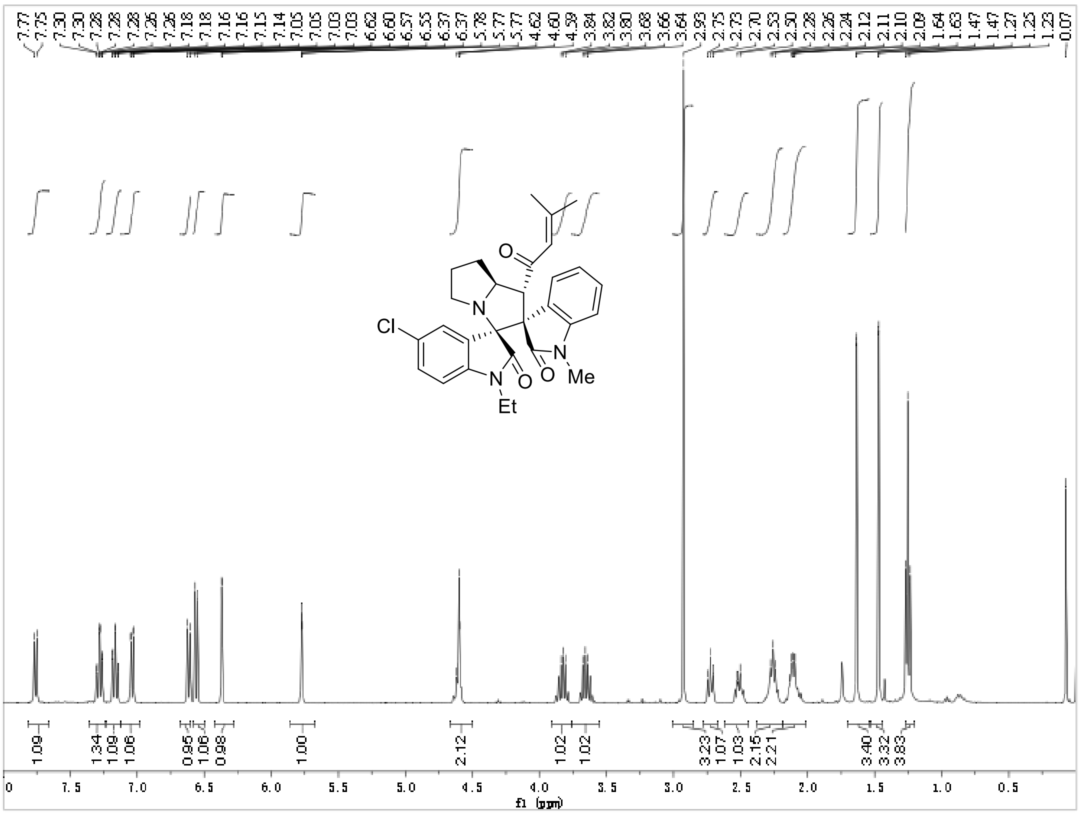

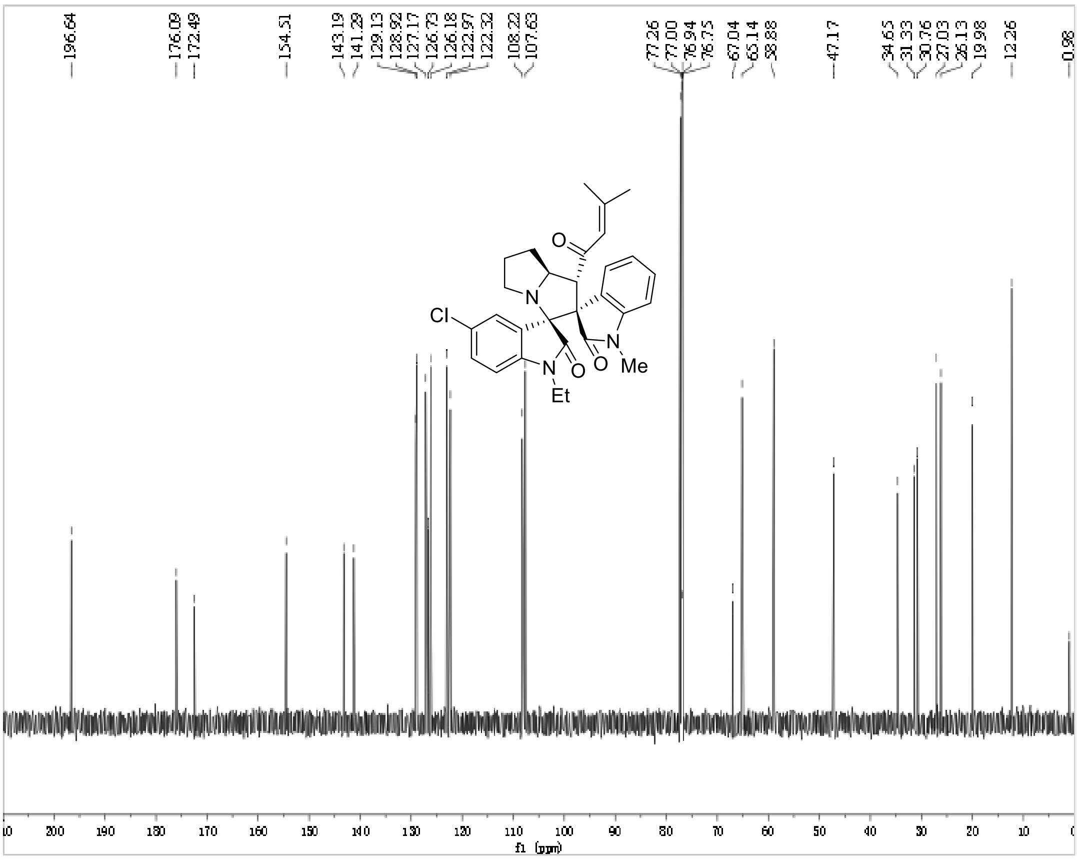

^{1}H and ^{13}C NMR of 3ab

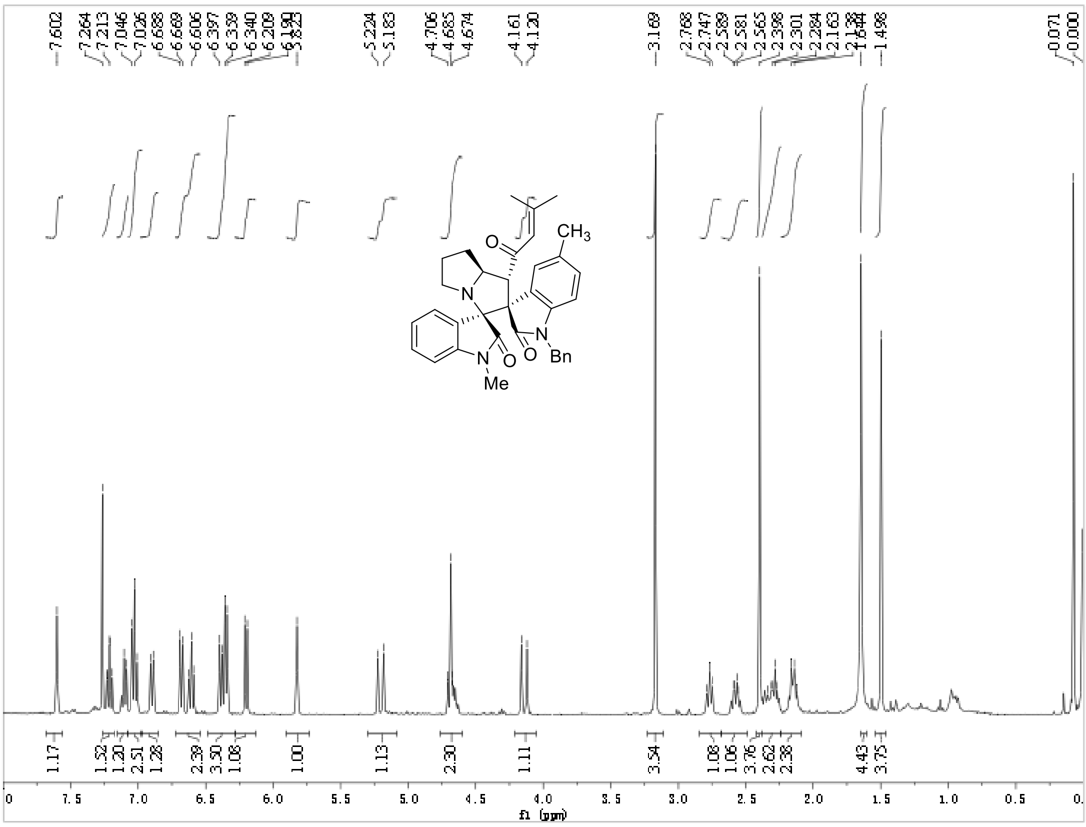

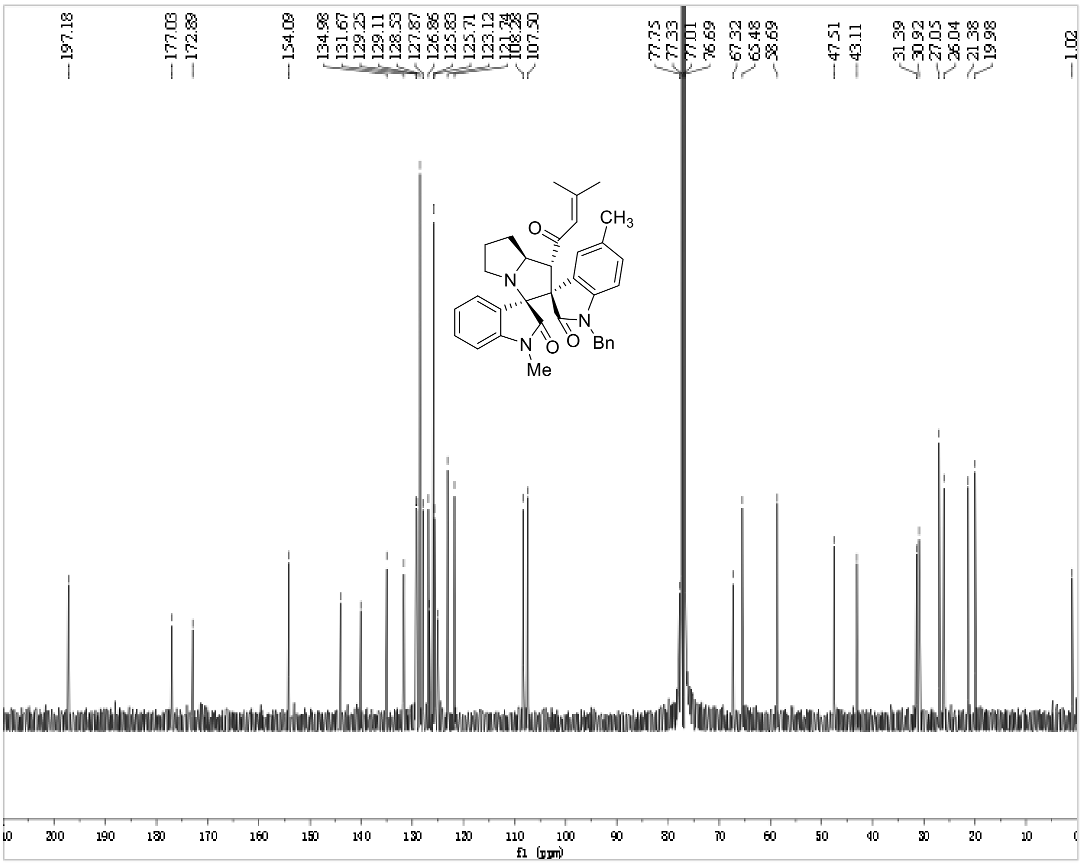

^{1}H and ^{13}C NMR of 3ac

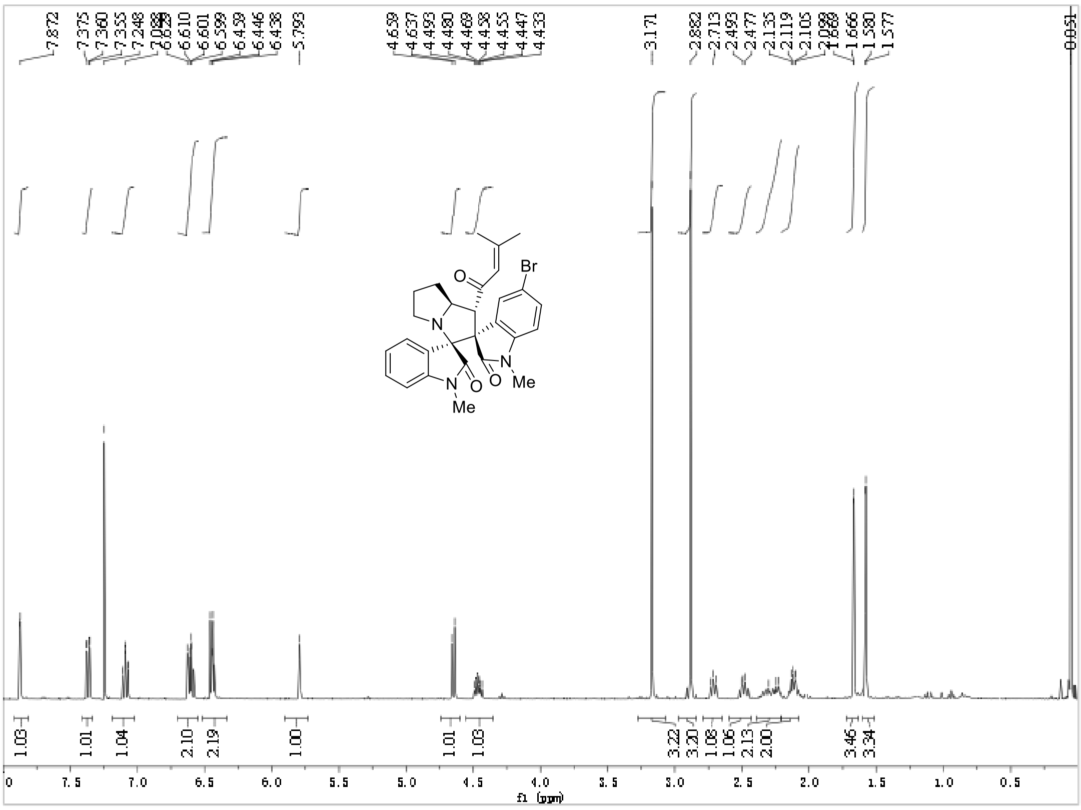

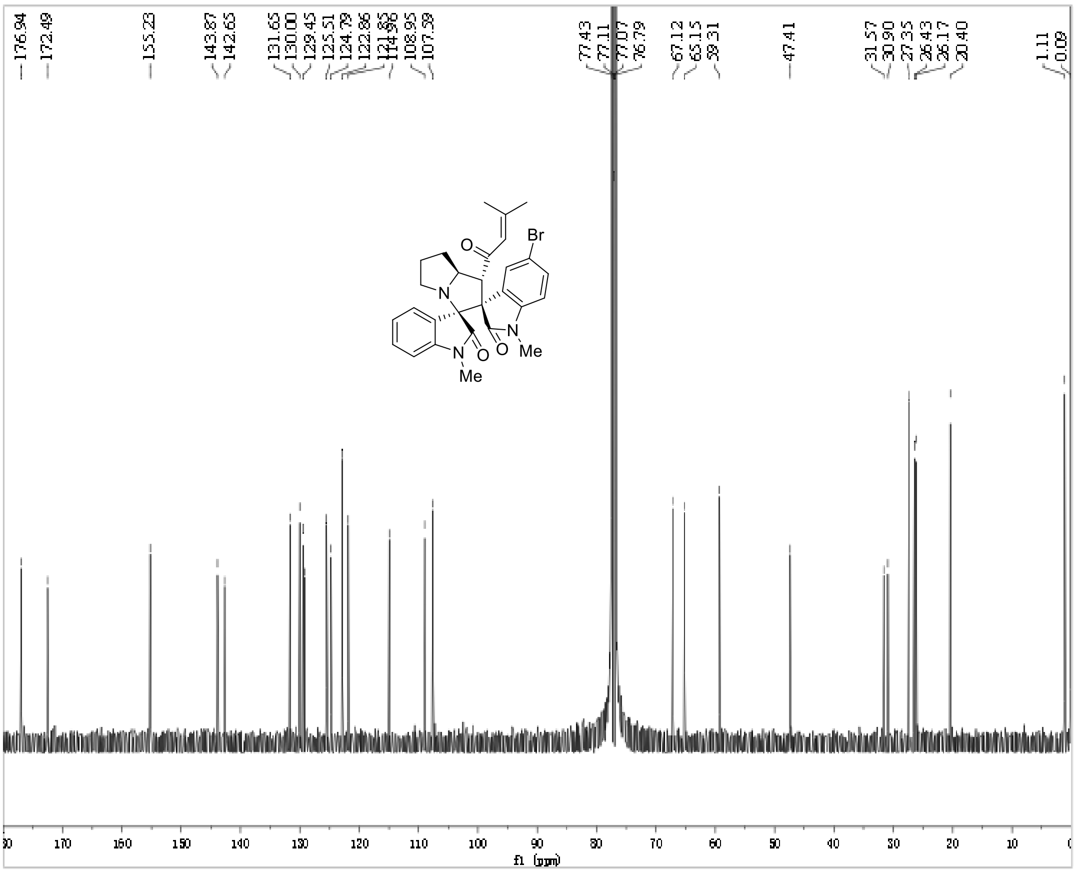

^{1}H and ^{13}C NMR of 3dd

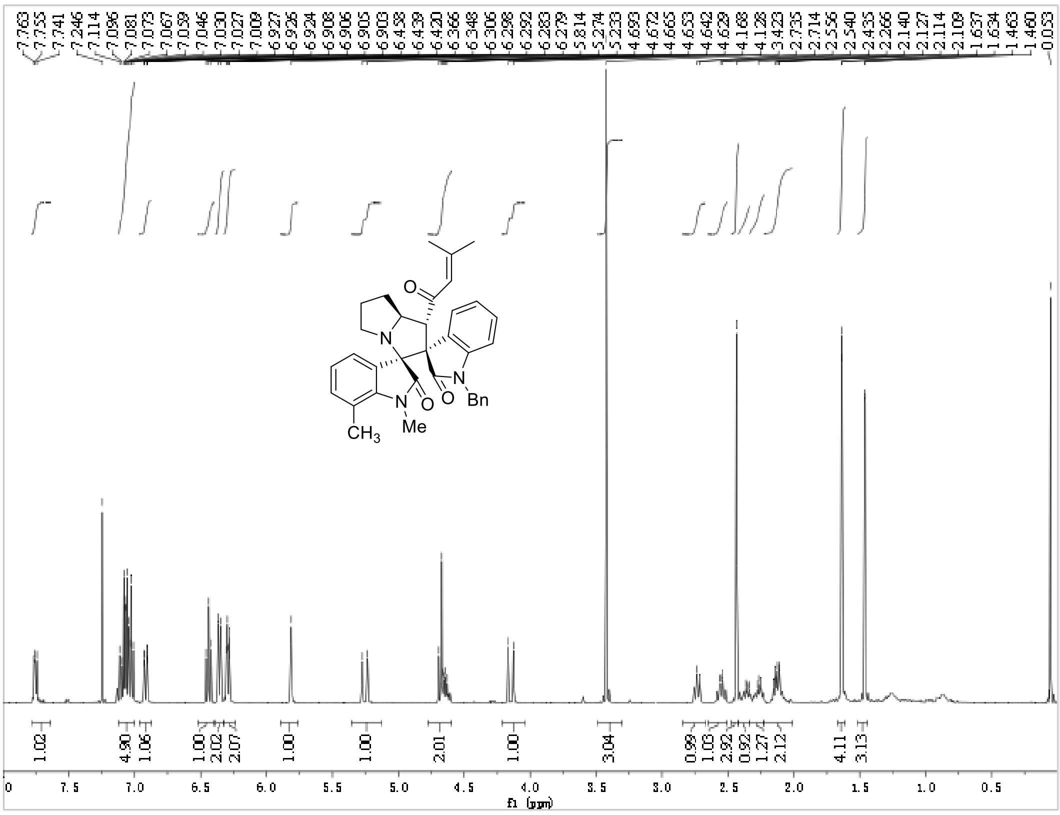

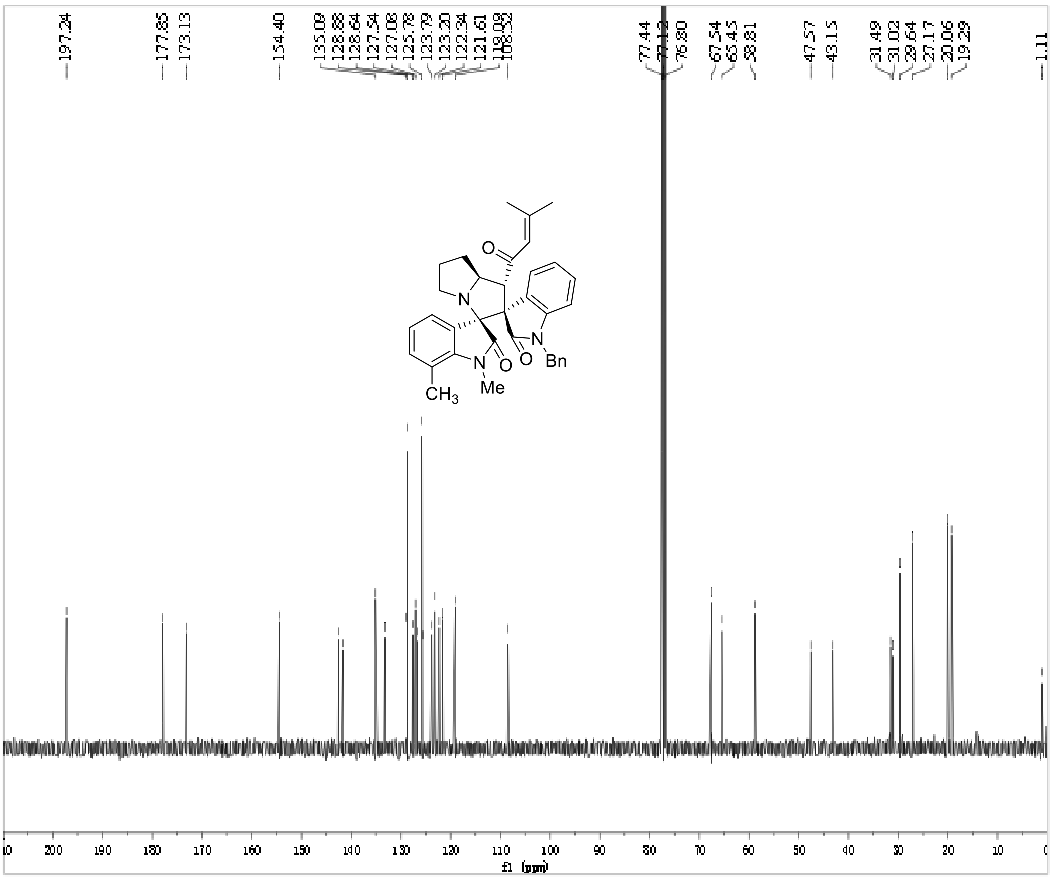

^{1}H and ^{13}C NMR of 3fd

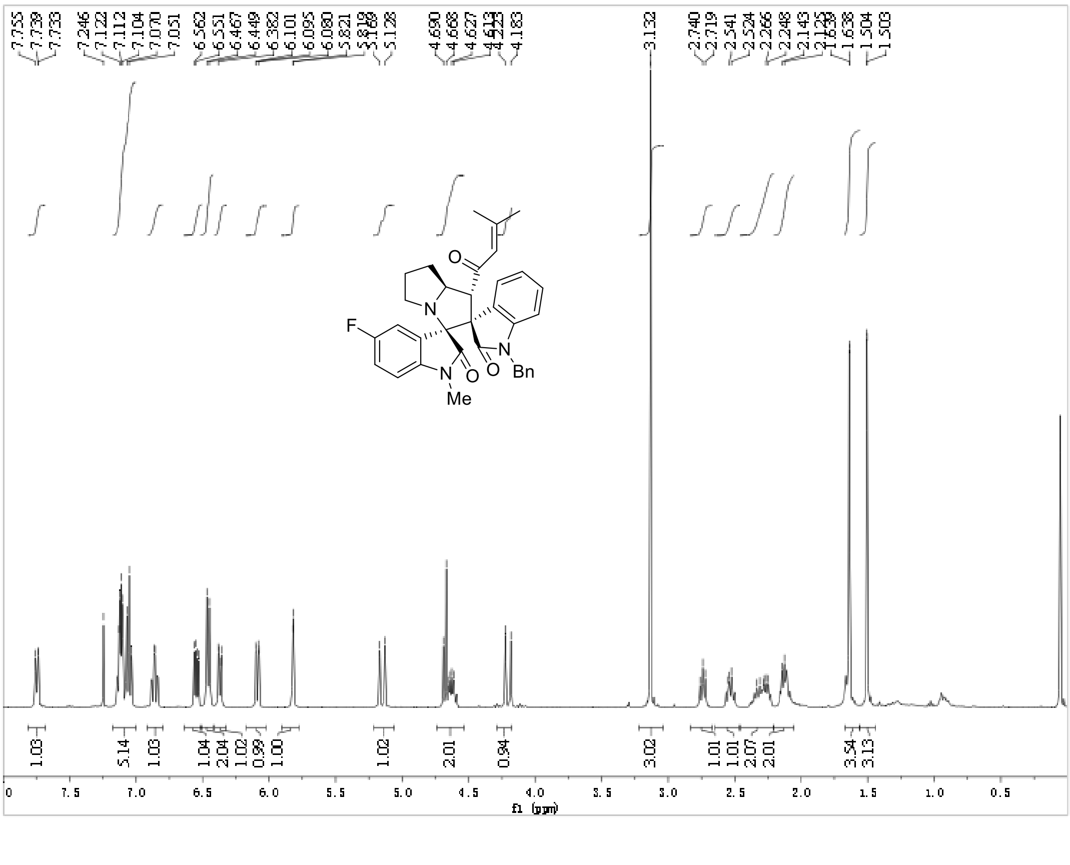

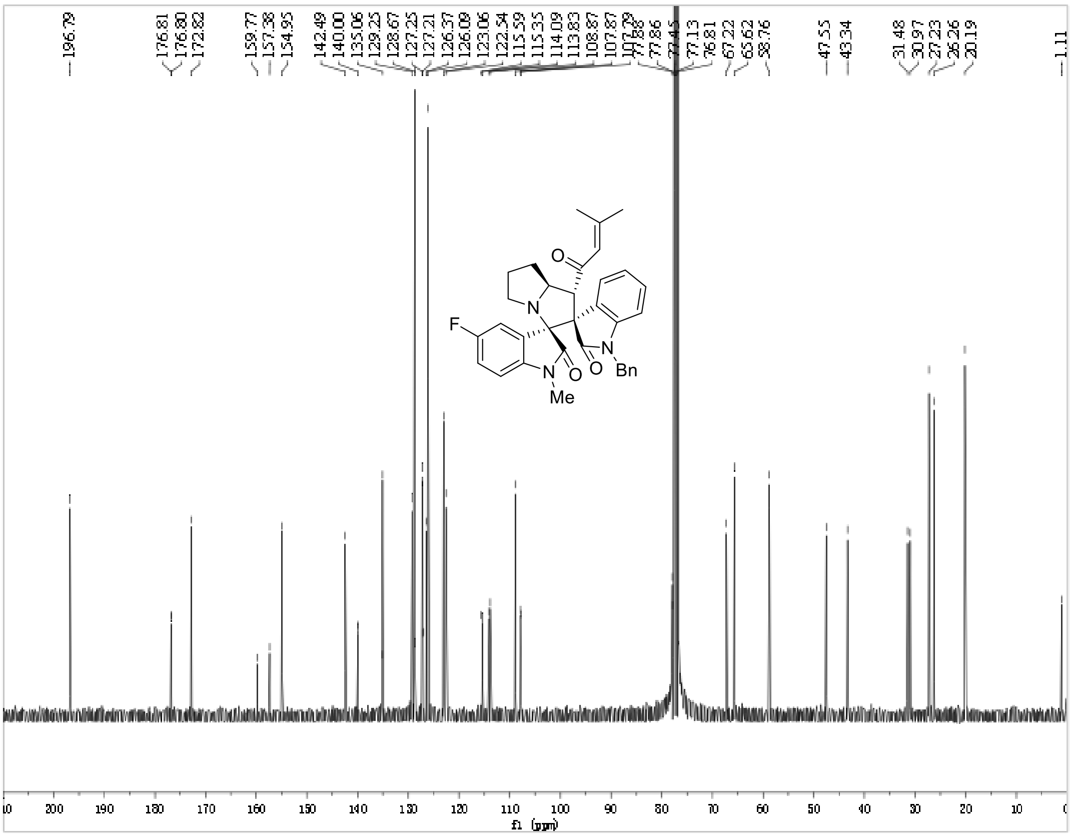

^{1}H and ^{13}C NMR of 3if

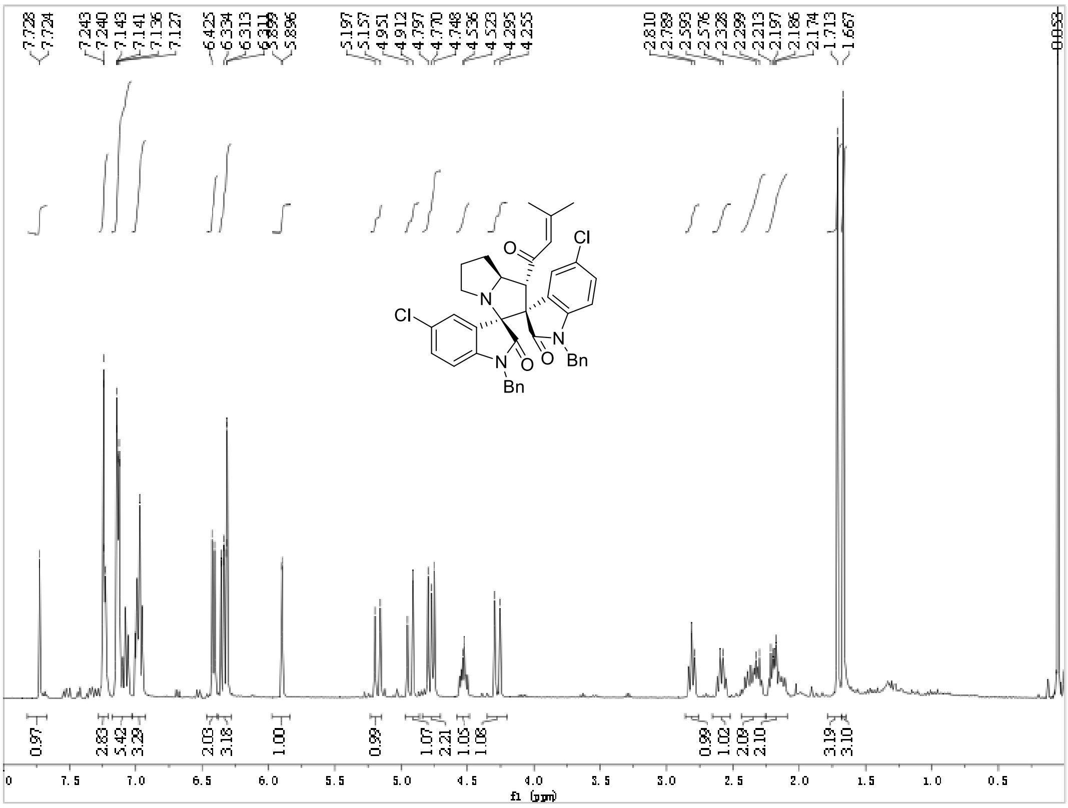

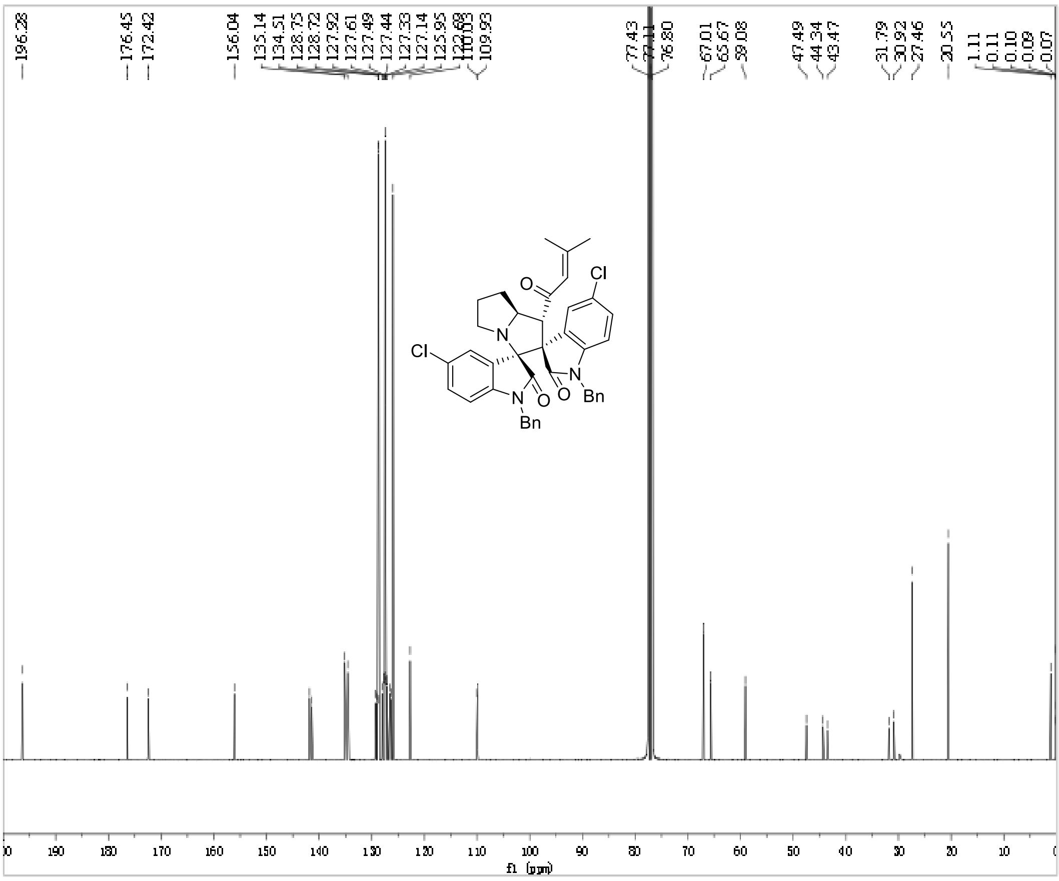

^{1}H and ^{13}C NMR of 3le

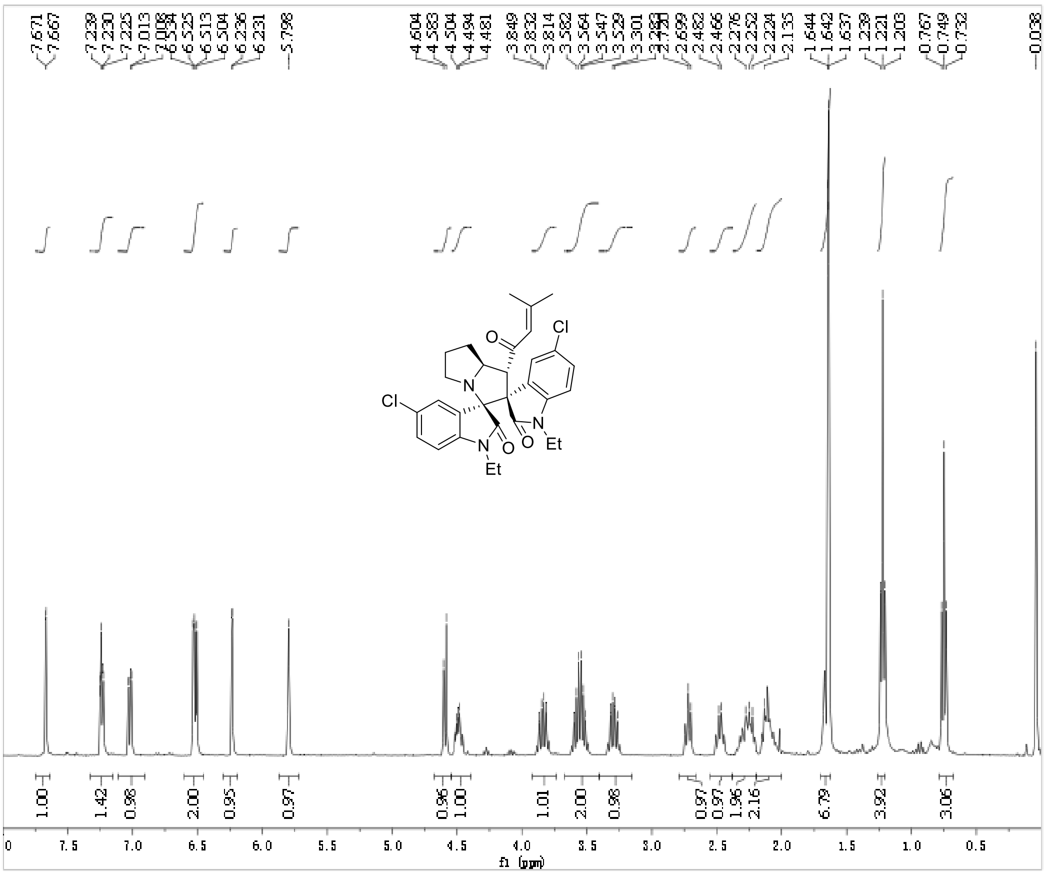

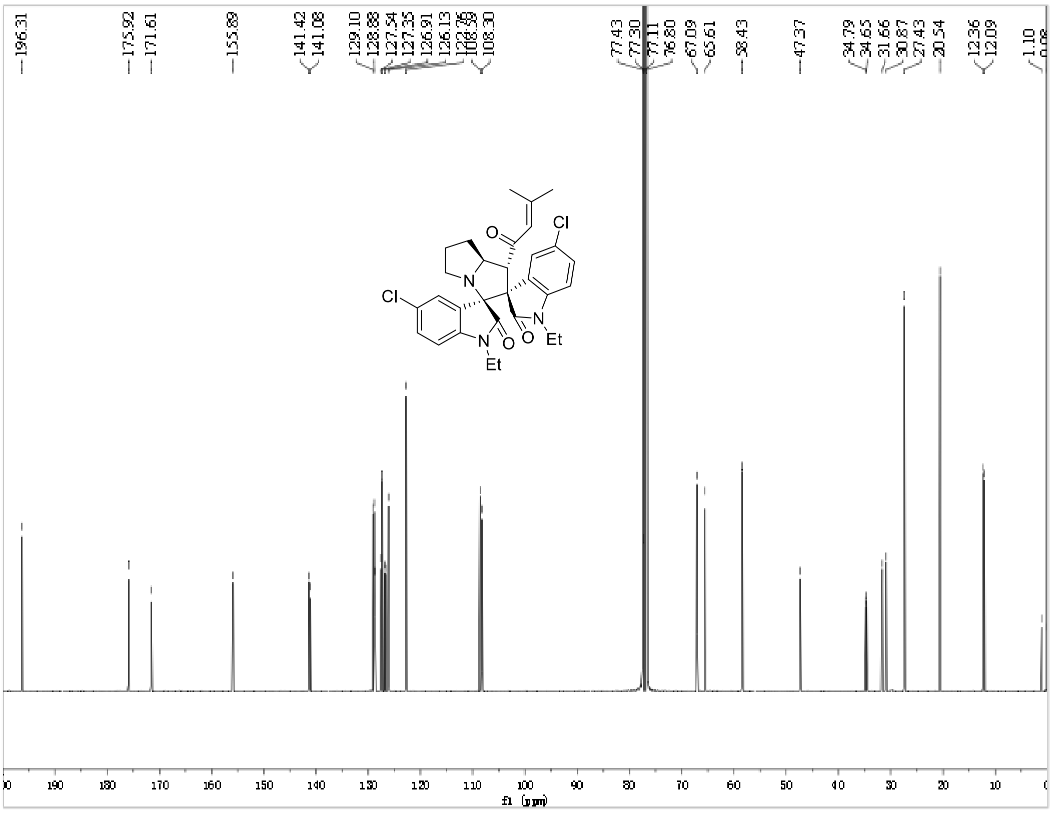

^{1}H and ^{13}C NMR of 4aa

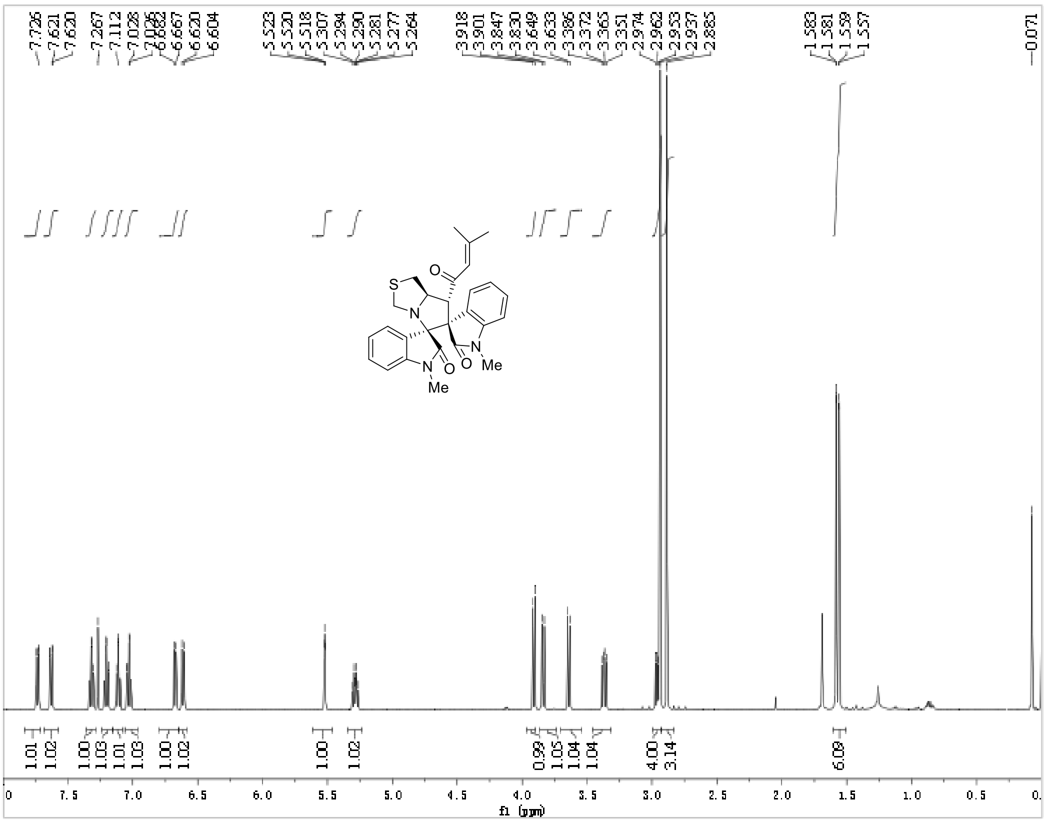

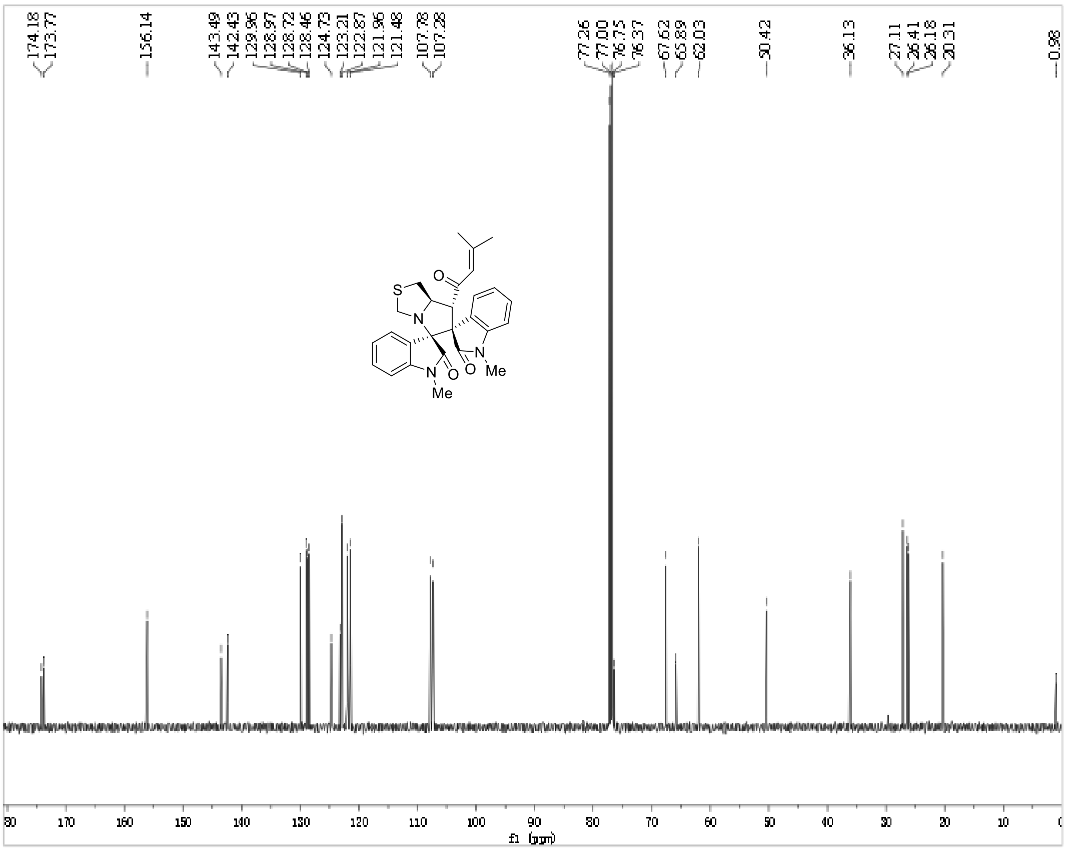

^{1}H and ^{13}C NMR of 4da

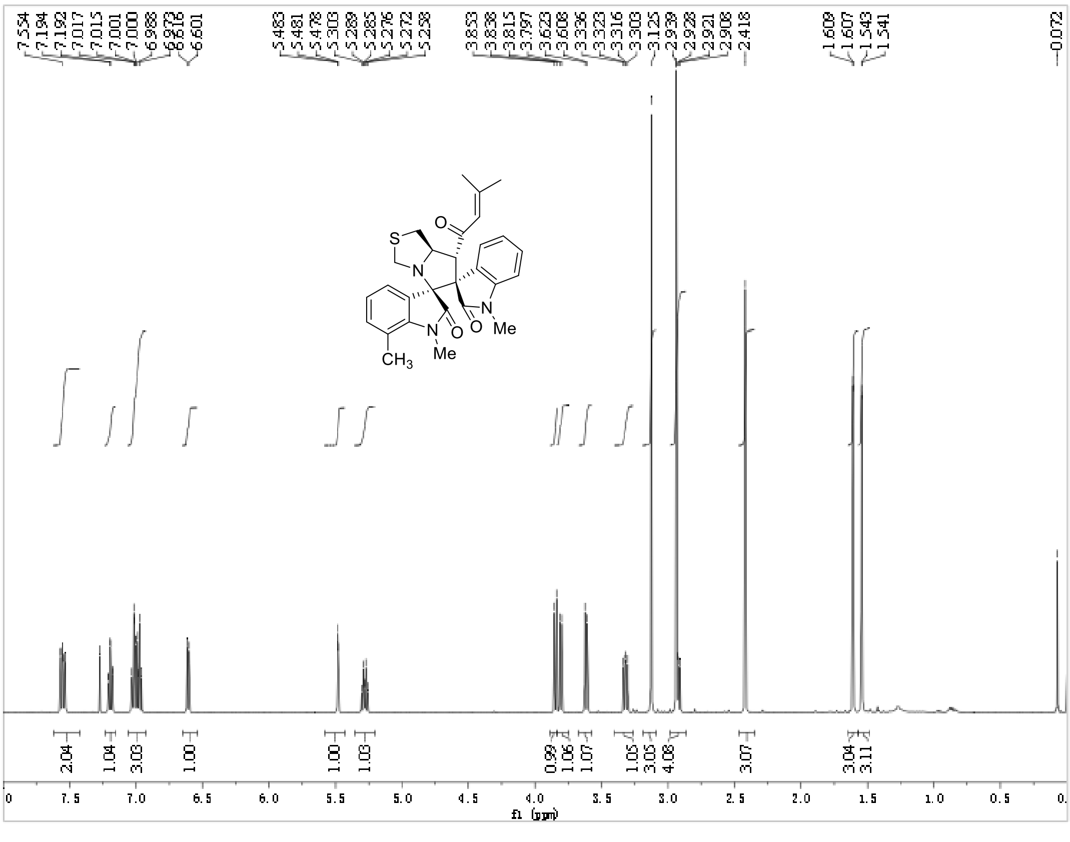

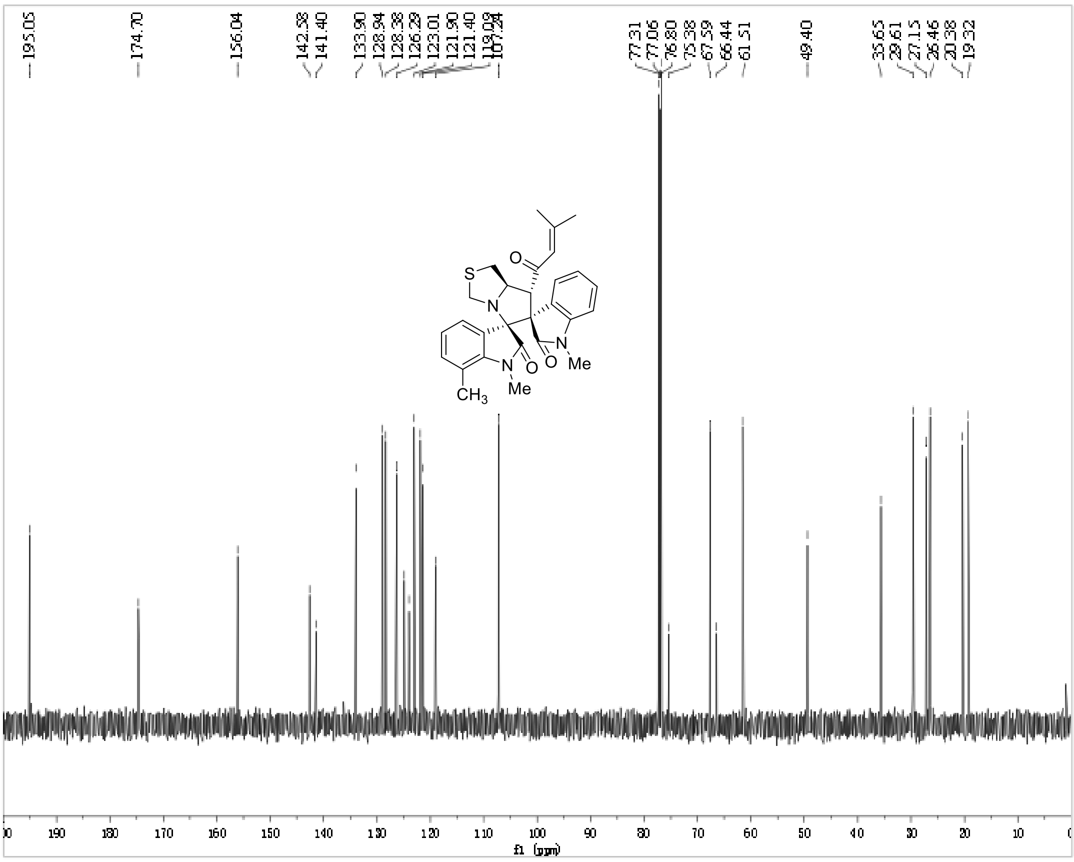

^{1}H and ^{13}C NMR of 4ea

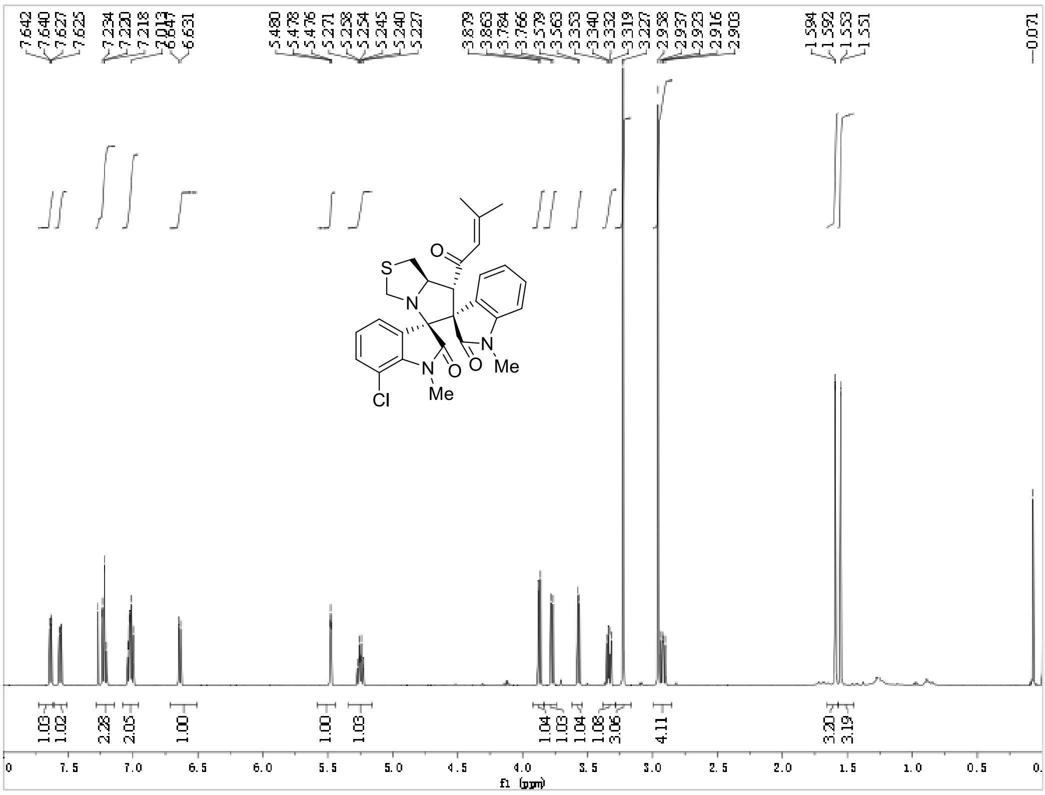

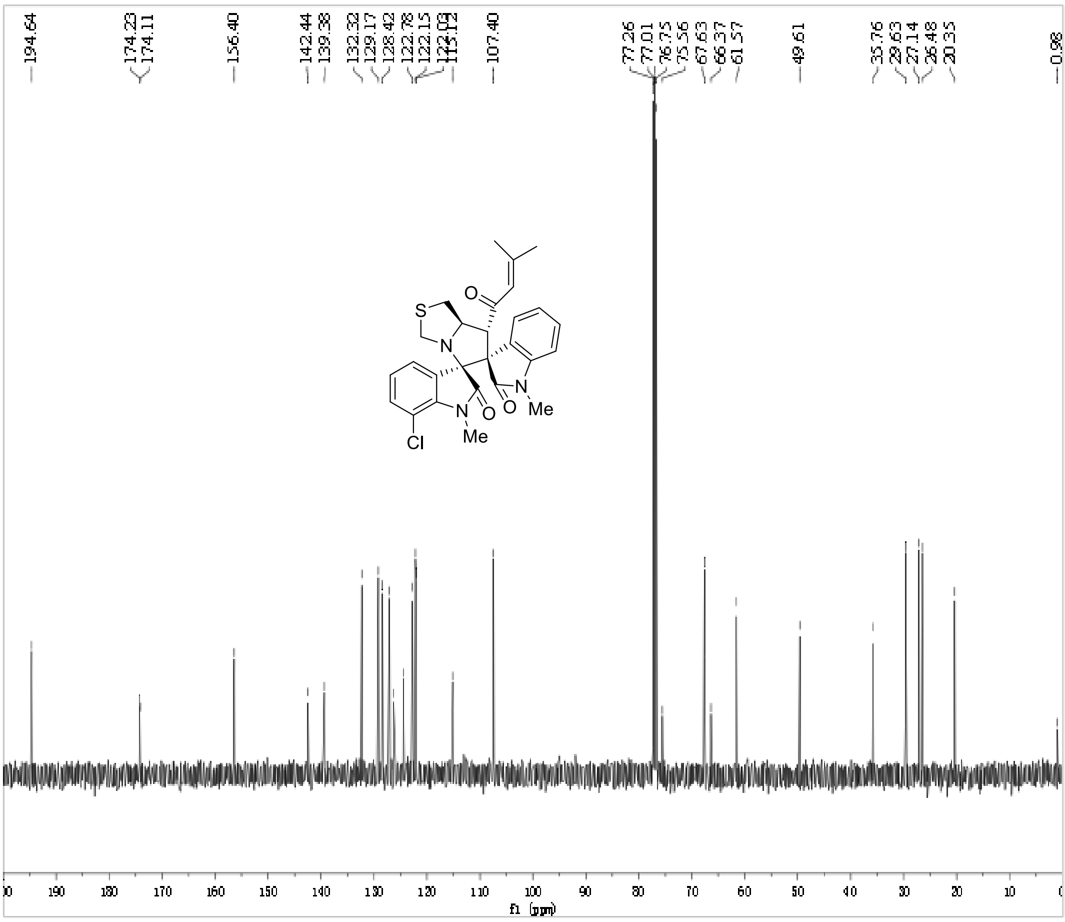

^{1}H and ^{13}C NMR of 4fa

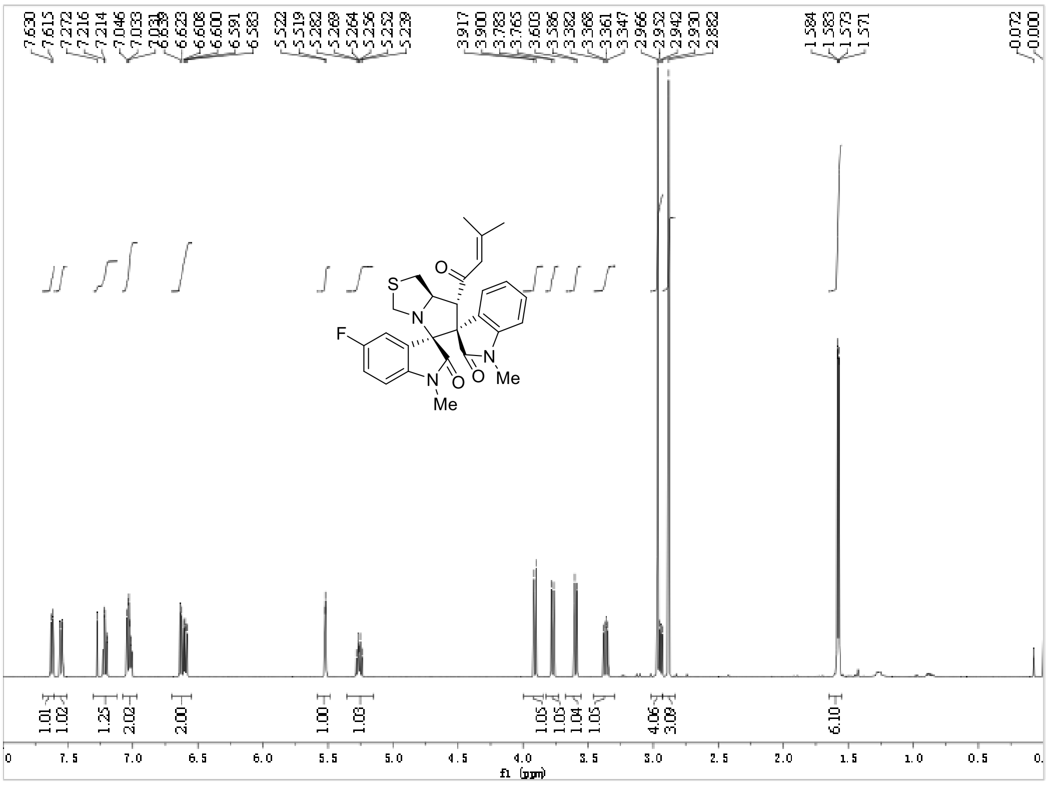

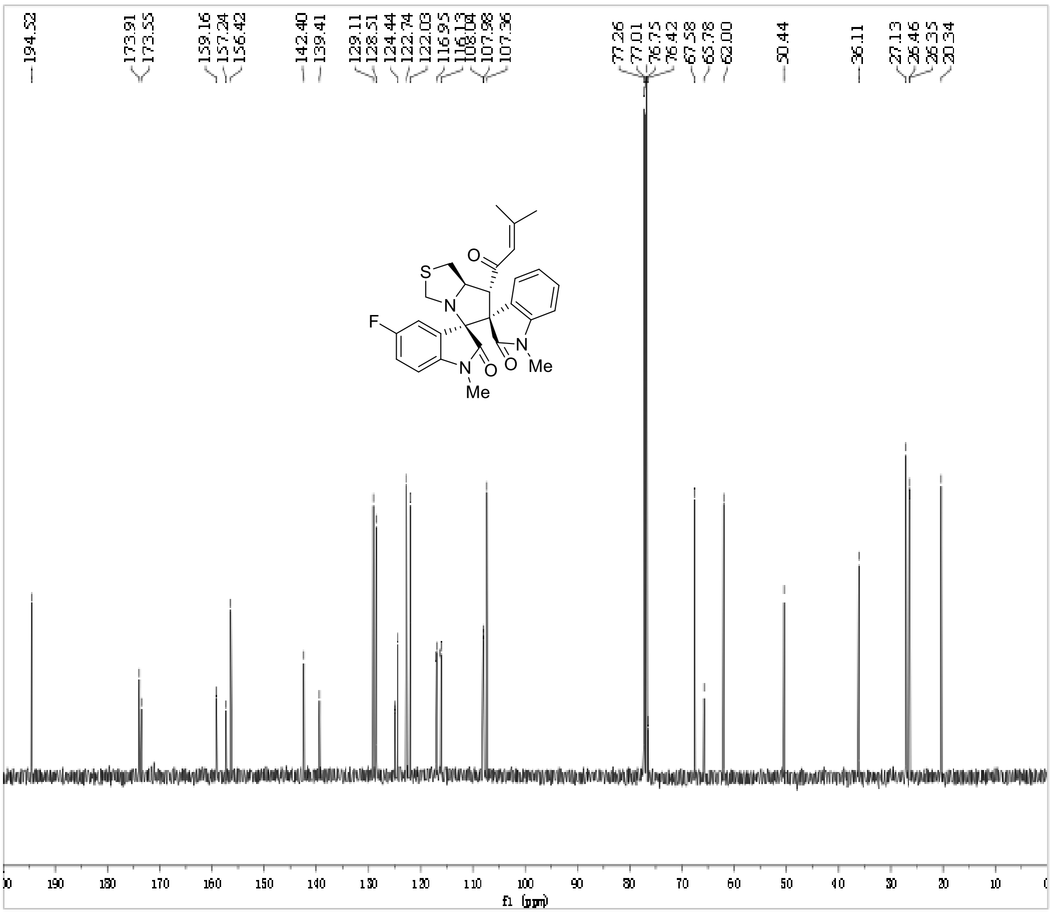

^{1}H and ^{13}C NMR of 4ma

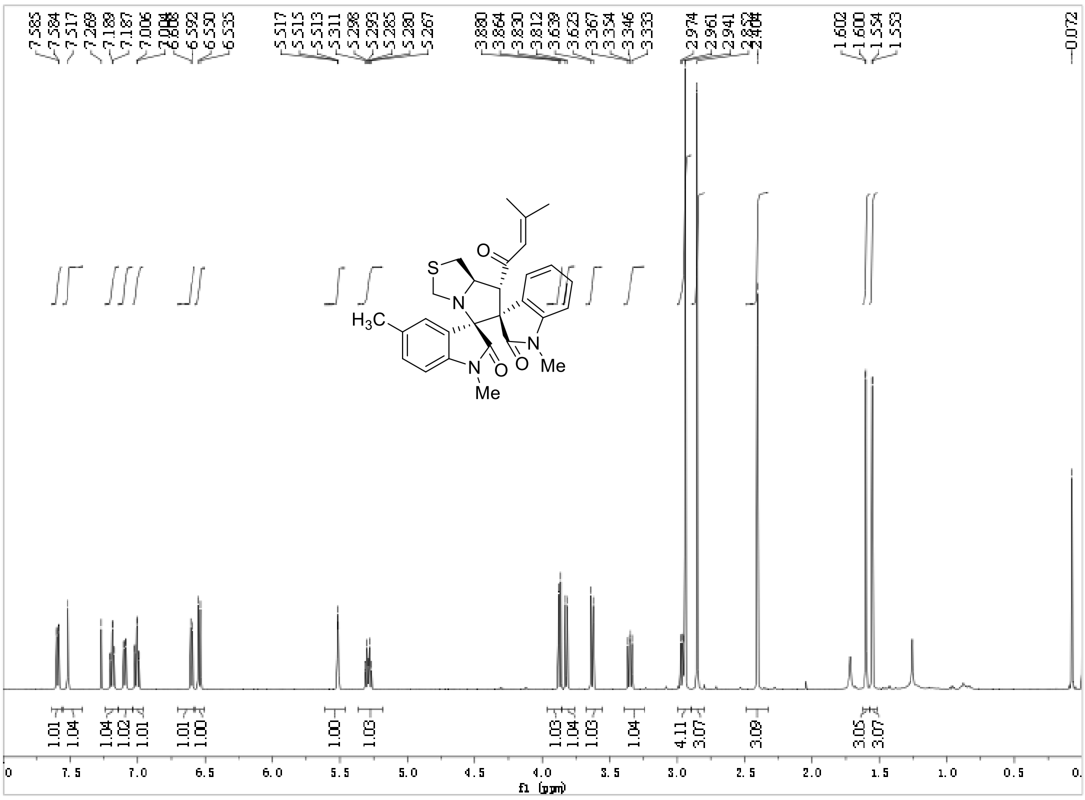

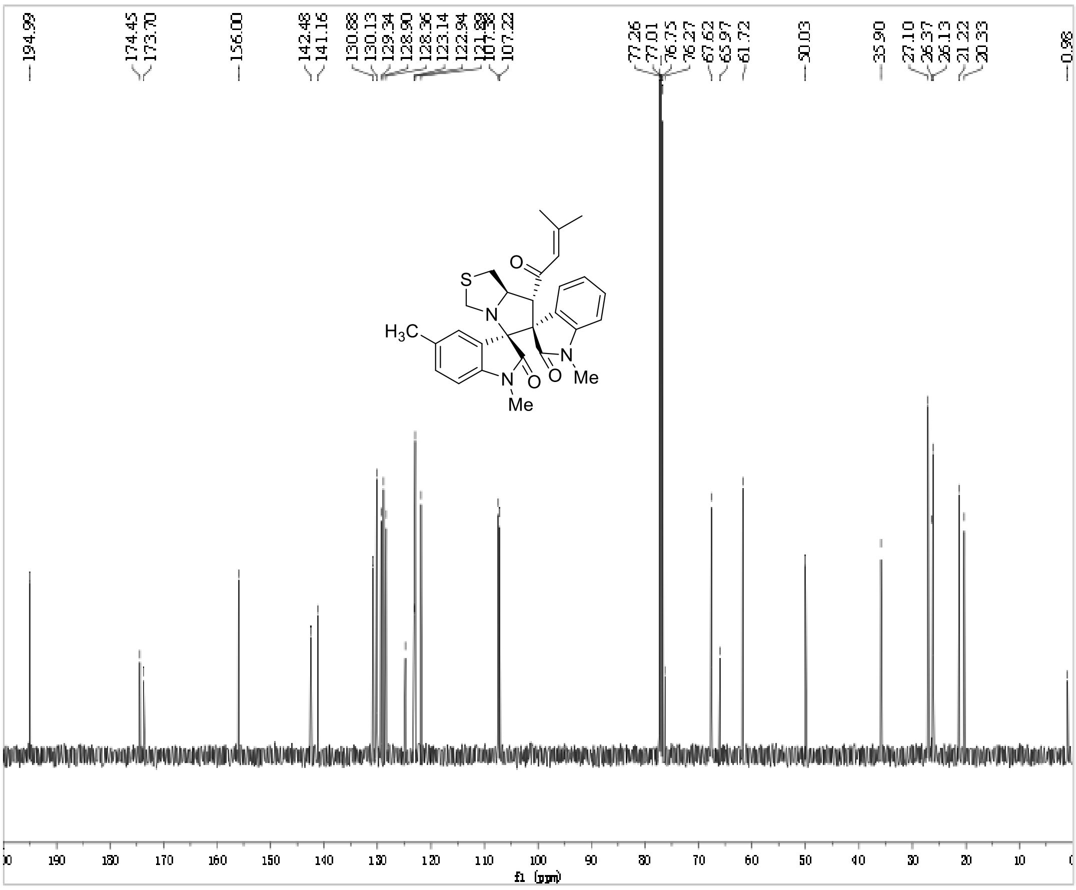

^{1}H and ^{13}C NMR of 4ag

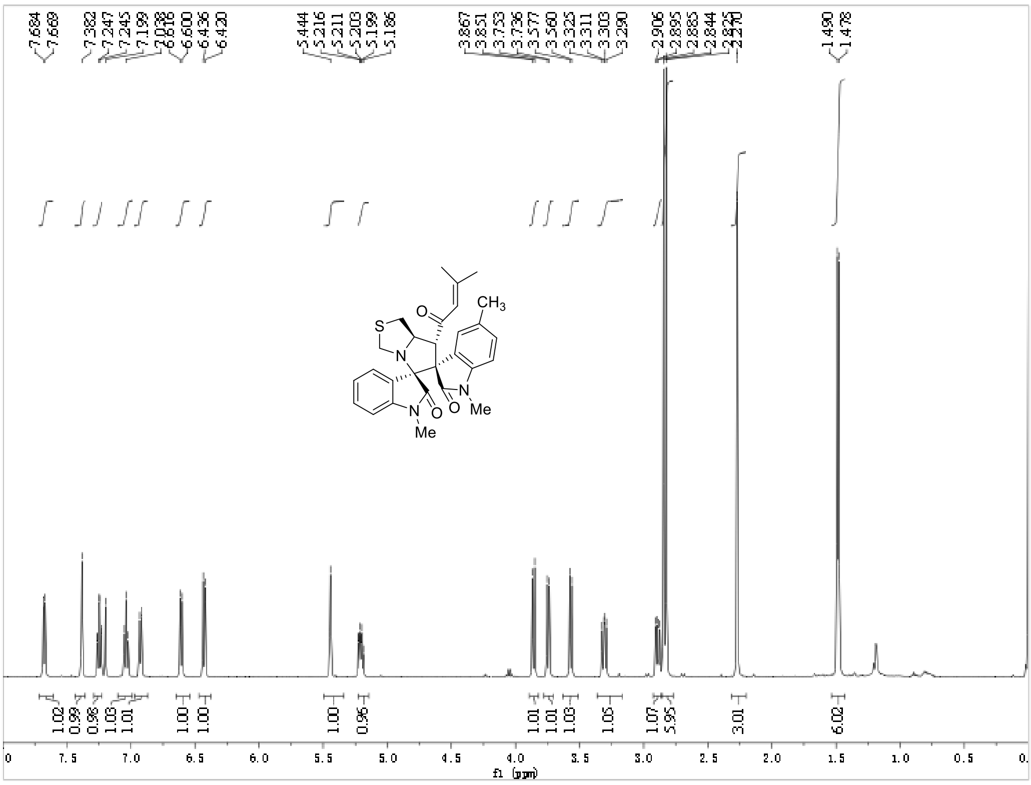
7.684
7.669
7.382
7.247
7.245
7.199
6.600
6.436
6.420
5.444
5.216
5.211
5.203
5.199
5.186
3.867
3.851
3.753
3.736
3.577
3.560
3.325
3.311
3.303
3.290
2.906
2.895
2.885
2.844
2.270
1.490
1.478
CH3
S
N
O
Me
1.02
0.99
0.98
1.03
1.01
1.00
1.00
1.00
0.95
1.01
1.01
1.03
1.05
1.07
5.95
3.01
6.02
f1 (ppm)

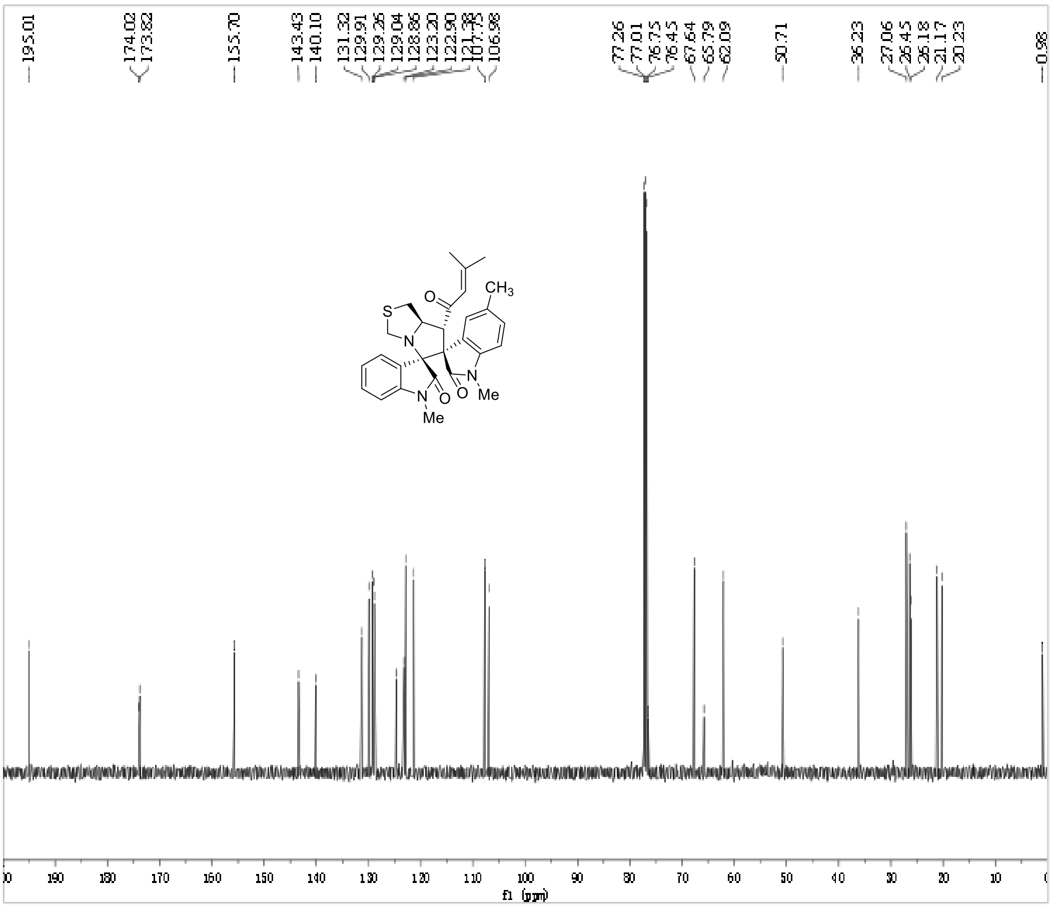
195.01
174.02
173.82
155.70
143.43
140.10
131.32
129.91
129.26
129.04
128.86
123.20
122.90
106.98
77.26
77.01
76.75
67.64
65.79
62.09
50.71
36.23
27.06
26.45
26.18
21.17
20.23
0.98
CH3
S
N
O
Me
f1 (ppm)

^{1}H and ^{13}C NMR of 4mh

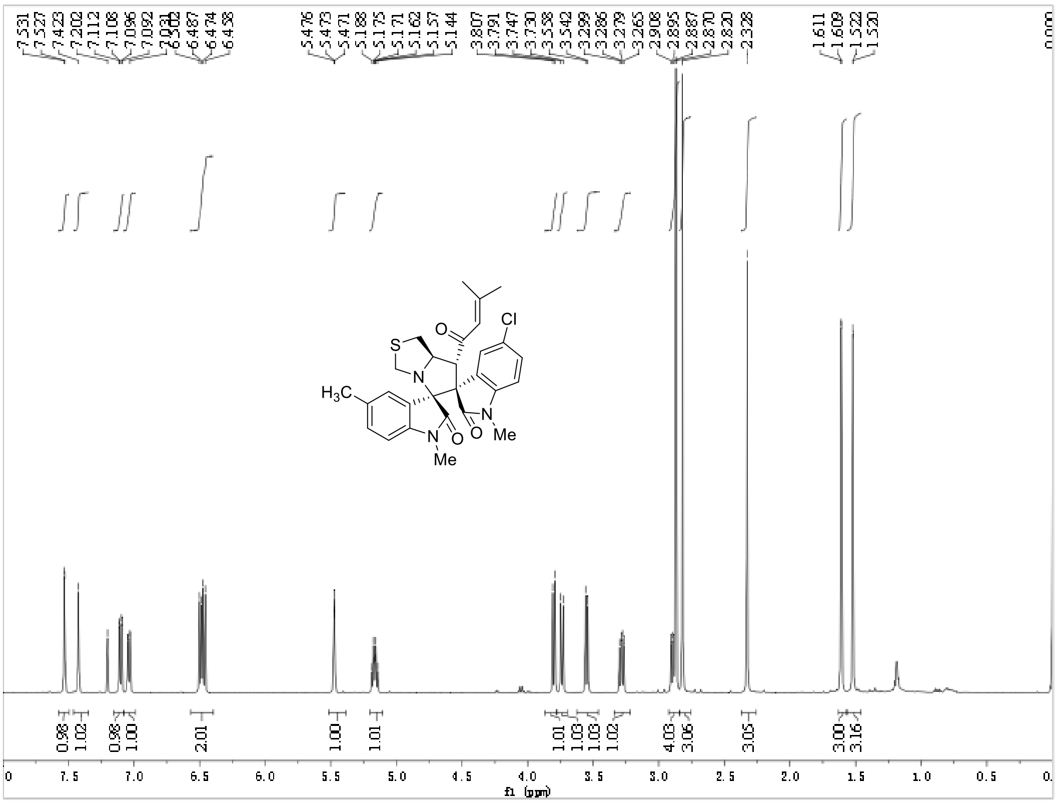

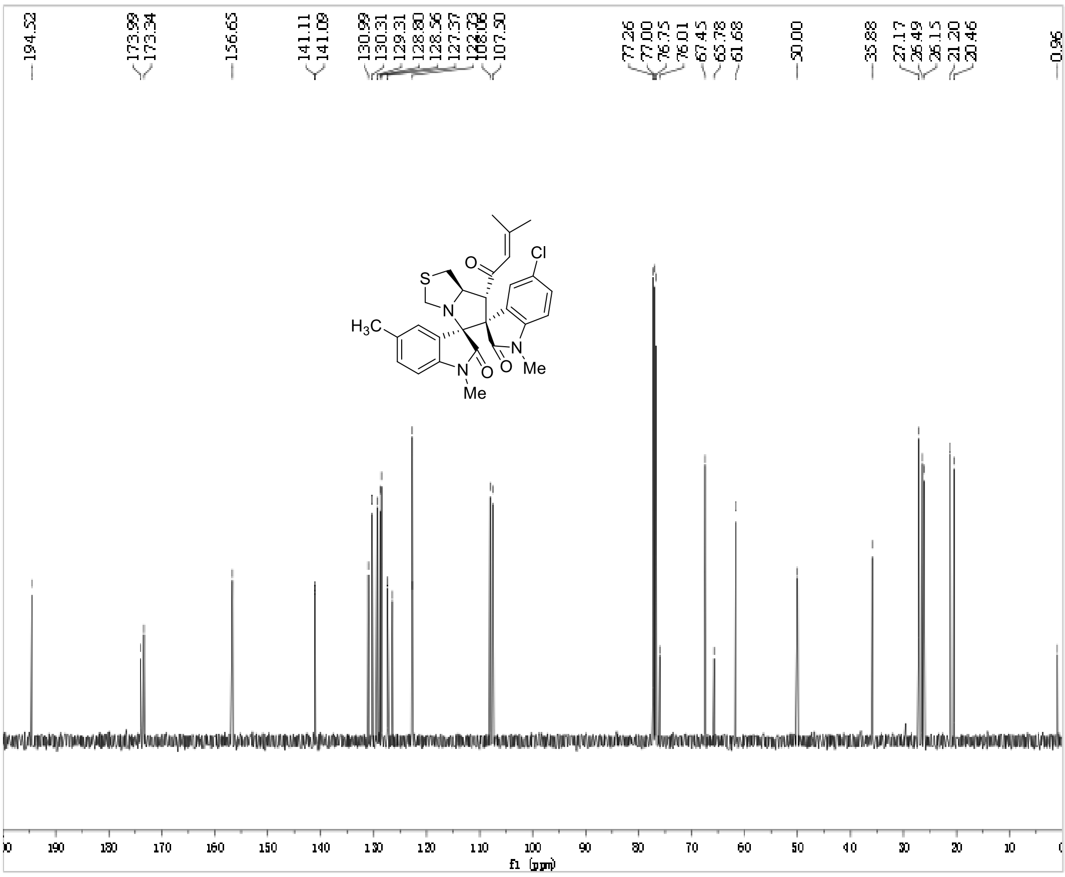

^{1}H and ^{13}C NMR of 4ng

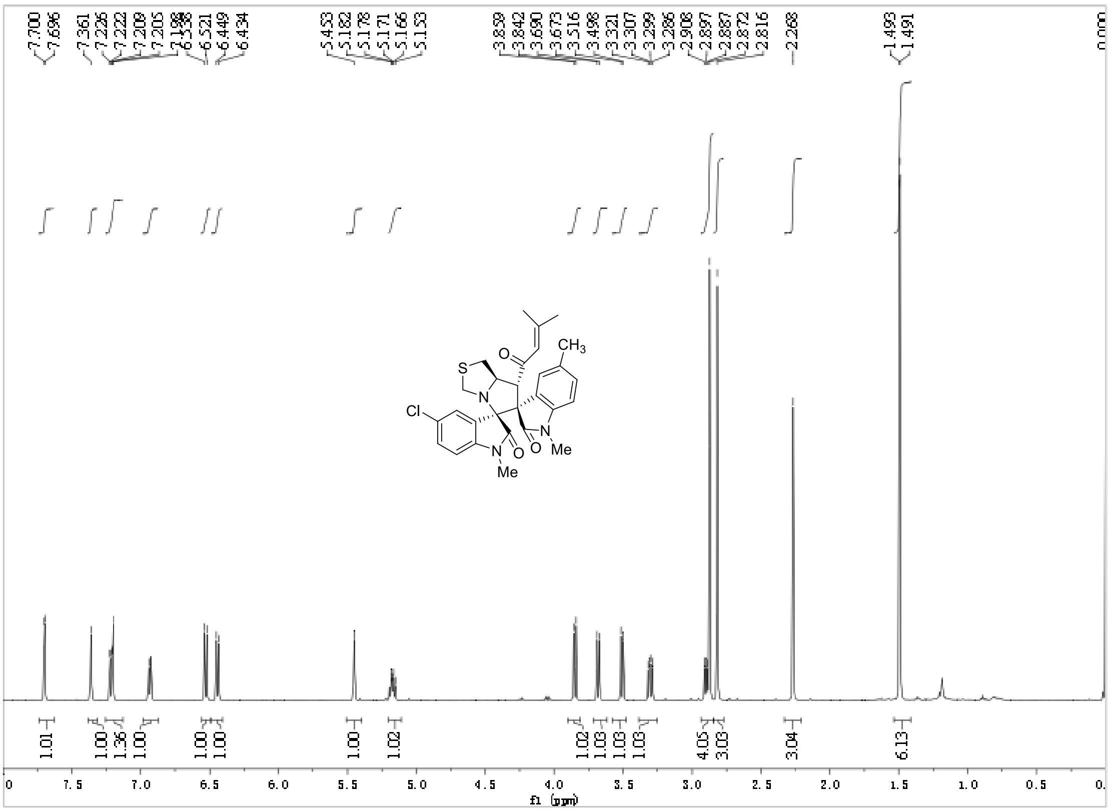

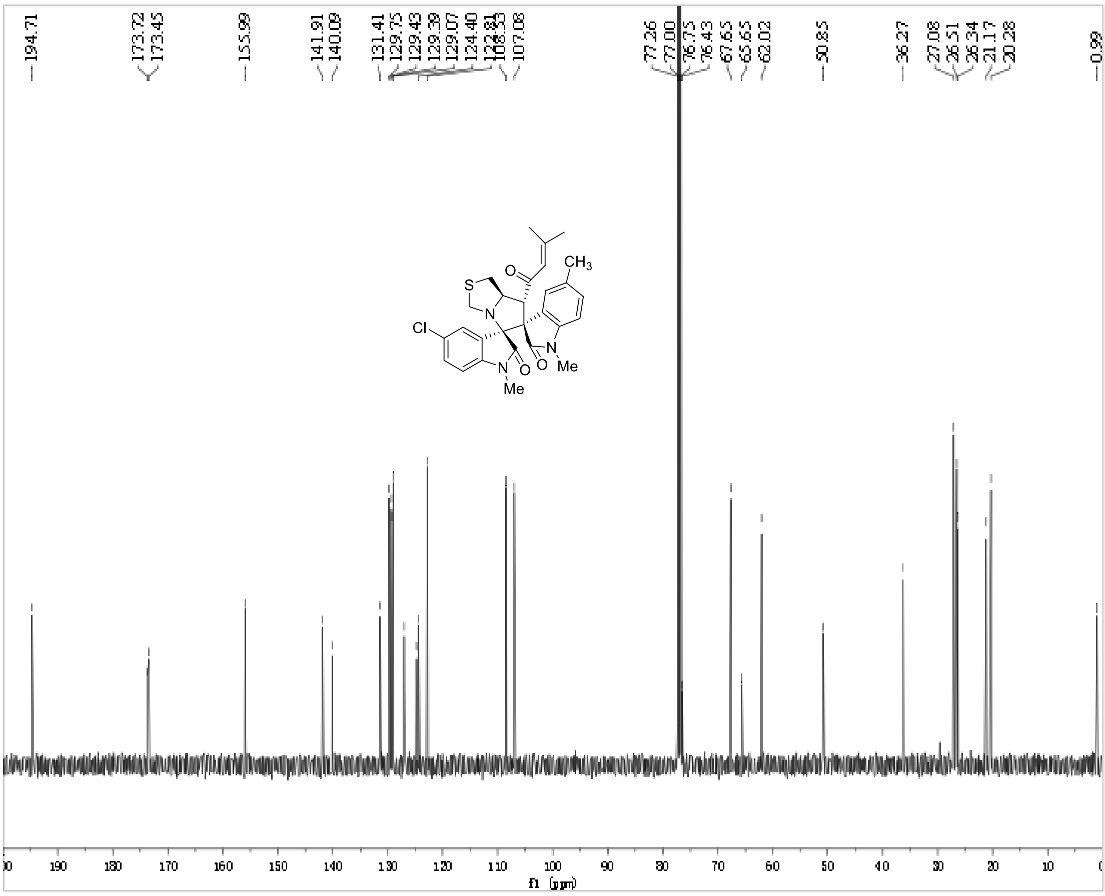

^{1}H and ^{13}C NMR of 4nh

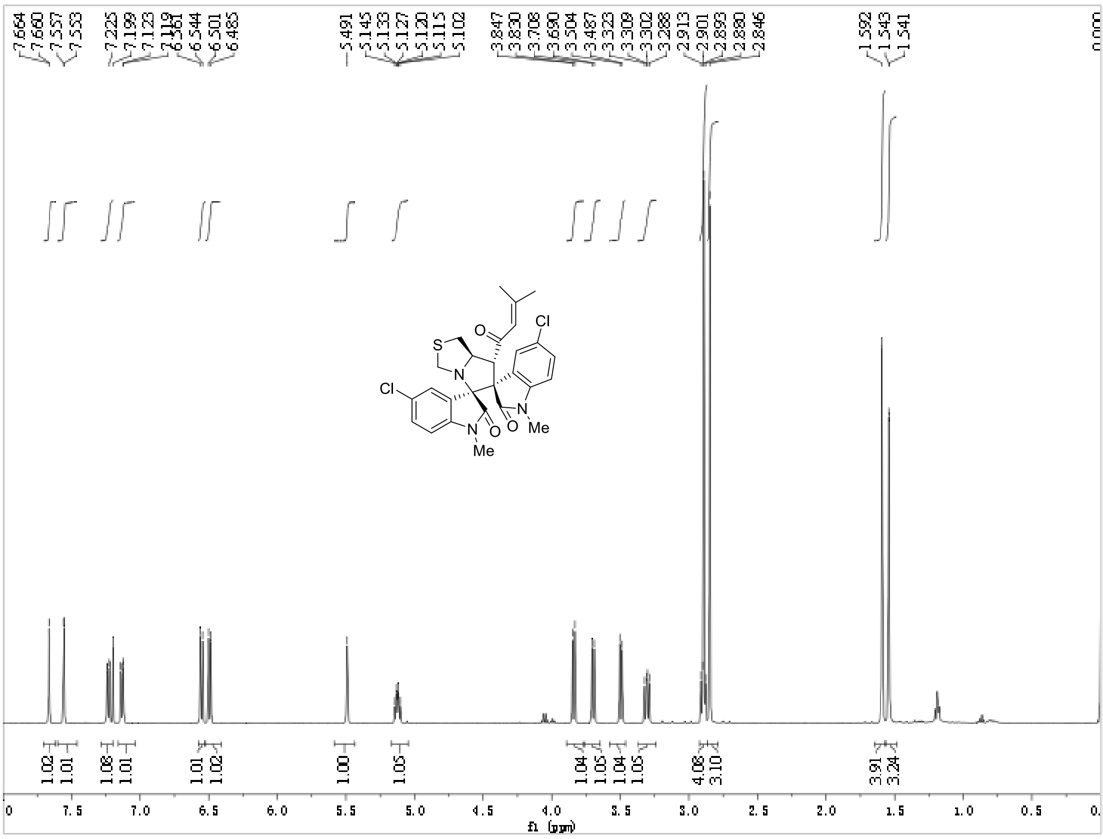

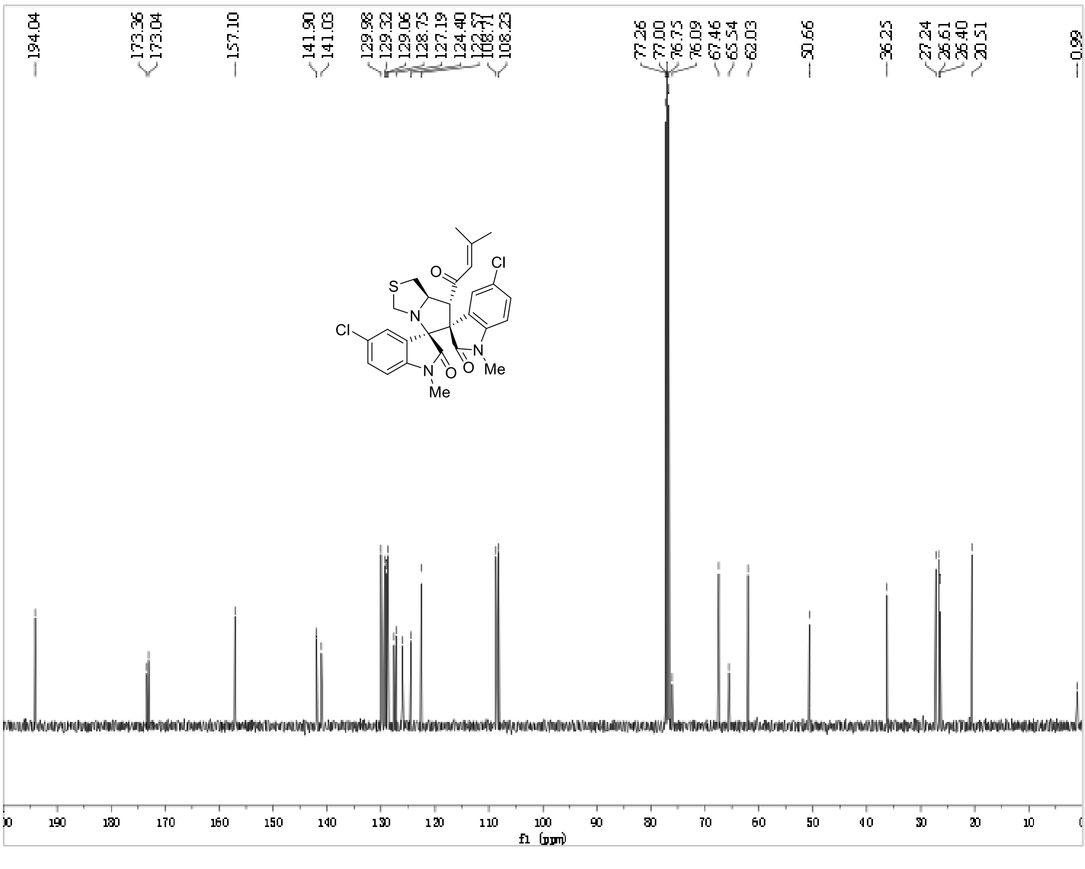

^{1}H and ^{13}C NMR of 5aa

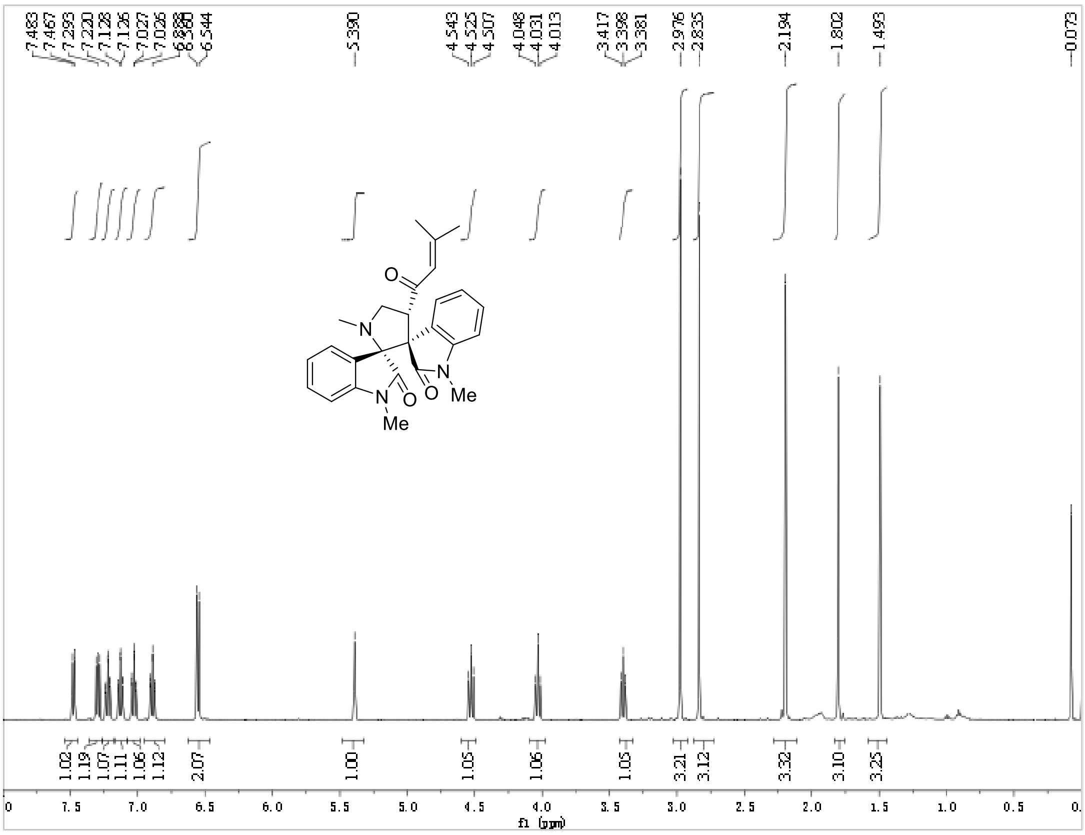

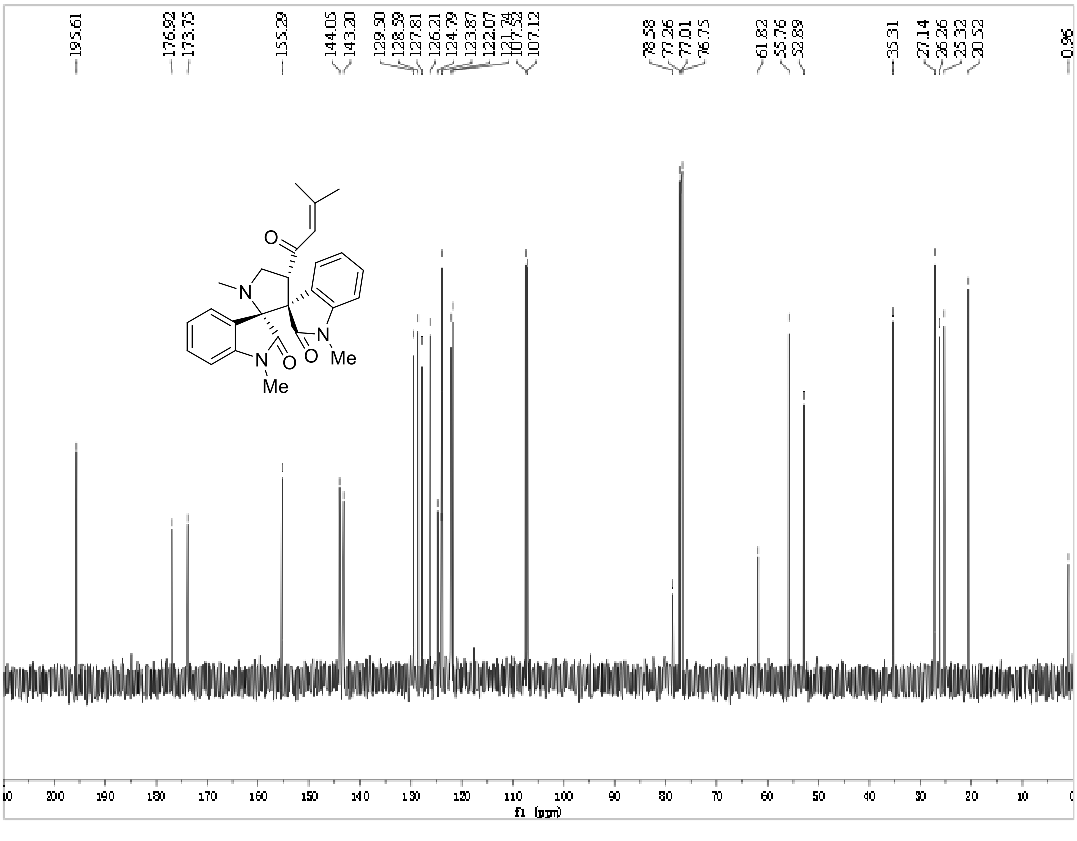

^{1}H and ^{13}C NMR of 5ba

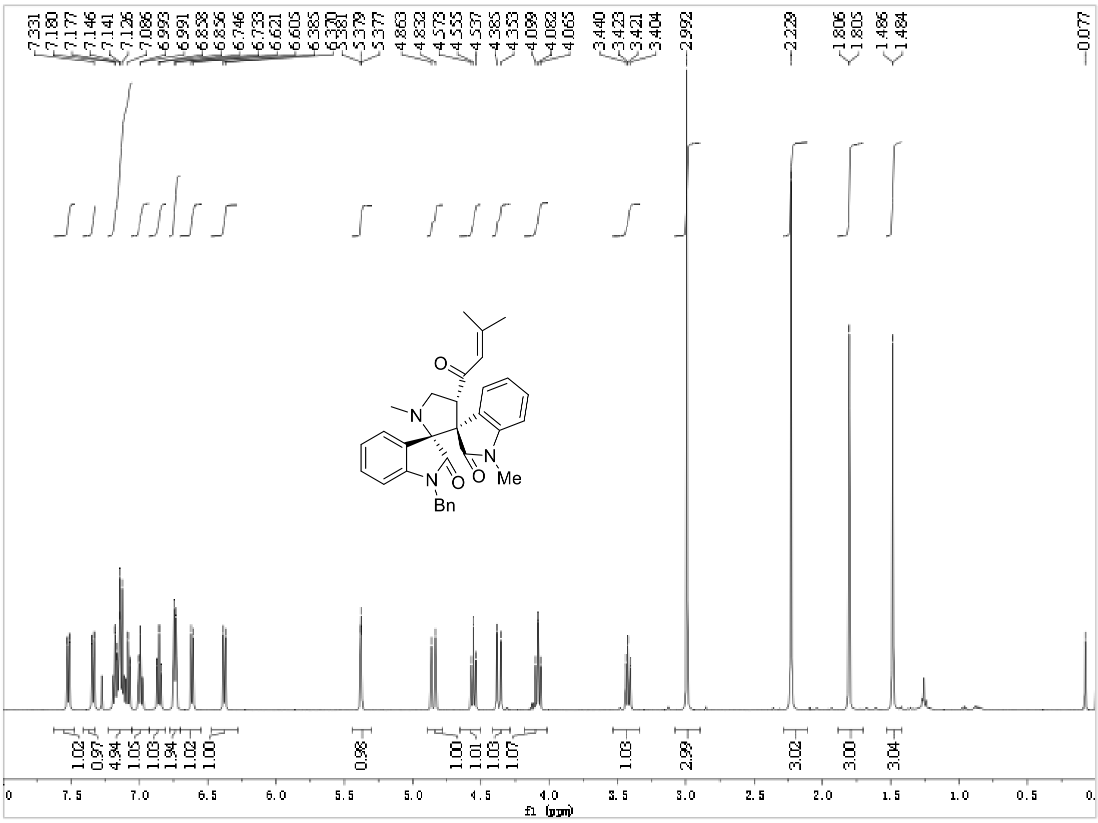

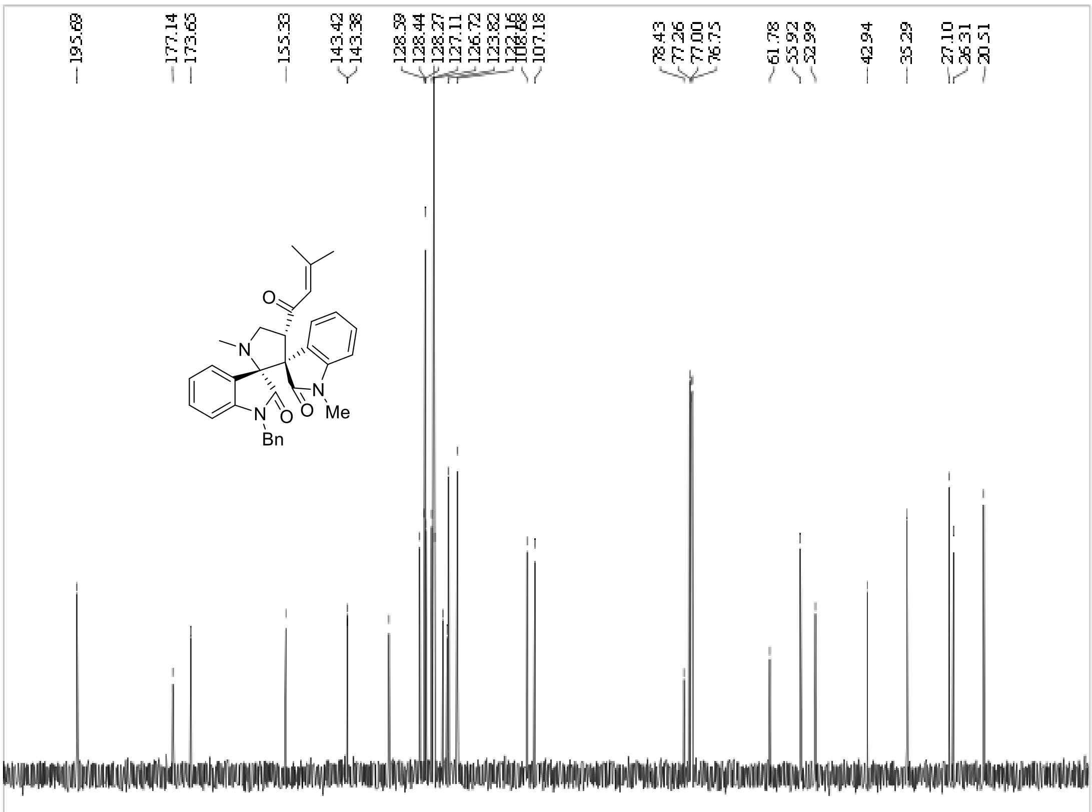

^{1}H and ^{13}C NMR of 5ca

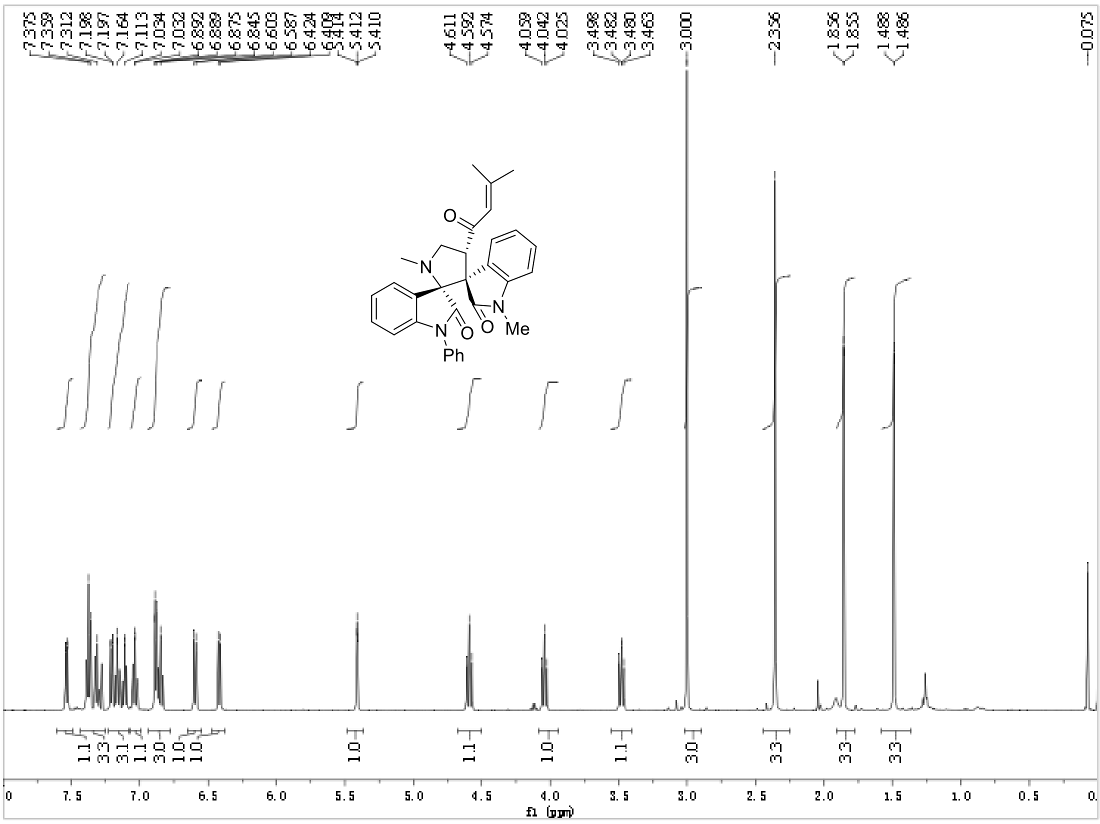

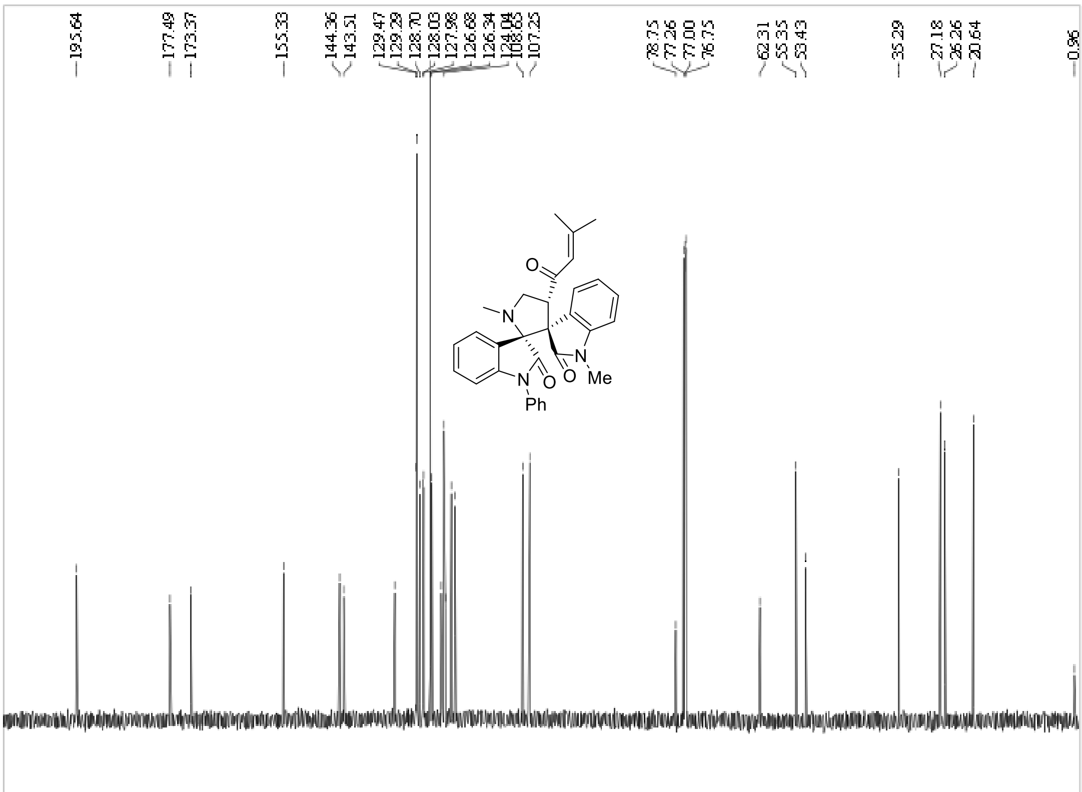

^{1}H and ^{13}C NMR of 5da

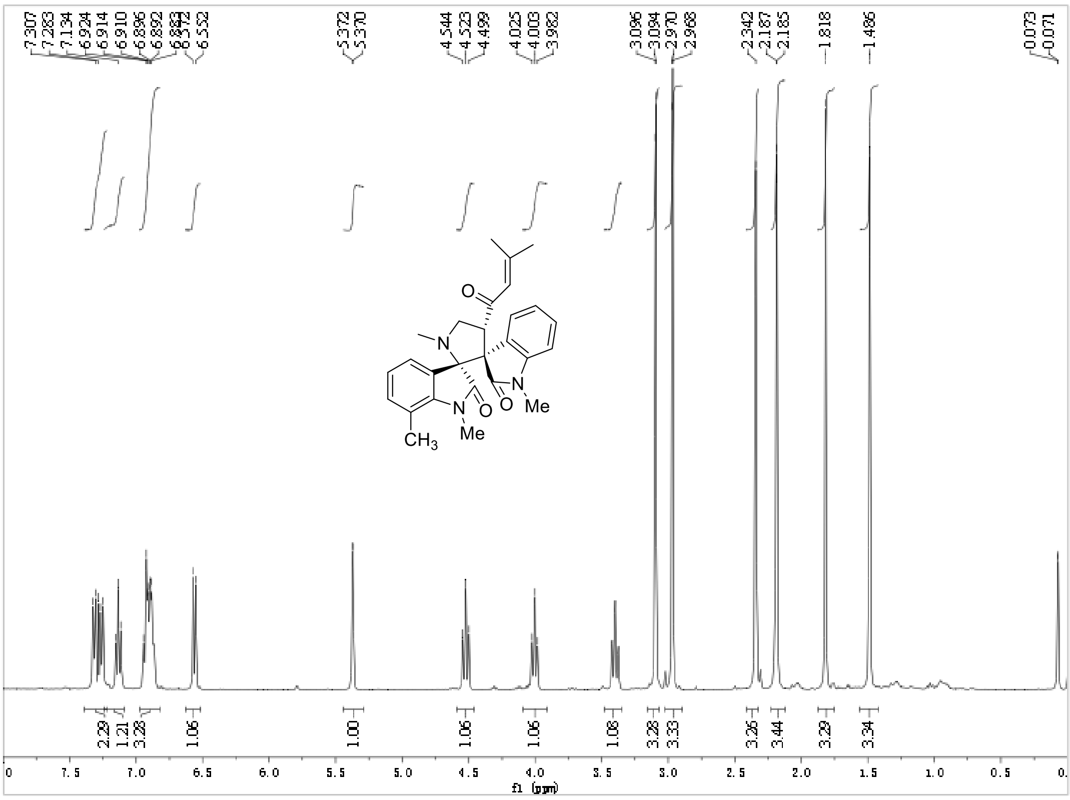

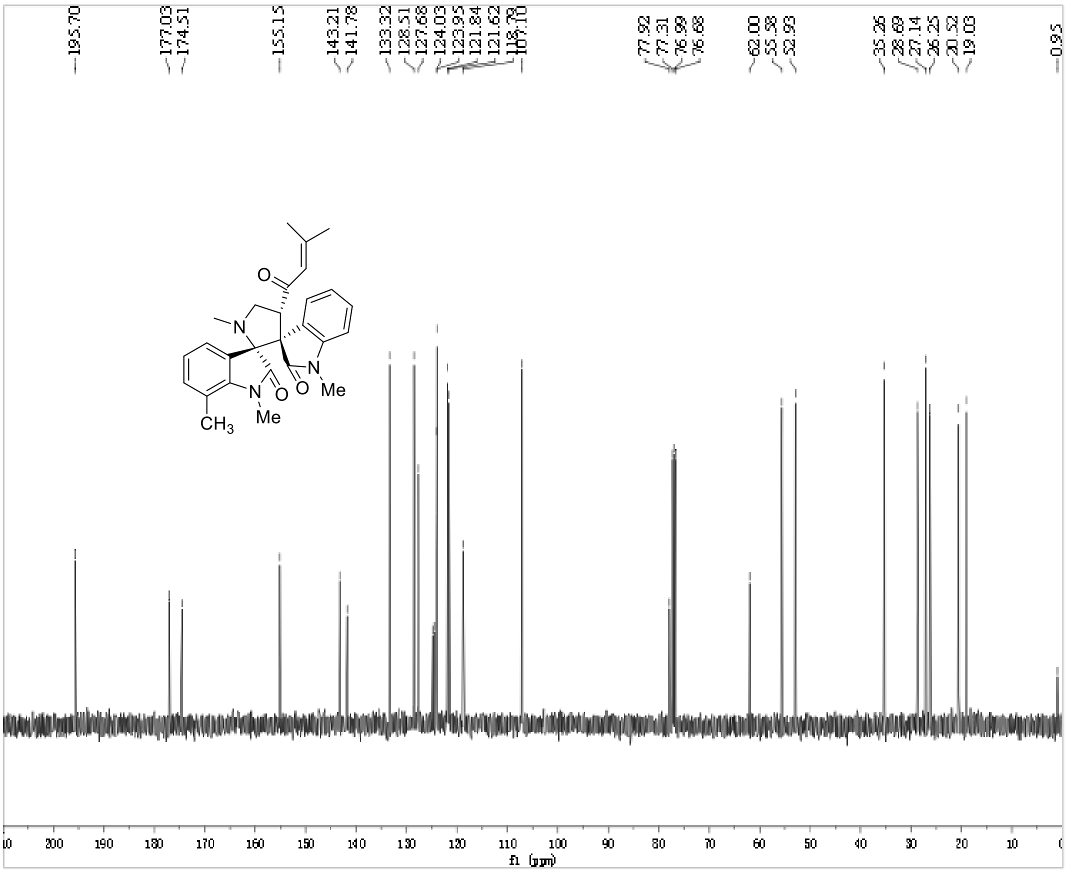

^{1}H and ^{13}C NMR of 5ea

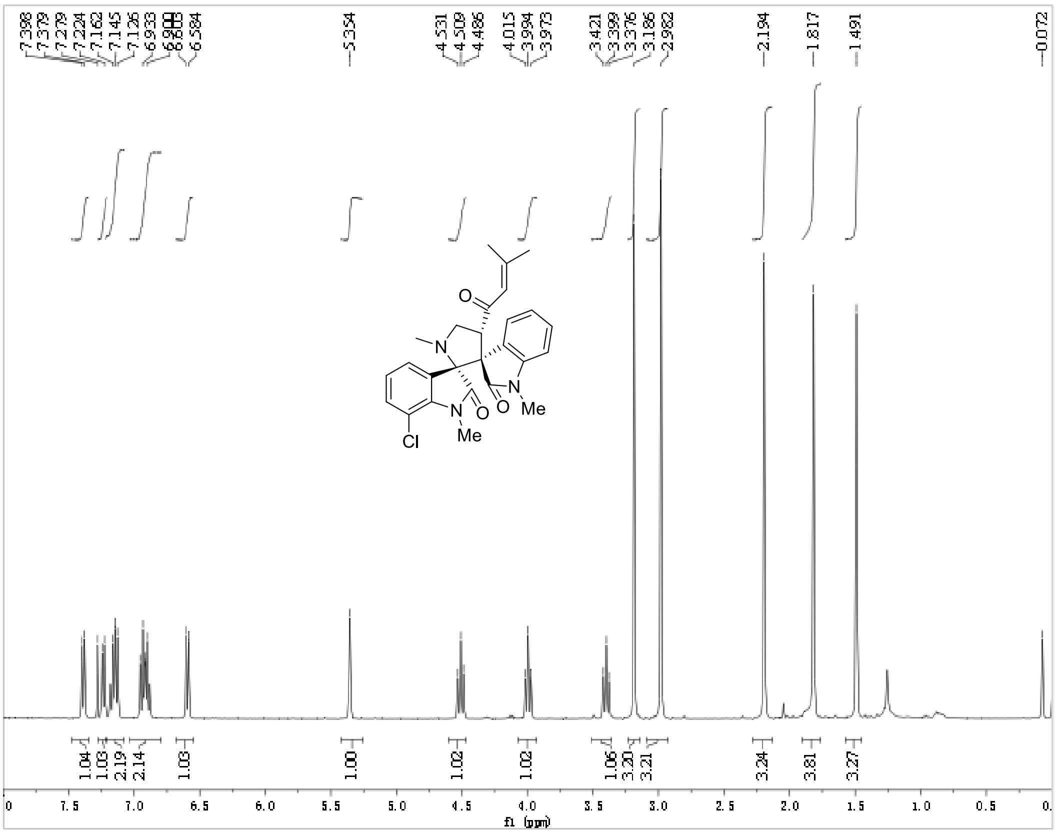

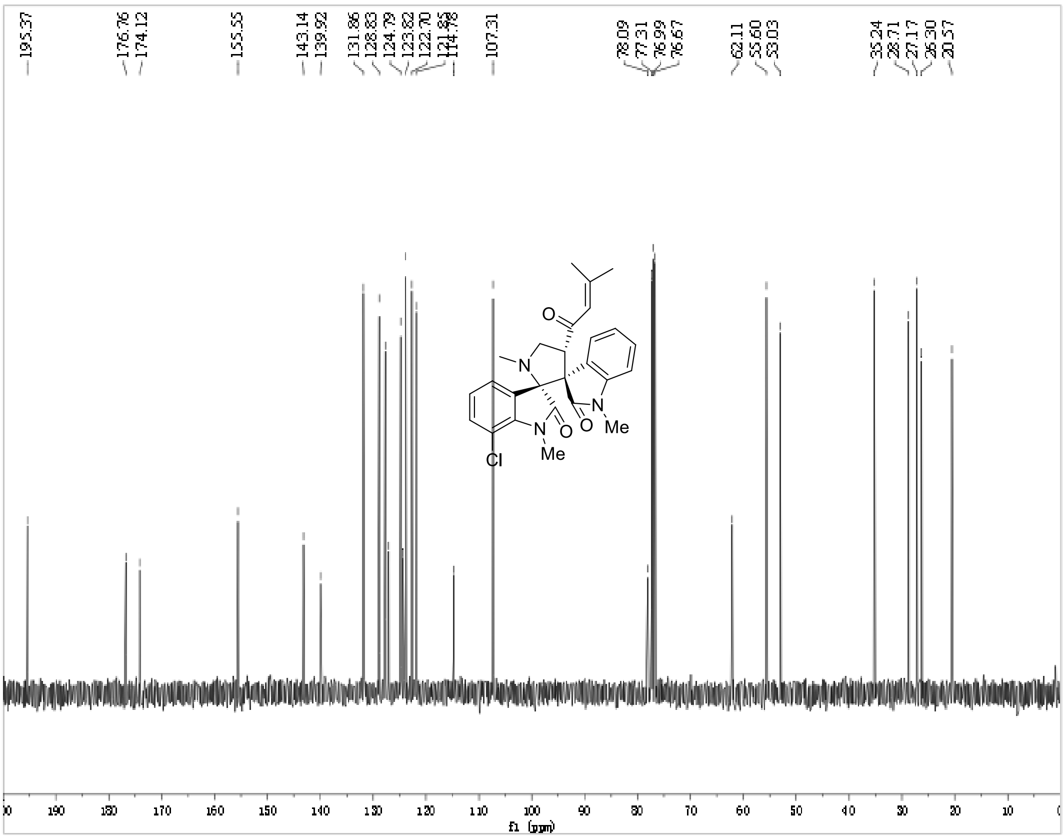

^{1}H and ^{13}C NMR of 5fa

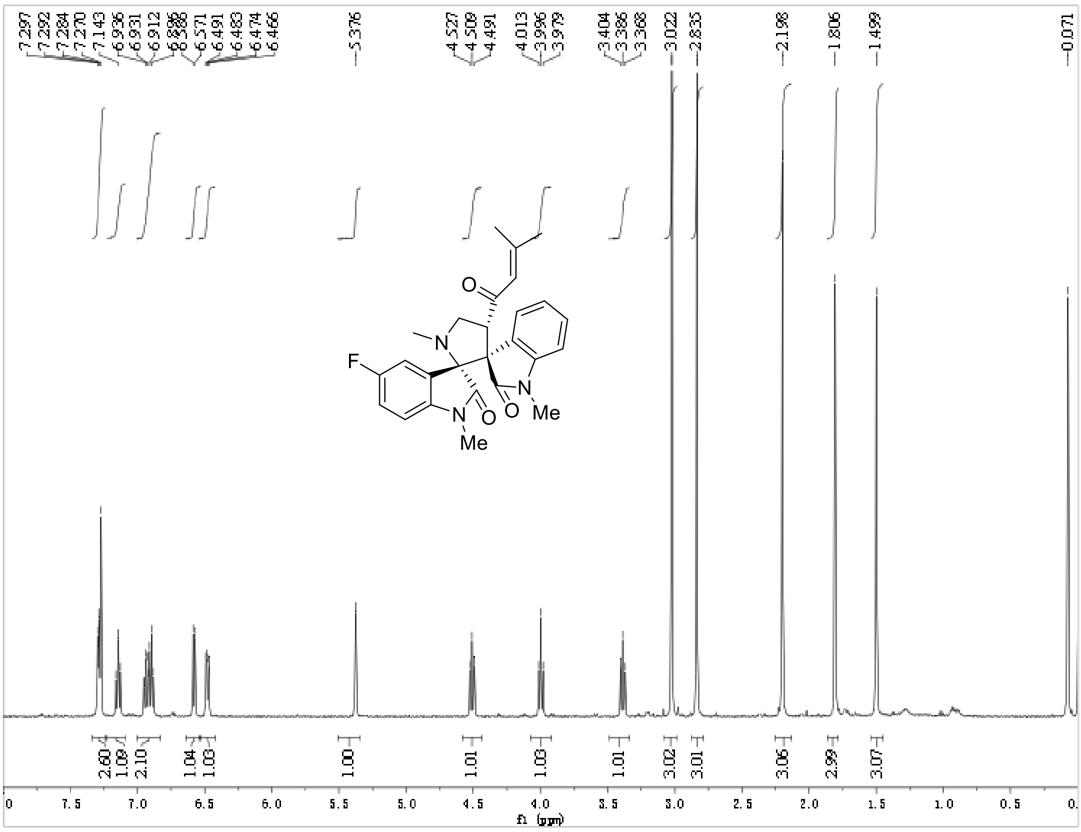

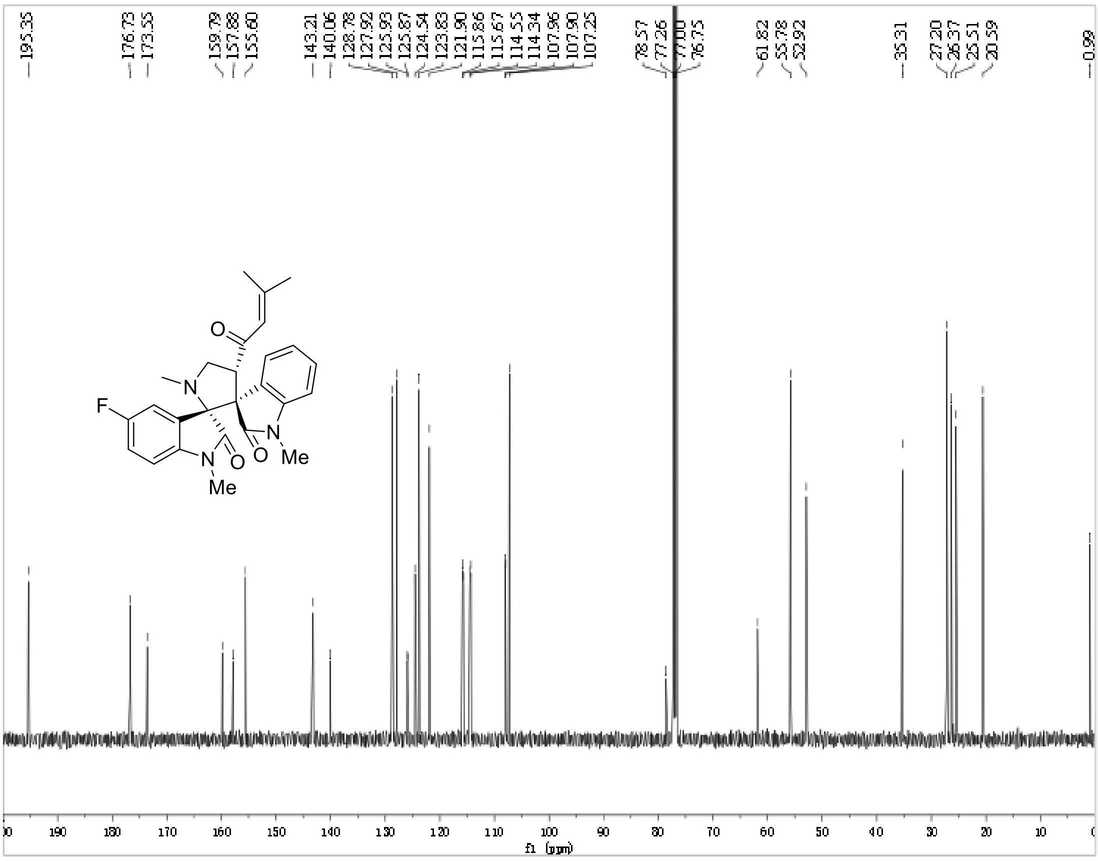

^{1}H and ^{13}C NMR of 5ga

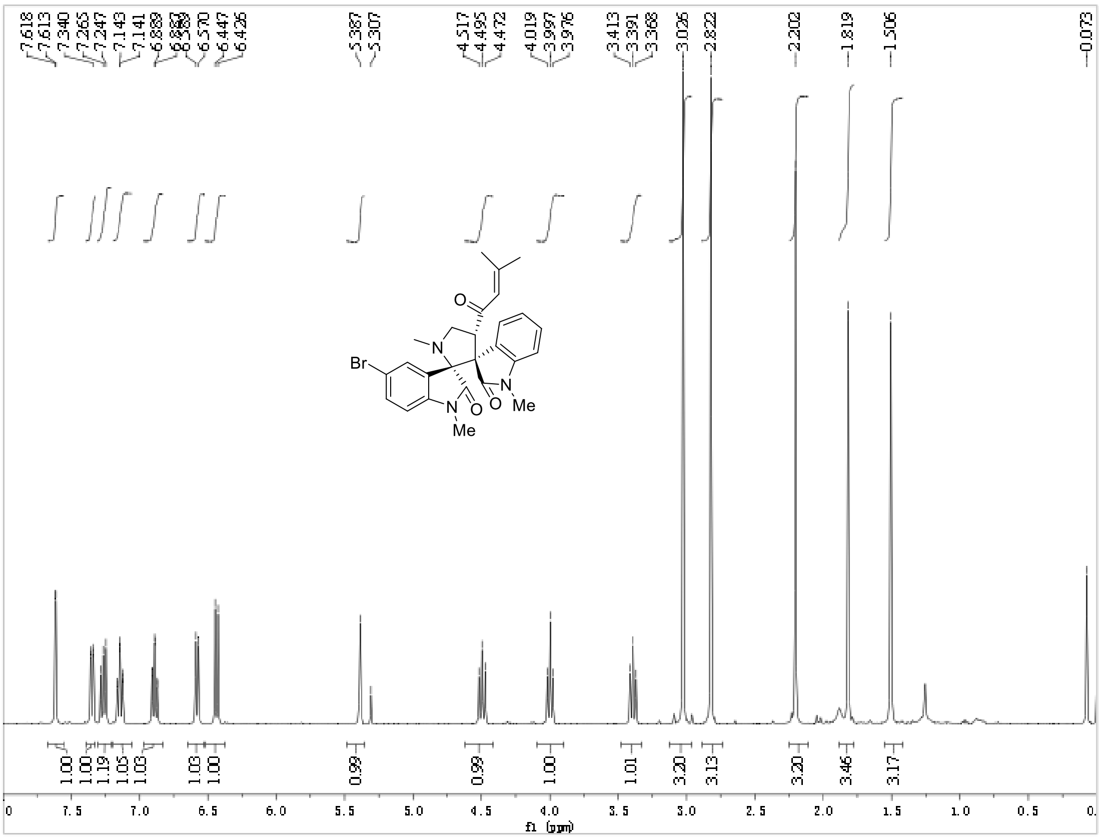

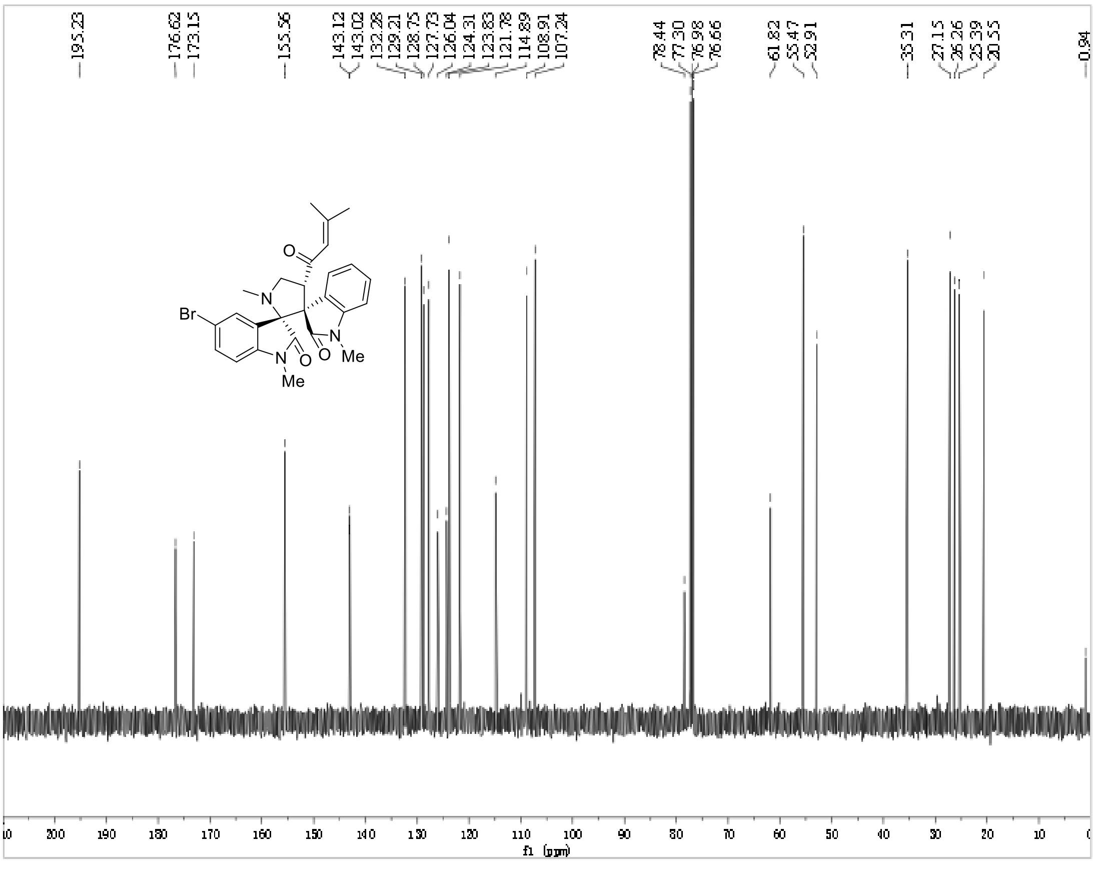

^{1}H and ^{13}C NMR of 5ia

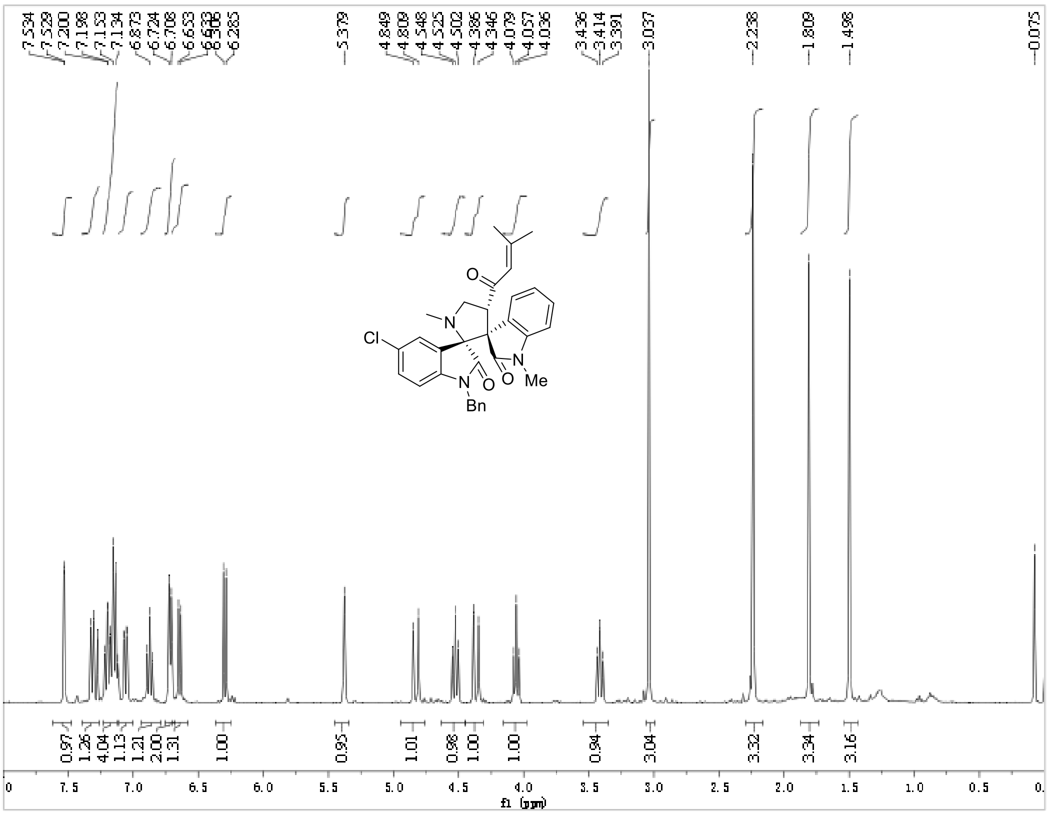

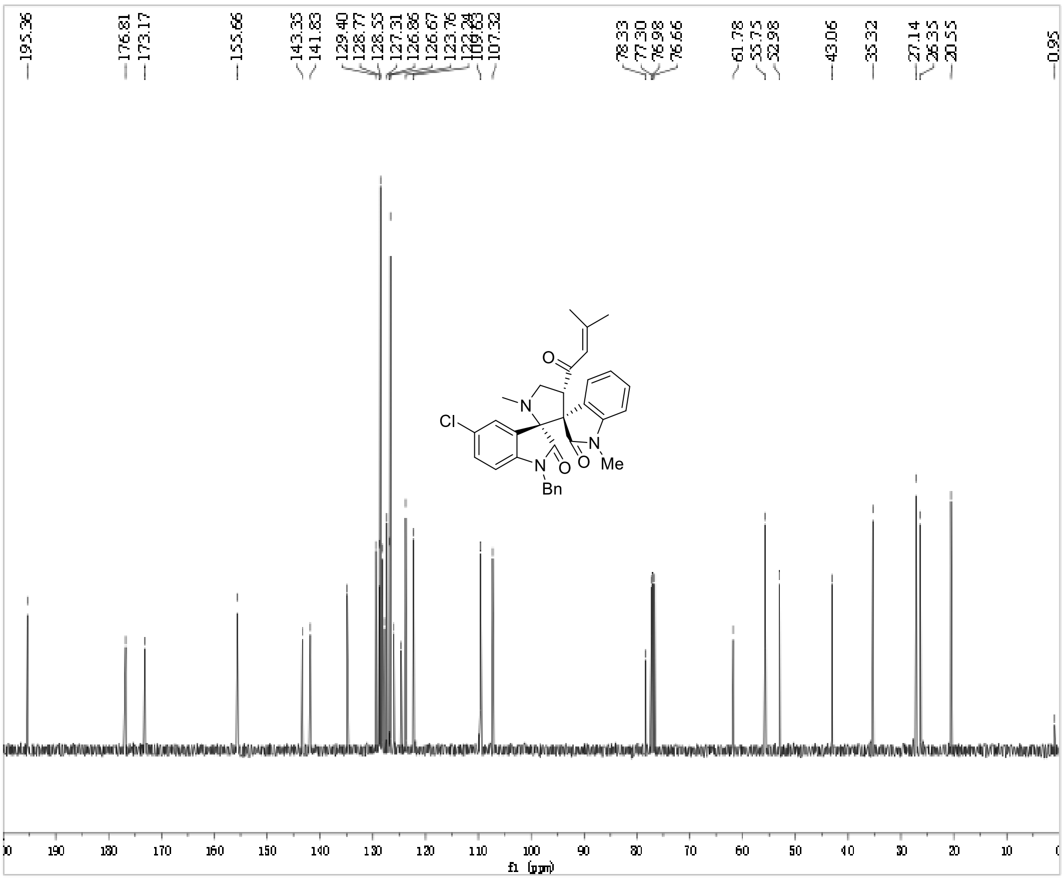

^{1}H and ^{13}C NMR of 5ja

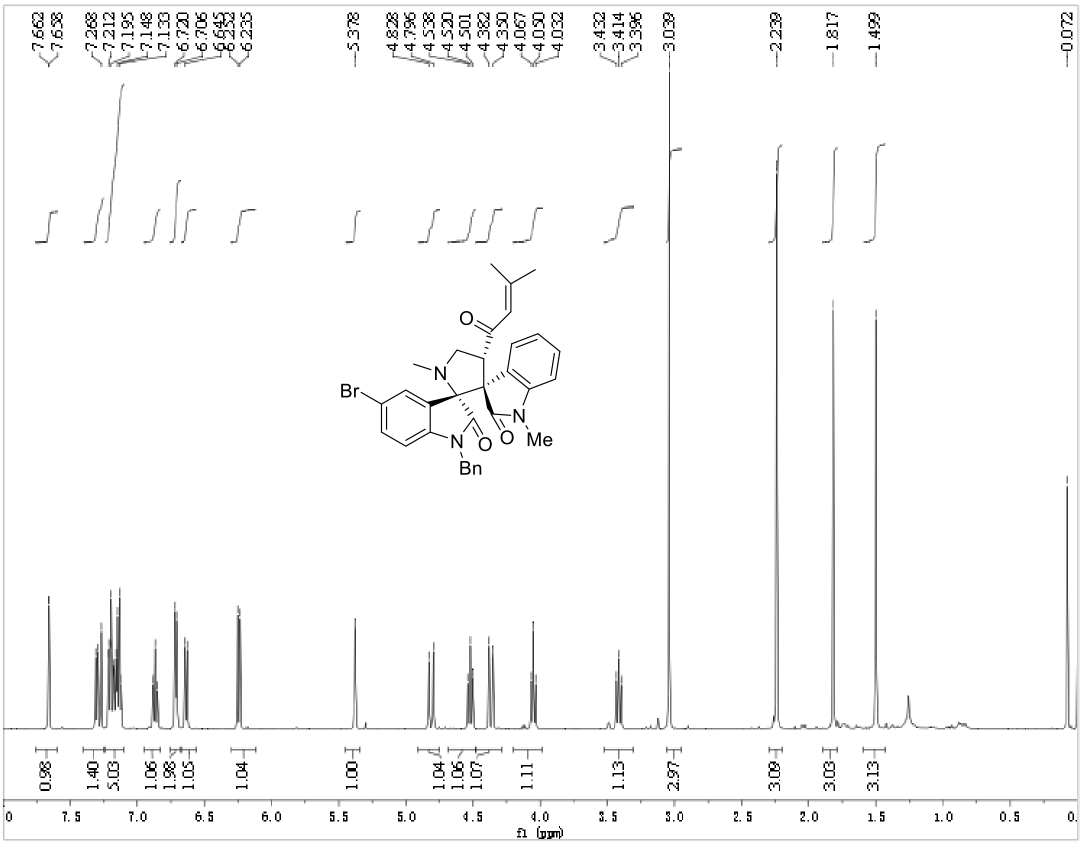

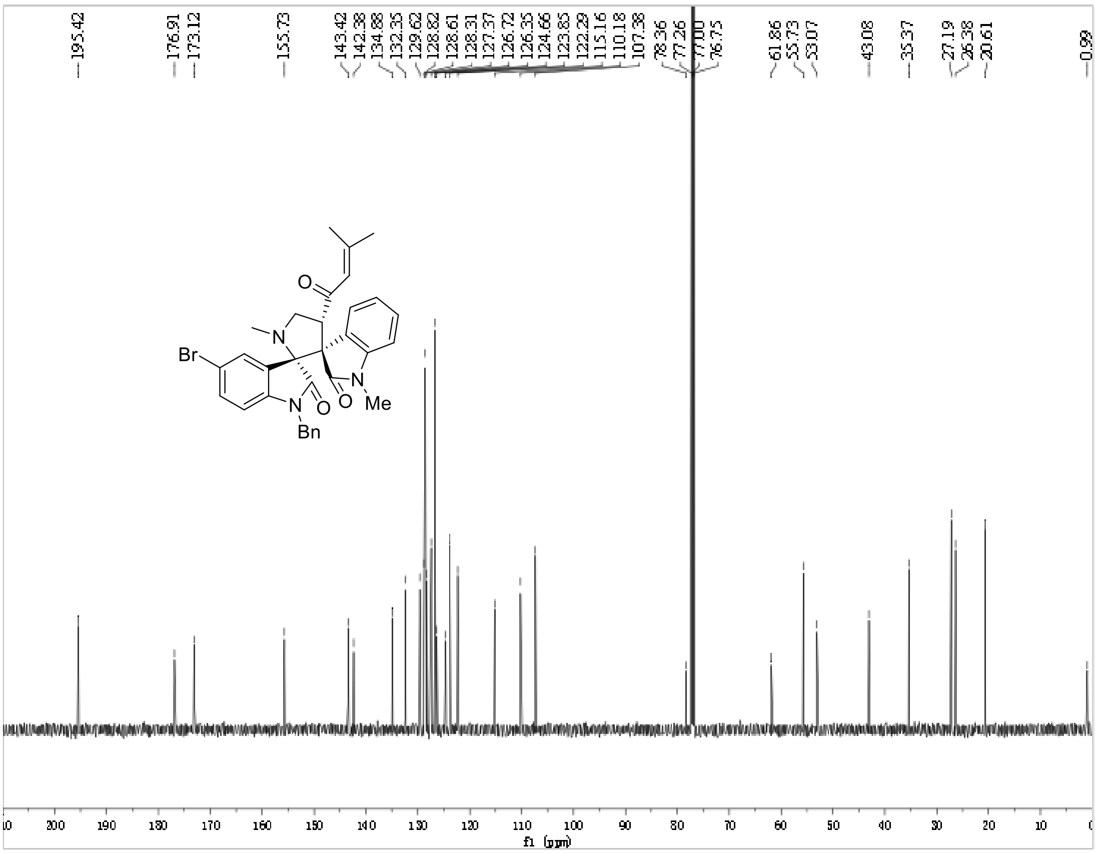

^{1}H and ^{13}C NMR of 5ma

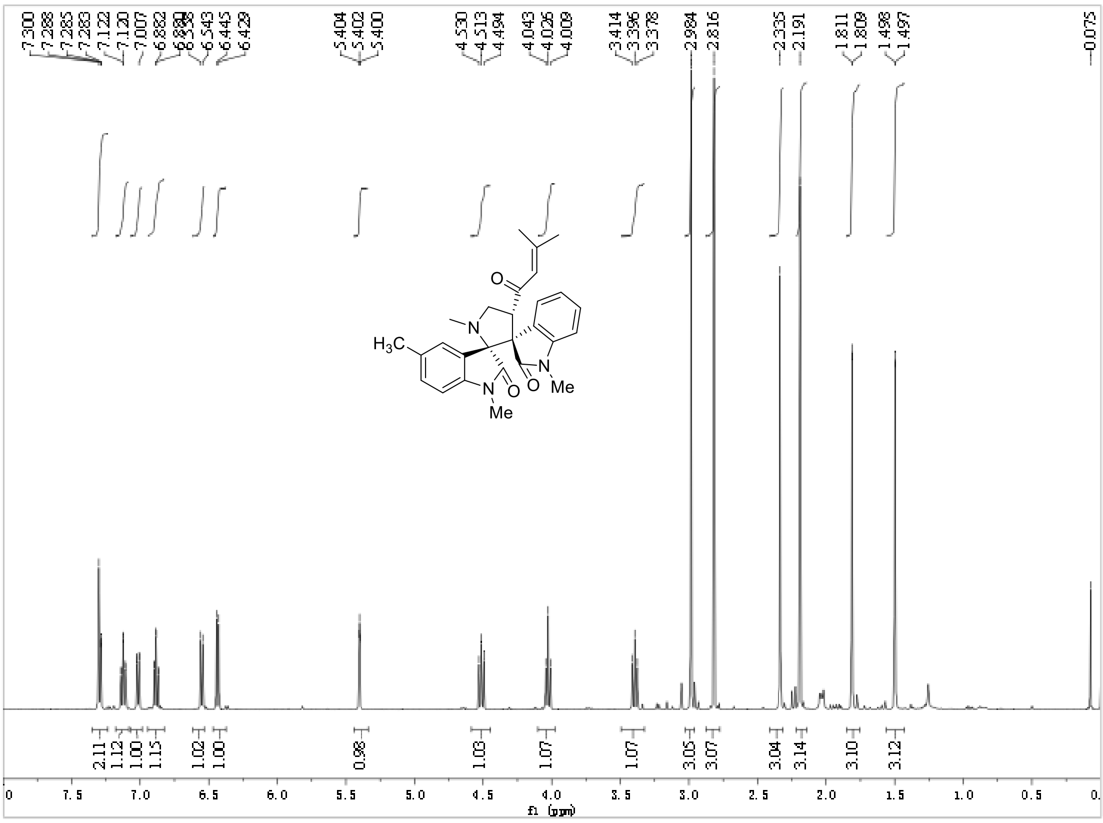

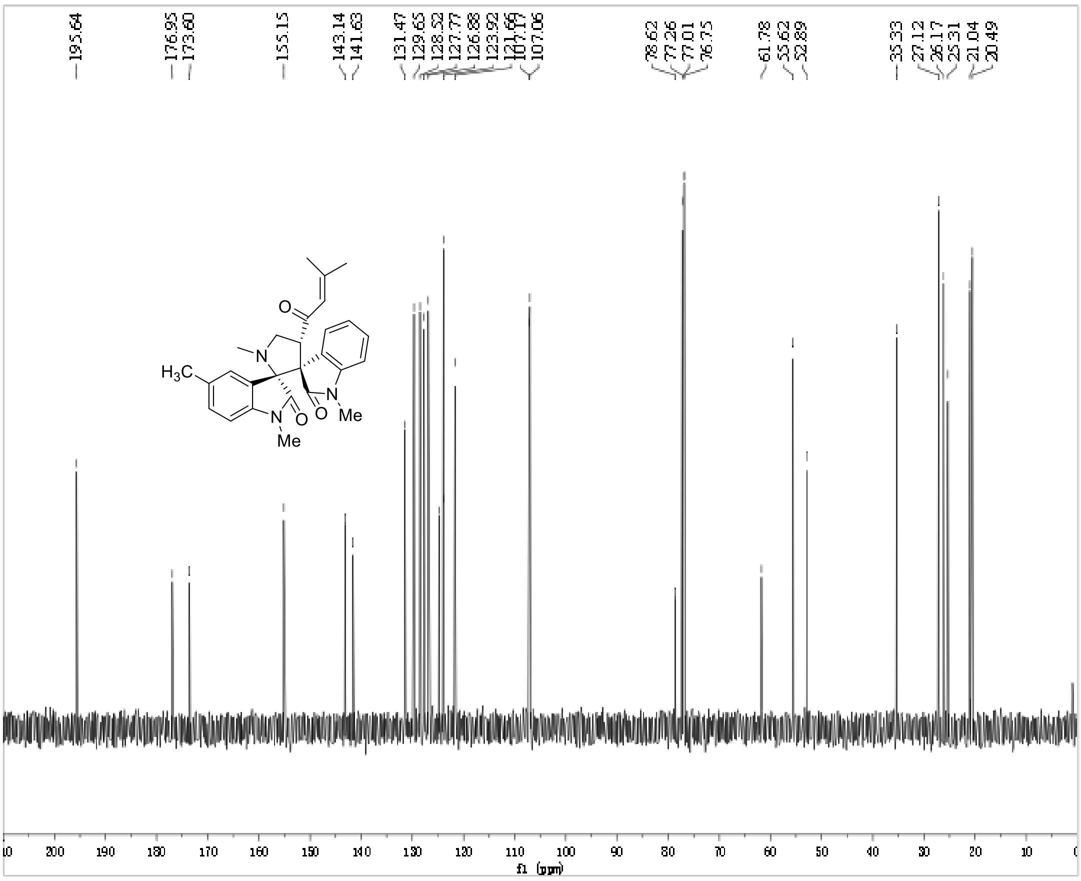

^{1}H and ^{13}C NMR of 5na

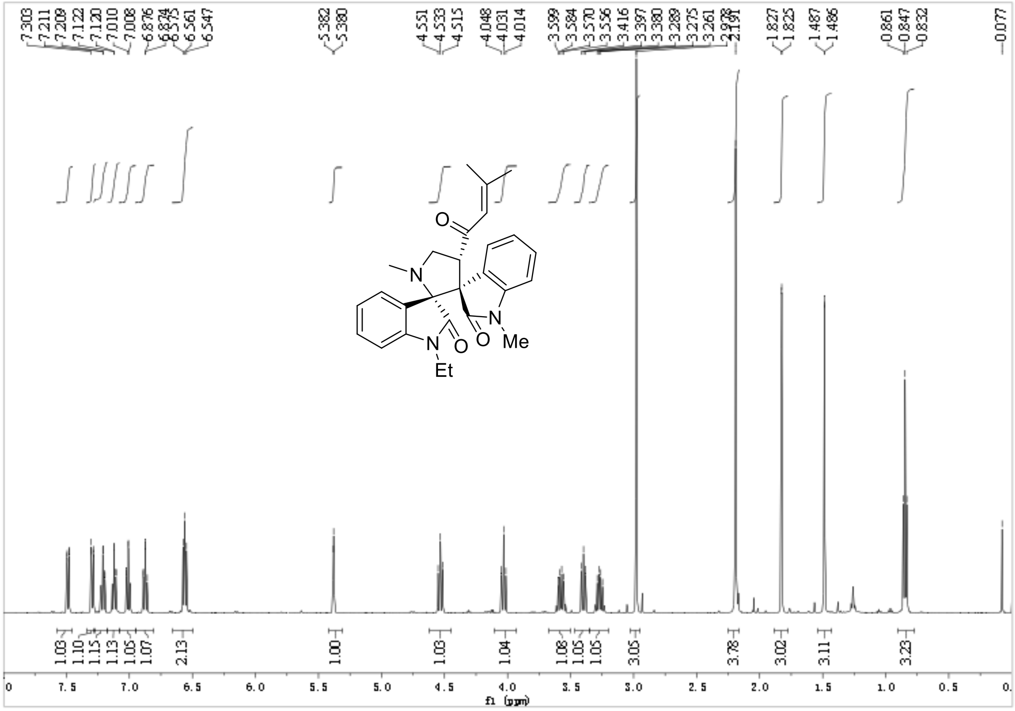

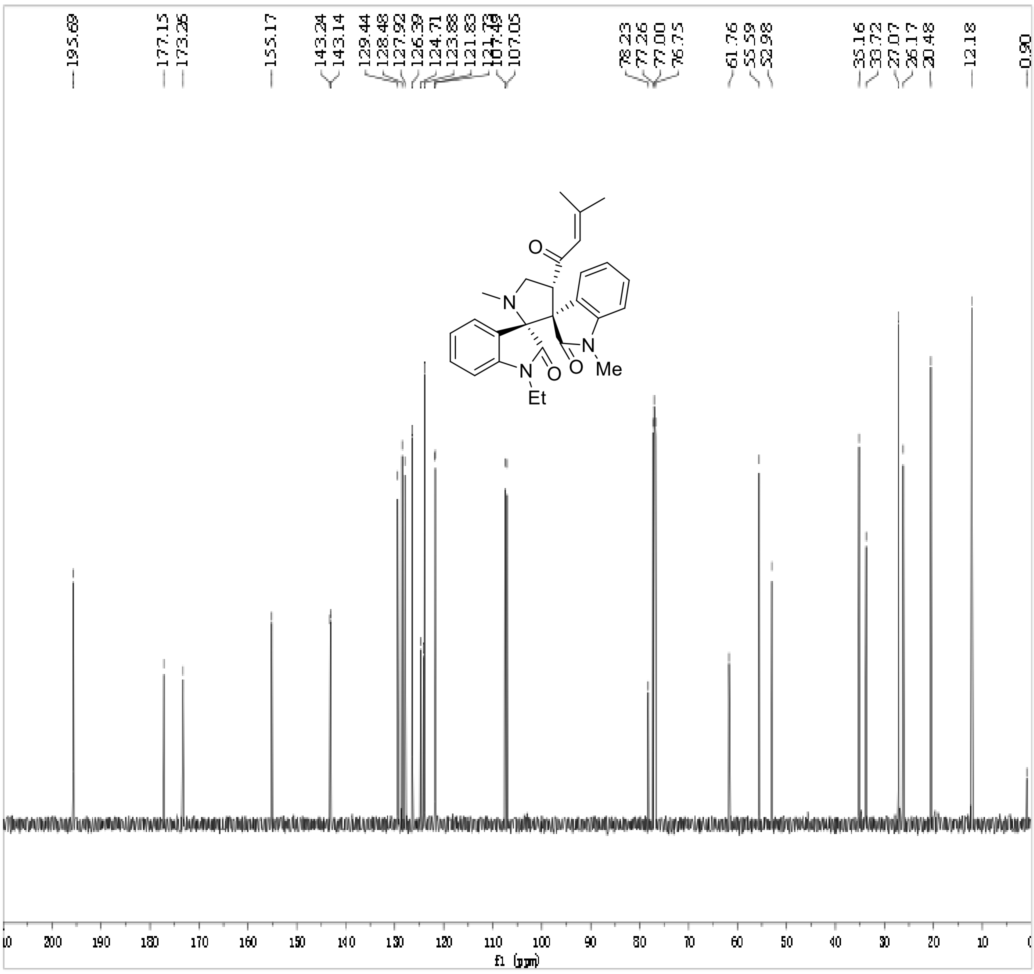

^{1}H and ^{13}C NMR of 5oa

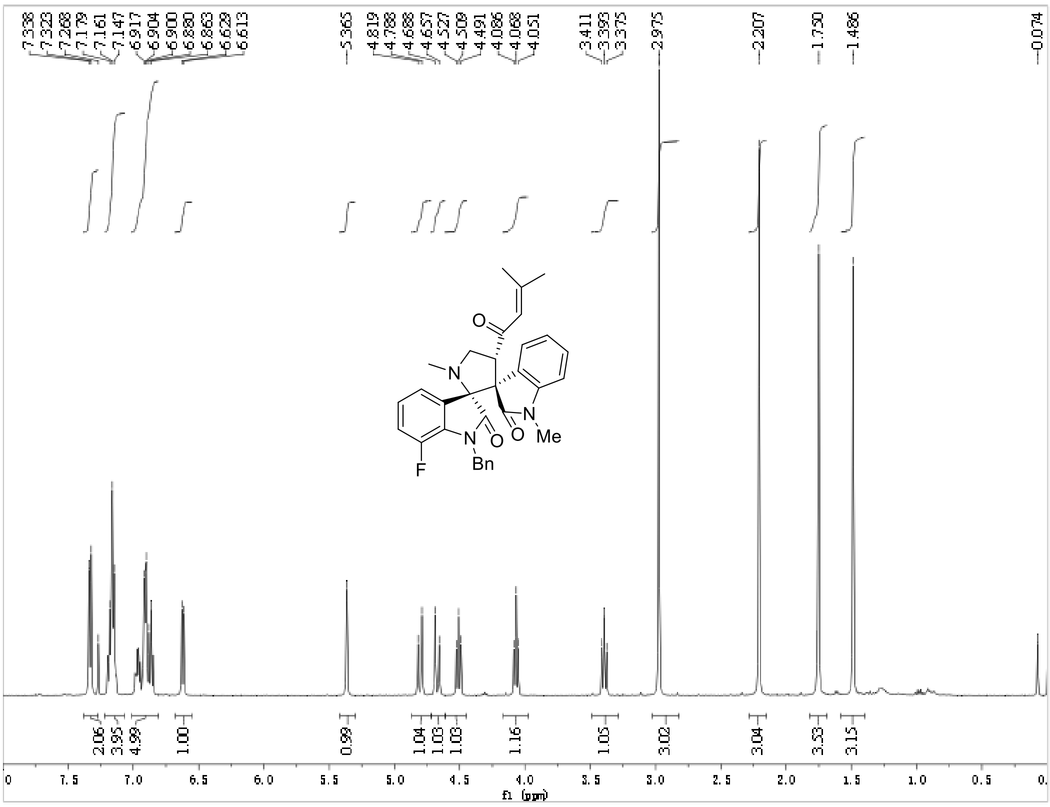

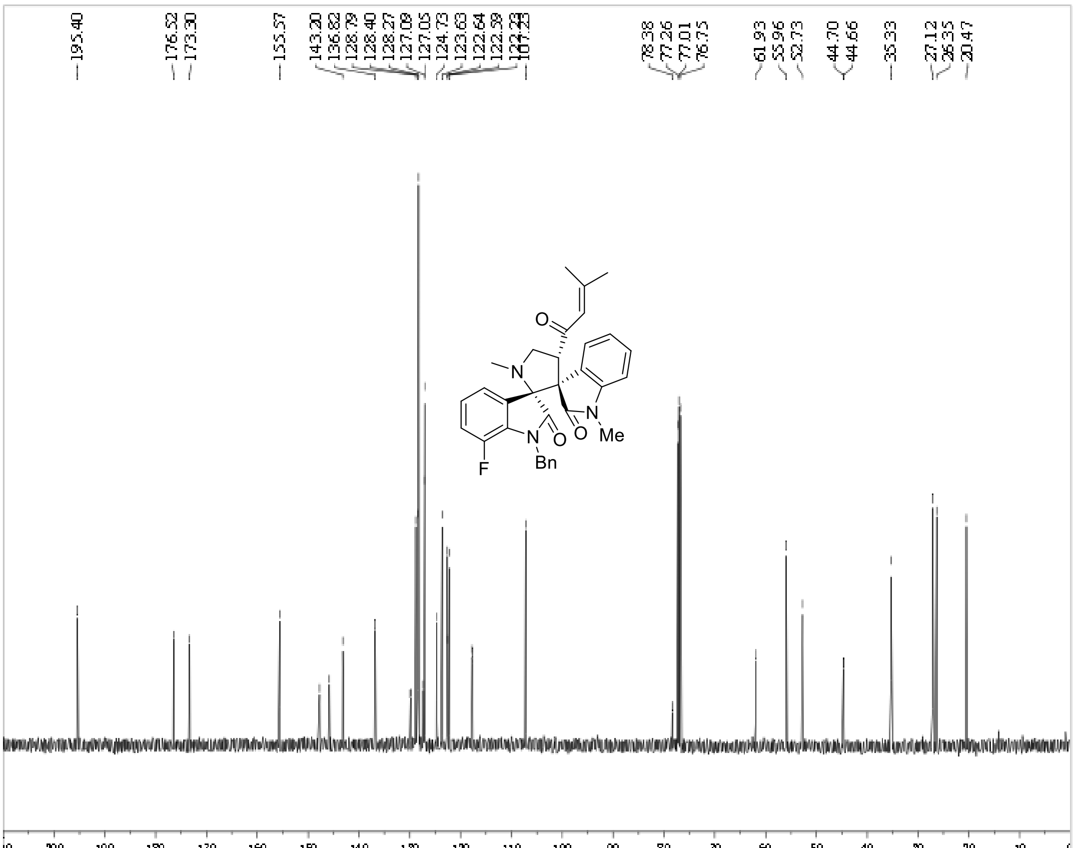

^{1}H and ^{13}C NMR of 5pa

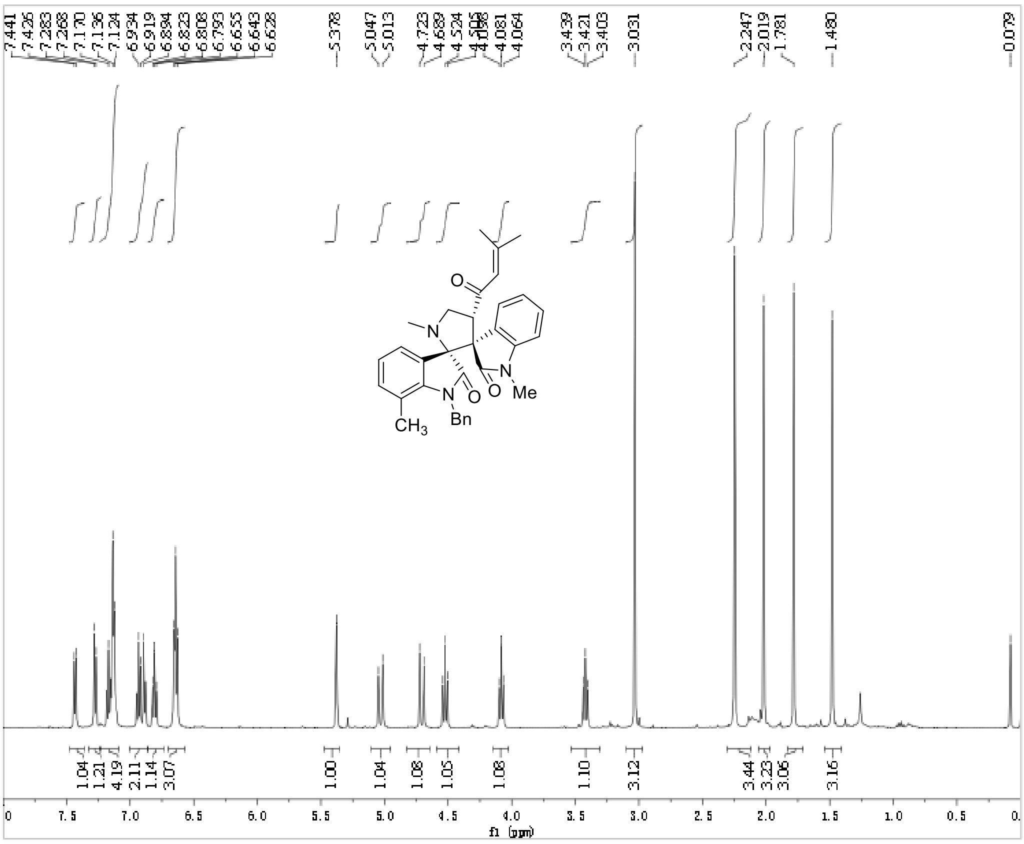

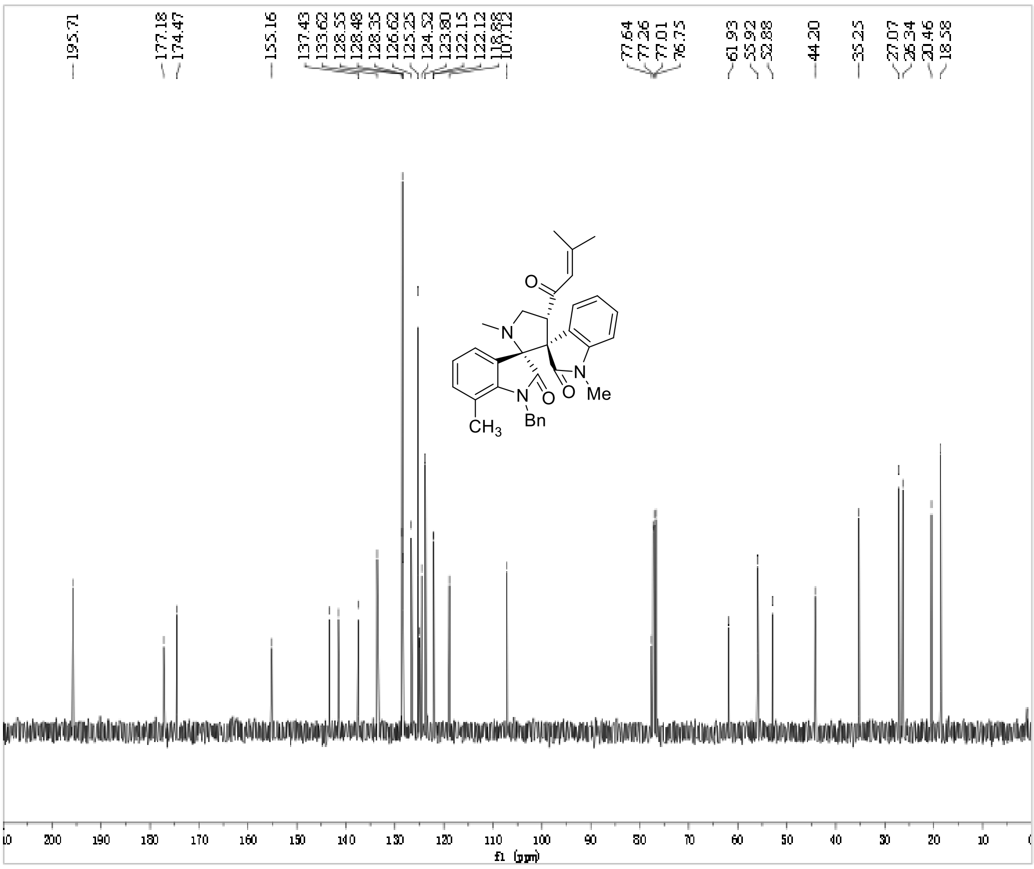

^{1}H and ^{13}C NMR of 5ai

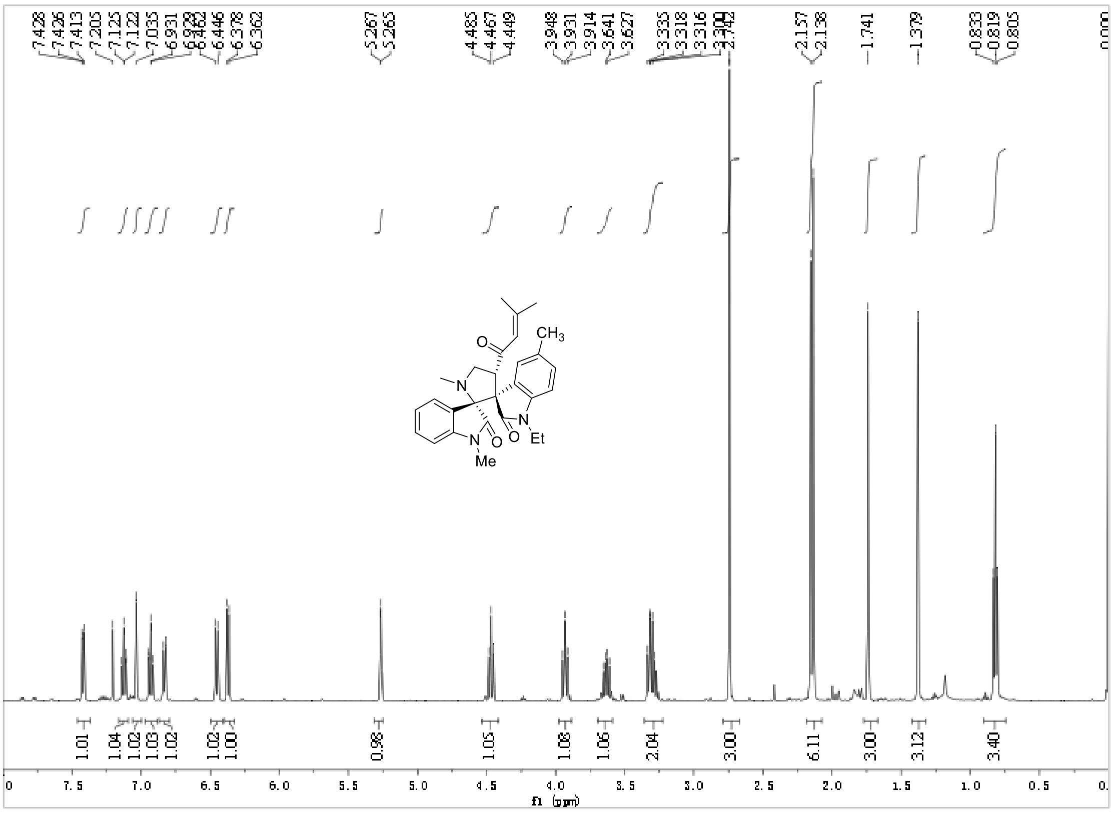

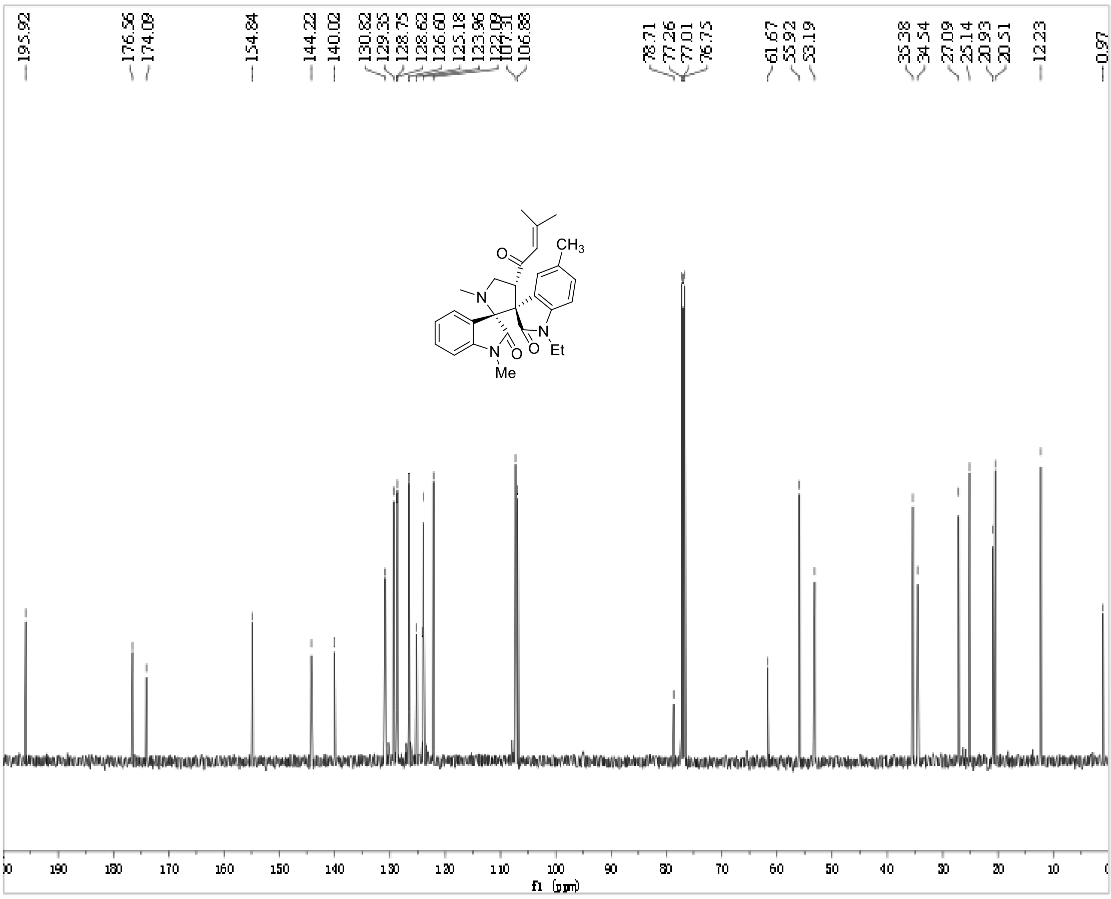

^{1}H and ^{13}C NMR of 5aj

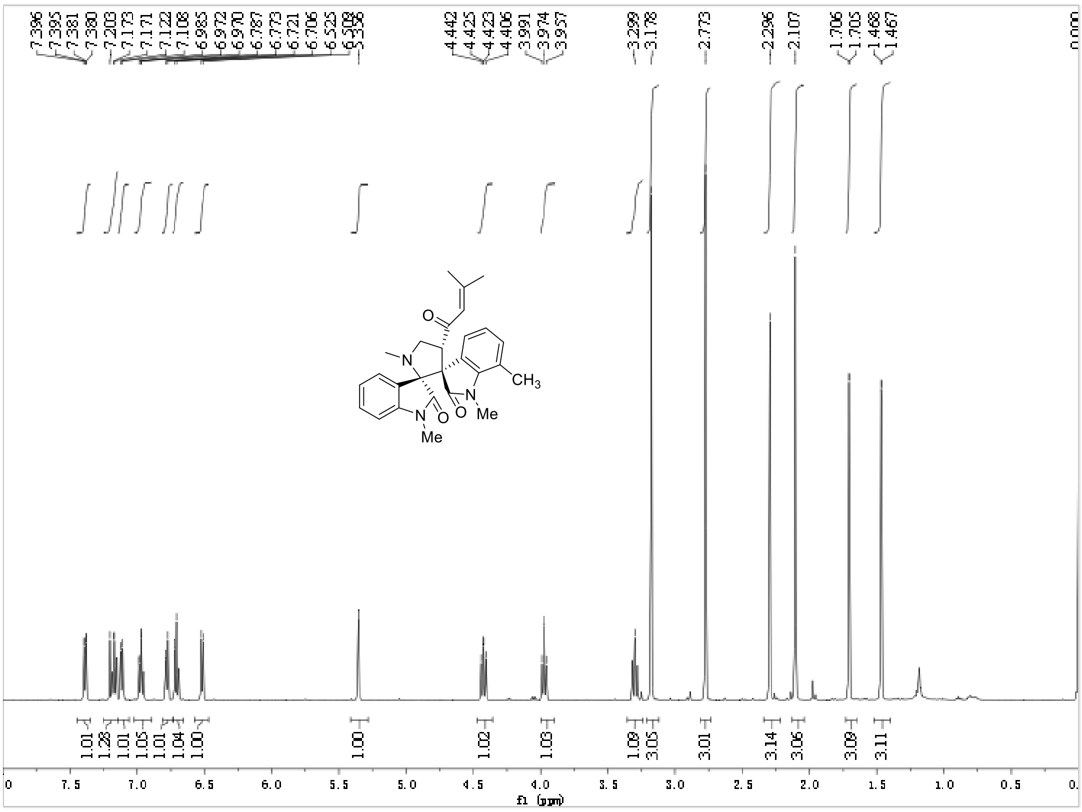

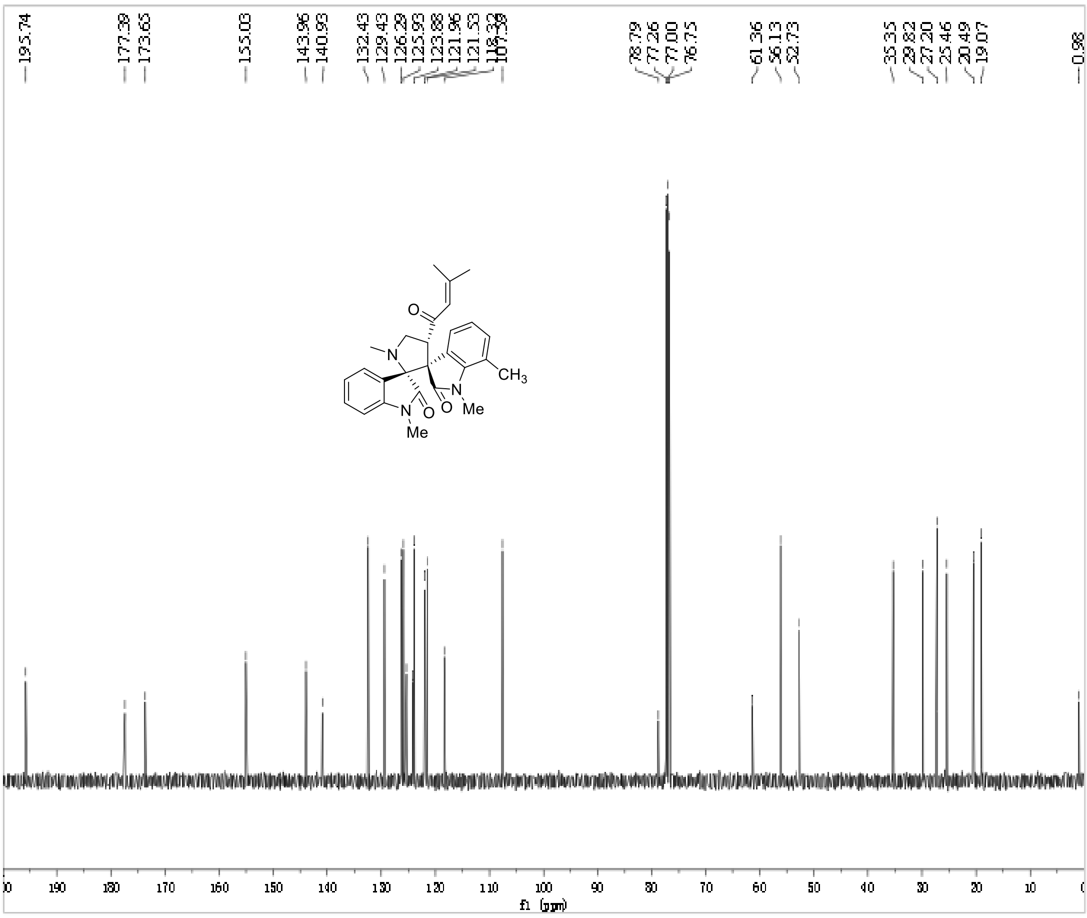

^{1}H and ^{13}C NMR of 5dh

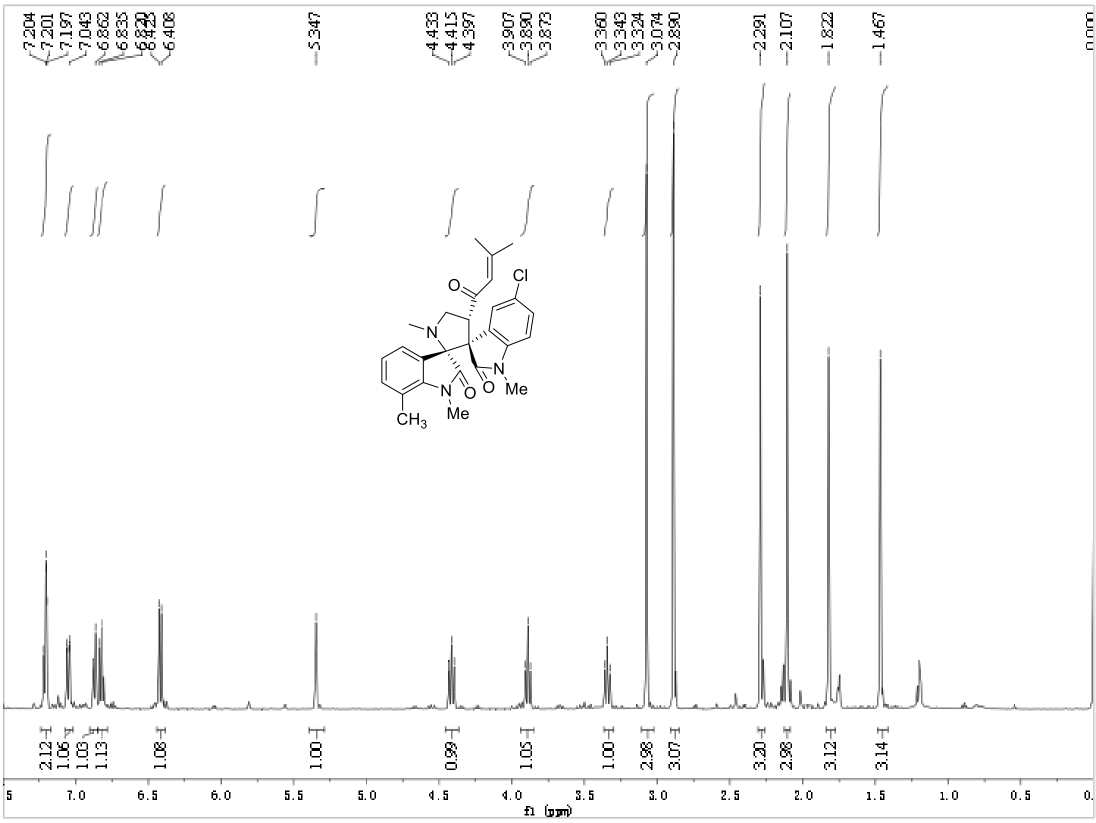

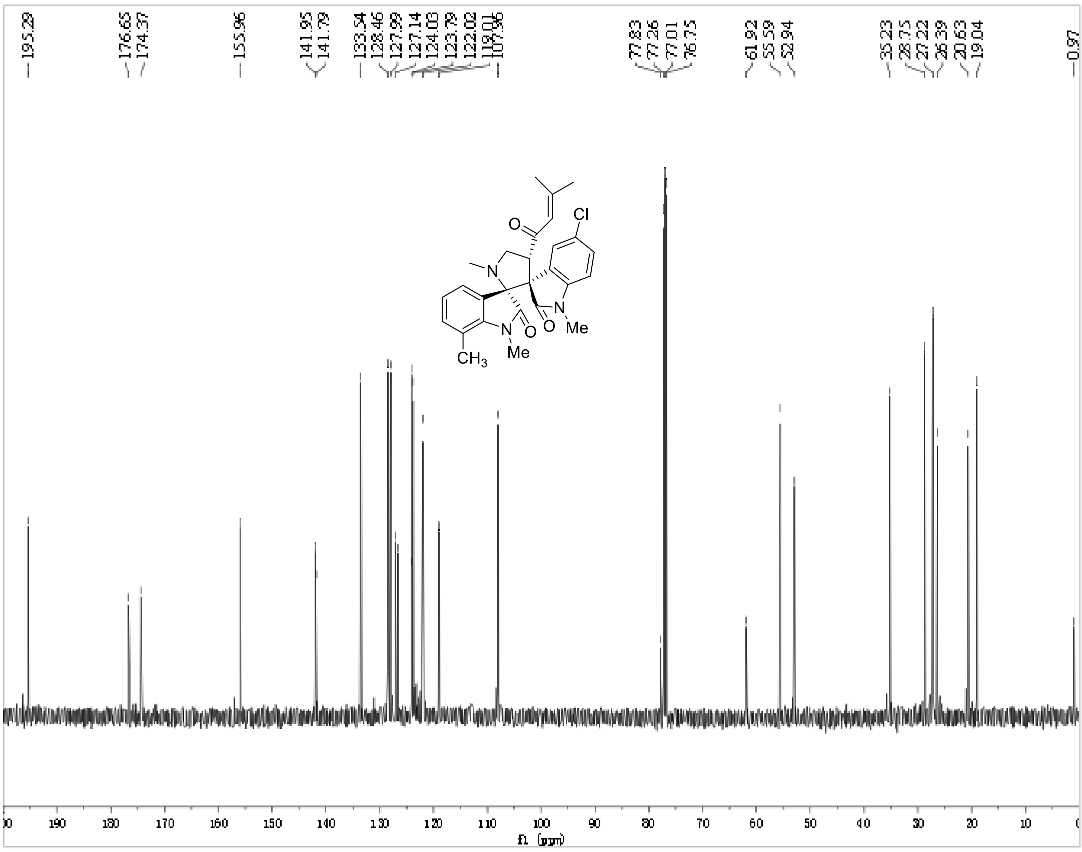

^{1}H and ^{13}C NMR of 5nb

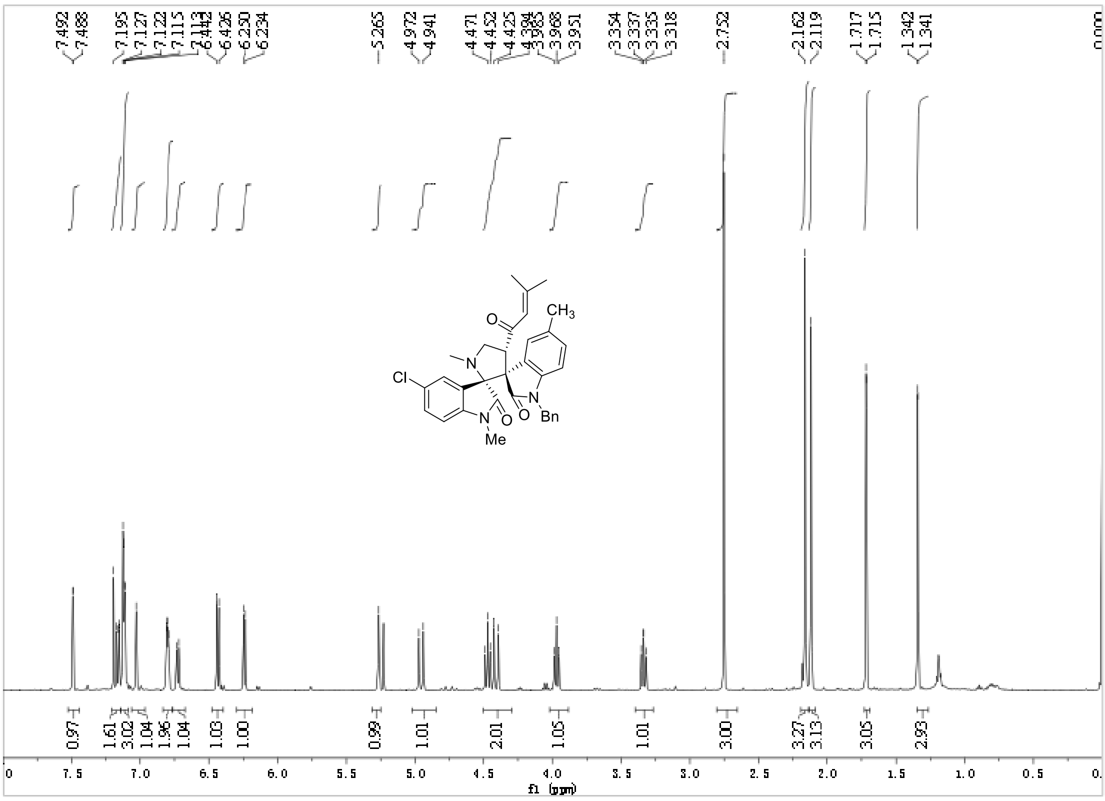

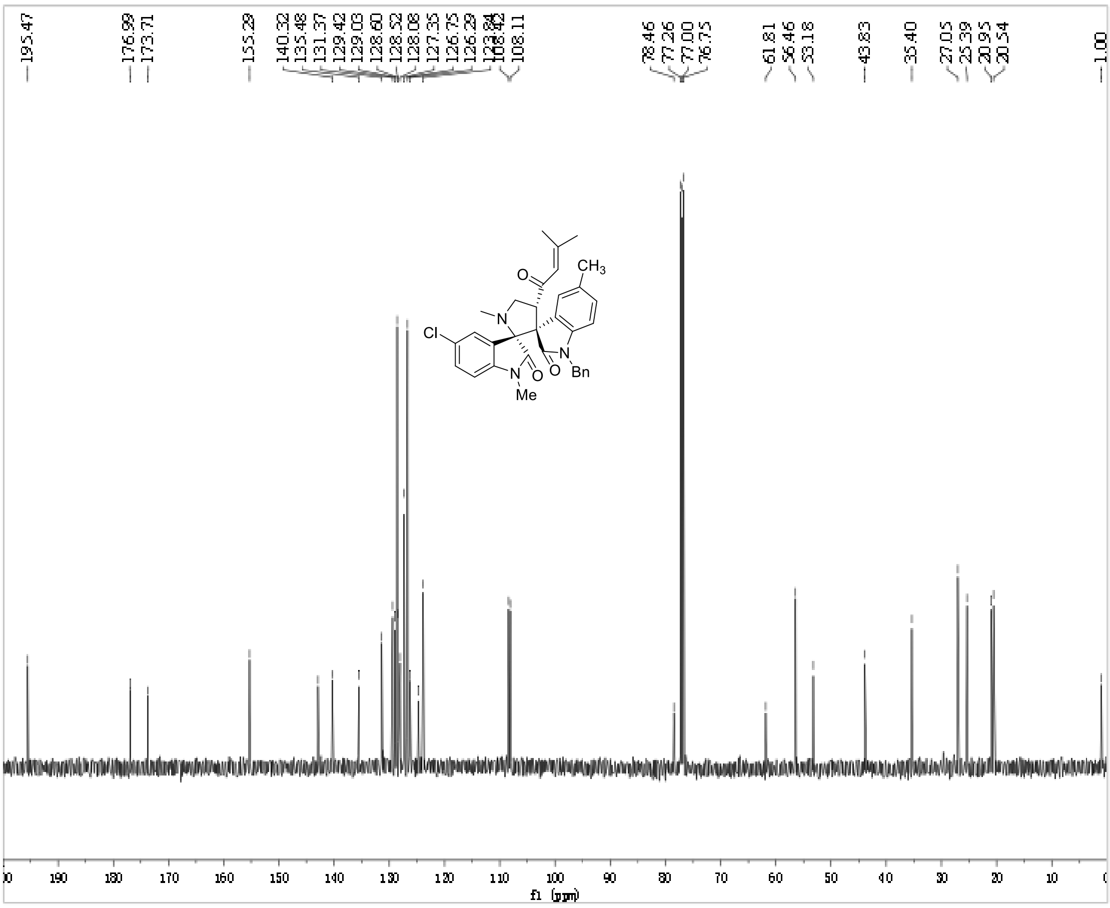

^{1}H and ^{13}C NMR of 5oc

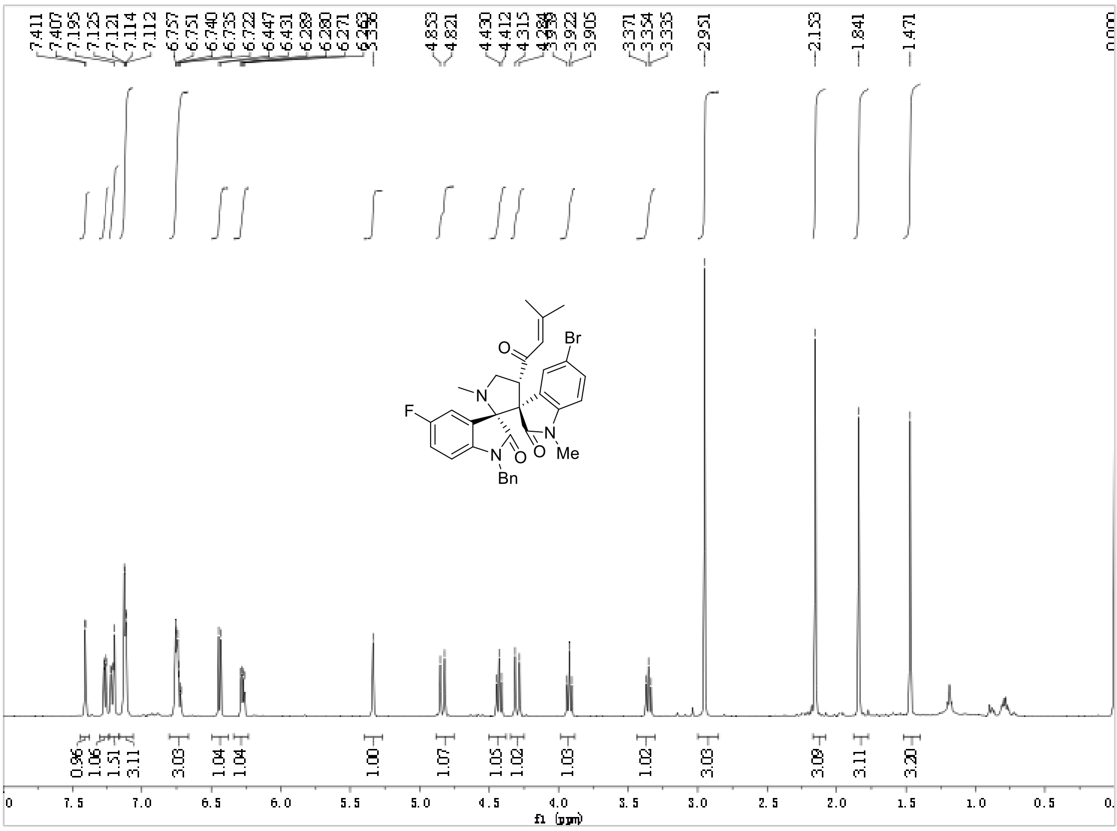

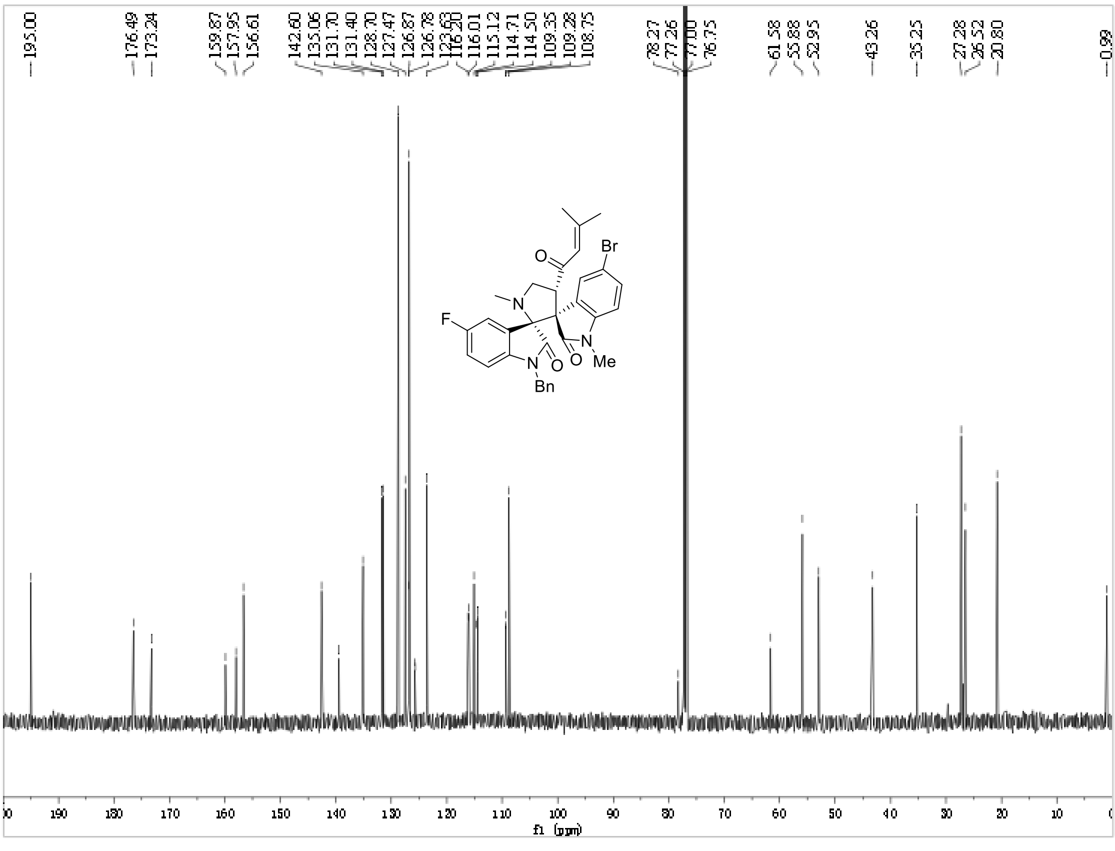

Supplement: Supplementary file 1 [file molecules-22-00645-s001.pdf]
